# Supplementary material for: Cost-utility of cytisine for smoking cessation over and above behavioural support in people with newly diagnosed pulmonary tuberculosis: an economic evaluation of a multicentre randomised controlled trial
Source: BMJ Open. 2022 Aug 26;12(8):e049644. doi: 10.1136/bmjopen-2021-049644 (PMC9422837; doi:10.1136/bmjopen-2021-049644)
Supplement: Supplementary data [file bmjopen-2021-049644supp001.pdf]

# Detailed methods and results of secondary analyses

## Methods

### Cytisine dosage schedule

The standard regimen for cytisine (Desmoxan, Aflofarm) was a 25-day course with 1.5mg hard capsules for oral administration, with six per day on days 1-3, five per day on days 4-12, four per day on days 13-16, three per day on days 17-20, two per day on days 21-24 and one on the last day.

### Intervention costs

Training for the delivery of brief behavioural support was given to TB health workers before the trial began. In Bangladesh, it was a one-day training programme with a one-day refresher training and the total cost was estimated to be €4499 in 2017. In Pakistan, this consisted of a two-day training programme for DOTS facilitators and the total cost was estimated at €2324 in 2016.

In Bangladesh, the average monthly salary of a TB health worker (local salary grades G-11 to G-13) was PPP US$649.84, and average working hours per week was 48 hours.[1] In Pakistan, the average monthly salary of a TB health worker was PPP US$921.50 and average working hours per week was 47.4 hours.[2] We assumed a 30-day month as 4.3 weeks. The estimated hourly wage was therefore PPP US$3.17 in Bangladesh and PPP US$4.54 in Pakistan. The cost of BS was PPP US$0.52 for the first session and PPP US$0.26 for the second session in Bangladesh and PPP US$0.75 and PPP US$0.38 in Pakistan.

### TB treatment costs

The standard treatment for pulmonary TB consisted of a two-month intensive phase and a four-month continuation phase. We extracted the overall costs of a six-month TB treatment for the two countries from the World Health Organization (WHO) TB database[3] and applied a ratio of costs of the two phases, based on a TB treatment modelling study,[4] to produce an estimate of monthly cost of intensive phase and continuation phase respectively. They were then converted to PPP US$.[5 6] The TB treatment costs were then estimated based on the participants' treatment progression on their TB registry cards.

### Smoking cessation costs outside of the trial

Due to the limited smoking cessation services in the two countries,[7 8] we made assumptions on duration, based on usual practice in the UK:[9 10] 10-minute brief intervention with professionals (physician or professional nurse) for help/advice from a public/government clinic/hospital; one hour group session of 15 people or 30-minute individual session led by medical technicians/auxiliary nurses for counselling sessions in public/voluntary hospital. The ratio of group and individual sessions was assumed to be 1:1. The average hourly wage was PPP US$4.14 for "professionals" and PPP US$3.33 for "technicians and associate professionals" in Bangladesh, and PPP US$5.29 for "professionals" and PPP US$4.51 for "technicians and associate professionals" in Pakistan.[1 2 5]

#### *General healthcare services costs*

Participants' visits to a public/voluntary doctor and length of stay in a public hospital in the previous six months were collected by self-report at baseline, 6- and 12-month follow-ups. The unit costs of these services were extracted from the WHO country specific in- and out-patient costs, inflated to 2018 and converted to PPP US$.[5 11 12] The unit cost of hospital inpatient stay was the average of all hospital levels and the unit cost of a visit to doctor was the average of all settings for outpatient. These costs did not include drugs.

### Out-of-pocket payments (OOPs)

Participants' spending related to following items were collected: TB treatment, public/voluntary doctor and hospital visits, and private doctor and hospital visits, including travel, smoking cessation services in public/voluntary facilities and private settings, purchasing Nicotine Replacement Therapy (NRT) or e-cigarette refills, purchasing other traditional medicine for quitting, and purchasing tobacco products.

## Results

### Costs

Mean training costs were PPP US$10.94 (SD PPP US$2.09) per participant in the cytisine arm and PPP US$10.92 (SD PPP US$2.09) per participant in the placebo arm. Mean cost of the information leaflet was PPP US$0.76 (SD PPP US$0.75) in the cytisine arm and PPP US$0.75 (SD PPP US$0.75) in the placebo arm. Mean cost of BS was PPP US$0.68 (SD PPP US$0.36) among 1233 participants in the cytisine arm and PPP US$0.70 (SD US$0.36) among 1226 participants in the placebo arm. Mean cost of cytisine was PPP US$48.27 (SD PPP US$12.54) while the cost of placebo was assumed at zero.

Mean costs of TB treatment were estimated to be PPP US$307.39 (SD PPP US$110.25) in the cytisine arm and PPP US$302.45 (SD PPP US$108.53) in the placebo arm, excluding 102 (8.2%) participants in the cytisine arm and 103 (8.4%) in the placebo arm who did not have information from TB cards at six-month follow-up (Table 1). The use of smoking cessation support was reported by a small group of participants in both arms. Mean costs of public/voluntary smoking cessation services were low in both arms throughout the 12 months period. Most participants reported neither visiting a doctor other than for their TB treatment nor being admitted to hospital for any reason. While mean costs of doctor visits were similar between respondents in both arms throughout the trial period, mean costs of hospital stay in the cytisine arm were nearly twice as high as in the placebo arm in months 1-6.

### Out-of-pocket payments

The respondents reported an increase of spending on smoking cessation in months 1-6 compared to close to none before and after, corresponding with the intervention delivery and TB treatment period. Mean spending on tobacco was lower during the trial period than before among respondents. However, in comparison with the spending on smoking cessation, the spending on tobacco was consistently higher. The OOPs for healthcare services, including travel, loosely followed the same pattern of the costs of the services (Table 1).

*Table 1 Mean (SD) costs and OOPs of TB treatment, additional smoking cessation services and general healthcare services, and OOPs on tobacco products, by arm*

| | **Cytisine (n=1239)** | | **Placebo (n=1233)** | |
|---|---|---|---|---|
| | **n** | **Mean (SD) PPP US$** | **n** | **Mean (SD) PPP US$** |
| **TB treatment costs** | | | | |
| TB registry | 1137 | 307.39 (110.25) | 1130 | 302.45 (108.53) |
| **Additional smoking cessation costs** | | | | |
| Six months before baseline | 1239 | 0.00 (0.10) | 1233 | 0.00 (0.09) |
| Months 1-6 | 1174 | 0.47 (1.17) | 1164 | 0.47 (1.11) |
| Months 7-12 | 1134 | 0.22 (0.75) | 1144 | 0.21 (0.77) |
| **Doctor visit costs** | | | | |
| Six months before baseline | 1239 | 3.26 (14.27) | 1232 | 3.48 (23.44) |
| Months 1-6 | 1176 | 3.39 (12.96) | 1166 | 3.04 (10.39) |

| | | | | |
|---|---|---|---|---|
| Months 7-12 | 1148 | 1.27 (4.73) | 1157 | 1.12 (4.58) |
| **Hospital stay costs** | | | | |
| Six months before baseline | 1237 | 6.77 (57.79) | 1231 | 4.84 (43.25) |
| Months 1-6 | 1175 | 31.58 (268.99) | 1166 | 16.52 (148.33) |
| Months 7-12 | 1148 | 5.01 (80.30) | 1157 | 5.87 (94.86) |
| **Additional smoking cessation OOPs** | | | | |
| Six months before baseline | 1236 | 0.04 (0.75) | 1230 | 0.00 (0.09) |
| Months 1-6 | 1091 | 0.34 (2.72) | 1080 | 0.28 (1.95) |
| Months 7-12 | 1110 | 0.05 (0.61) | 1115 | 0.05 (0.56) |
| **Tobacco OOPs** | | | | |
| Six months before baseline | 1229 | 1.79 (5.05) | 1224 | 1.64 (2.35) |
| Months 1-6 | 1177 | 0.50 (1.03) | 1166 | 0.48 (0.91) |
| Months 7-12 | 1148 | 0.58 (0.92) | 1157 | 0.57 (0.75) |
| **TB treatment OOPs** | | | | |
| Six months before baseline | 1238 | 15.45 (59.42) | 1233 | 19.71 (119.96) |
| Months 1-6 | 1174 | 22.00 (85.28) | 1164 | 15.77 (42.34) |
| Months 7-12 | 1148 | 5.03 (48.72) | 1156 | 4.36 (30.50) |
| **Doctor visit OOPs** | | | | |
| Six months before baseline | 1233 | 61.53 (243.17) | 1227 | 63.21 (199.10) |
| Months 1-6 | 1173 | 27.49 (238.38) | 1158 | 22.07 (216.35) |
| Months 7-12 | 1148 | 13.28 (84.58) | 1157 | 19.07 (162.71) |
| **Hospital stay OOPs** | | | | |
| Six months before baseline | 1237 | 6.91 (101.21) | 1231 | 3.01 (28.19) |
| Months 1-6 | 1173 | 16.65 (200.58) | 1164 | 11.72 (220.92) |
| Months 7-12 | 1148 | 17.20 (460.84) | 1157 | 5.65 (99.21) |

## Productivity loss

Among the respondents, while the mean productivity loss peaked in months 1-6 as expected, it was higher than expected in the six months before baseline, most prominently reflected by productivity loss due to participants' sick leave (Table 2). This might correspond with productivity loss due to companion to TB clinic in the six months before baseline, which was consistent with participants' OOPs for TB clinic during the same period.

*Table 2 Mean (SD) productivity loss of companion to TB clinic, doctor, and participants' sick leave, by arm*

| | **Cytisine (n=1239)** | | **Placebo (n=1233)** | |
|---|---|---|---|---|
| | **n** | **Mean (SD) PPP US$** | **n** | **Mean (SD) PPP US$** |
| **Companion to TB clinic** | | | | |
| Six months before baseline | *1232* | *4.62 (9.01)* | *1228* | *4.48 (7.73)* |
| Month 1 – 6 | *1134* | *13.45 (21.55)* | *1127* | *12.43 (19.86)* |
| Month 7 – 12 | *1145* | *2.01 (7.40)* | *1152* | *2.33 (7.19)* |
| **Companion to doctor** | | | | |
| Six months before baseline | *1203* | *2.10 (9.15)* | *1196* | *1.87 (6.05)* |
| Month 1 – 6 | *1126* | *3.35 (13.22)* | *1116* | *2.65 (8.44)* |
| Month 7 – 12 | *1143* | *0.37 (2.82)* | *1151* | *0.56 (4.82)* |
| **Sick leave** | | | | |
| Six months before baseline | *1230* | *27.14 (73.17)* | *1227* | *23.82 (61.12)* |
| Month 1 – 6 | *1194* | *31.98 (100.27)* | *1171* | *28.52 (107.49)* |

| | Cytisine (n=1239) | | Placebo (n=1233) | |
|---|---|---|---|---|
| | n | Mean (SD) PPP US$ | n | Mean (SD) PPP US$ |
| Month 7 – 12 | *1163* | *3.62 (18.24)* | *1160* | *5.14 (28.21)* |

## Quality-adjusted life years

In the EQ-5D-5L descriptive system, the domains with least proportion of respondents scoring no problem were Pain/Discomfort and Anxiety/Depression at all three time points although the proportion increased after baseline (Table 3).

*Table 3 Number and percentage of respondents scoring five levels of each domain of EQ-5D-5L, by arm and time point*

| Domains | Mobility | | Self-care | | Usual activities | | Pain/ Discomfort | | Anxiety/ Depression | |
|---|---|---|---|---|---|---|---|---|---|---|
| | Cytisine | Placebo | Cytisine | Placebo | Cytisine | Placebo | Cytisine | Placebo | Cytisine | Placebo |
| Baseline | | | | | | | | | | |
| 1 | 731 | 746 | 985 | 993 | 655 | 654 | 413 | 426 | 407 | 411 |
| | 59% | 61% | 79% | 81% | 53% | 53% | 33% | 35% | 33% | 33% |
| 2 | 315 | 291 | 190 | 163 | 380 | 373 | 447 | 462 | 453 | 463 |
| | 25% | 24% | 15% | 13% | 31% | 30% | 36% | 38% | 37% | 38% |
| 3 | 140 | 143 | 49 | 58 | 133 | 146 | 250 | 227 | 232 | 231 |
| | 11% | 12% | 4% | 5% | 11% | 12% | 20% | 18% | 19% | 19% |
| 4 | 50 | 51 | 12 | 16 | 55 | 52 | 114 | 104 | 112 | 98 |
| | 4% | 4% | 1% | 1% | 4% | 4% | 9% | 8% | 9% | 8% |
| 5 | 3 | 2 | 3 | 1 | 14 | 8 | 14 | 13 | 33 | 29 |
| | 0% | 0% | 0% | 0% | 1% | 1% | 1% | 1% | 3% | 2% |
| Total | 1239 | 1233 | 1239 | 1231 | 1237 | 1233 | 1238 | 1232 | 1237 | 1232 |
| Six months | | | | | | | | | | |
| 1 | 985 | 992 | 1077 | 1078 | 945 | 960 | 753 | 778 | 818 | 829 |
| | 86% | 88% | 94% | 95% | 83% | 85% | 66% | 69% | 72% | 73% |
| 2 | 119 | 116 | 56 | 44 | 171 | 147 | 364 | 325 | 287 | 260 |
| | 10% | 10% | 5% | 4% | 15% | 13% | 32% | 29% | 25% | 23% |
| 3 | 25 | 13 | 7 | 8 | 19 | 18 | 19 | 20 | 28 | 29 |
| | 2% | 1% | 1% | 1% | 2% | 2% | 2% | 2% | 2% | 3% |
| 4 | 12 | 10 | 2 | 2 | 5 | 5 | 6 | 7 | 8 | 12 |
| | 1% | 1% | 0% | 0% | 0% | 0% | 1% | 1% | 1% | 1% |
| 5 | 2 | 1 | 1 | 0 | 3 | 1 | 1 | 1 | 2 | 1 |
| | 0% | 0% | 0% | 0% | 0% | 0% | 0% | 0% | 0% | 0% |
| Total | 1143 | 1132 | 1143 | 1132 | 1143 | 1131 | 1143 | 1131 | 1143 | 1131 |
| 12 months | | | | | | | | | | |
| 1 | 994 | 1020 | 1059 | 1082 | 968 | 996 | 755 | 780 | 826 | 833 |
| | 90% | 91% | 96% | 97% | 88% | 89% | 69% | 70% | 75% | 75% |
| 2 | 86 | 75 | 33 | 24 | 115 | 101 | 299 | 284 | 226 | 238 |
| | 8% | 7% | 3% | 2% | 10% | 9% | 27% | 26% | 21% | 21% |
| 3 | 11 | 12 | 6 | 2 | 12 | 10 | 35 | 34 | 33 | 28 |

| | | | | | | | | | | |
|---|---|---|---|---|---|---|---|---|---|---|
| | 1% | 1% | 1% | 0% | 1% | 1% | 3% | 3% | 3% | 3% |
| 4 | 8 | 6 | 3 | 4 | 4 | 5 | 9 | 13 | 8 | 13 |
| | 1% | 1% | 0% | 0% | 0% | 0% | 1% | 1% | 1% | 1% |
| 5 | 3 | 2 | 1 | 1 | 3 | 1 | 3 | 2 | 4 | 1 |
| | 0% | 0% | 0% | 0% | 0% | 0% | 0% | 0% | 0% | 0% |
| Total | 1102 | 1115 | 1102 | 1113 | 1102 | 1113 | 1101 | 1113 | 1097 | 1113 |

Levels for each domain: 1=no problem, 2=slight problem, 3=moderate problem, 4=severe problem, 5=extreme problem/inability

Table 4 shows mean EQ-5D-5L utility and VAS among observed cases at baseline, 6 and 12 months follow-ups and QALYs over 6 and 12 months period. Mean utility in the cytisine arm appeared to be consistently lower than in the placebo arm at all timepoints though the difference was small. The mean QALYs were therefore lower in the cytisine arm than in the placebo arm. However, it should be noted, only those who had data on all relevant timepoints were included in calculating QALYs. The EQ-5D VAS showed a similar pattern where both arms began at similar level but in the cytisine arm, the observed cases scored slightly lower than those in the placebo arm in the follow-ups.

*Table 4 Mean (SD) EQ-5D-5L utility, EQ-5D VAS and QALYs, by arm*

| | **Cytisine (n=1239)** | | **Placebo (n=1233)** | |
|---|---|---|---|---|
| | **n** | **Mean (SD)** | **n** | **Mean (SD)** |
| **Utility** | | | | |
| Baseline | 1234 | 0.754 (0.133) | 1229 | 0.759 (0.130) |
| 6 months | 1179 | 0.825 (0.165) | 1164 | 0.831 (0.161) |
| 12 months | 1144 | 0.822 (0.189) | 1149 | 0.829 (0.176) |
| **QALYs** | | | | |
| Over 6 months | 1174 | 0.394 (0.056) | 1160 | 0.397 (0.054) |
| Over 12 months | 1129 | 0.805 (0.134) | 1122 | 0.810 (0.128) |
| **VAS** | | | | |
| Baseline | 1239 | 53.5 (15.4) | 1233 | 53.5 (16.0) |
| 6 months | 1179 | 80.5 (20.3) | 1165 | 81.3 (19.8) |
| 12 months | 1150 | 84.0 (21.8) | 1156 | 84.7 (20.7) |

## Cost-utility analysis by country

The mean costs of smoking cessation and healthcare services in the six months before baseline were PPP US$18.33 (SE PPP US$3.65) in Pakistan and PPP US$5.40 (SE PPP US$1.65) in Bangladesh in the cytisine arm. In the placebo arm, the mean costs of these two types of services were PPP US$16.35 (SE PPP US$3.55) in Pakistan and PPP US$3.72 (SE PPP US$0.55) in Bangladesh.

The mean costs of intervention were PPP US$74.37 (SE PPP US$0.68) in the cytisine arm and PPP US$15.84 (SE PPP US$0.03) in the placebo arm in Pakistan. The mean costs of intervention were PPP US$52.10 (SE PPP US$0.13) in the cytisine arm and PPP US$10.23 (SE PPP US$0.00) in the placebo arm in Bangladesh.

The mean costs of TB treatment in the two arms were on a similar level within each country, over PPP US$400 in Pakistan and over PPP US$200 in Bangladesh. The mean costs of doctor visits were very similar between arms in Bangladesh, but they were slightly higher in the cytisine arm in Pakistan (PPP US$3.17 vs PPP US$2.66). The most prominent difference was in the mean costs of

hospital stay. In Pakistan, the mean costs of hospital stay were considerably higher in the cytisine arm (PPP US$78.12 [SE PPP US$19.80]) than in the placebo arm (PPP US$32.70 [SE PPP US$9.76]). On the contrary, in Bangladesh, the mean costs of hospital stay in the placebo arm (PPP US$7.35 [SE PPP US$3.82]) were over twice as high as in the cytisine arm (PPP US$3.07 [SE PPP US$1.62]). The mean costs of smoking cessation services were not different between arms within each country. However, there were nearly null costs incurred in Pakistan.

Upon further investigation, more participants had hospital stays in Pakistan than in Bangladesh, regardless of which arm they were in. Among participants who incurred hospital stay costs over the six months post-randomisation, not only did the cytisine arm in Pakistan have more participants admitted to hospital but also showed a few potential outliers (Figure 1). This was in contrast with the placebo arm in Pakistan and both arms in Bangladesh.

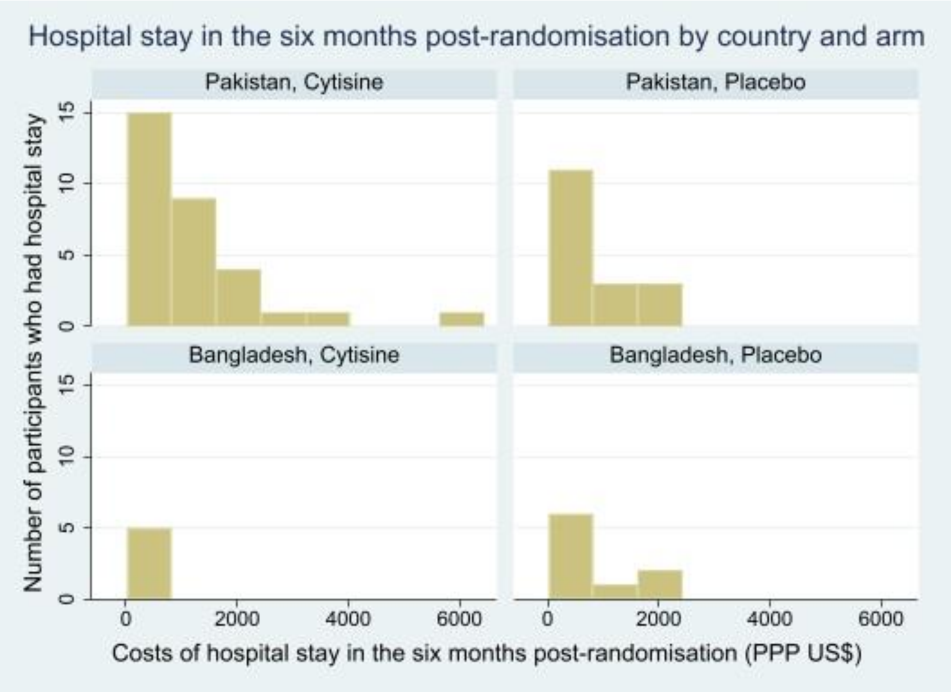


*Figure 1 Distribution of costs of hospital stay among those who incurred this cost, by country and arm*

Whilst the mean utility was higher in Pakistan than in Bangladesh, the mean utility in both arms showed a relatively gradual and small increase from baseline to six months (Figure 2). In contrast, the mean utility at baseline was much lower in Bangladesh than in Pakistan but it increased more sharply to a similar level in the cytisine arm and a higher level in the placebo arm.

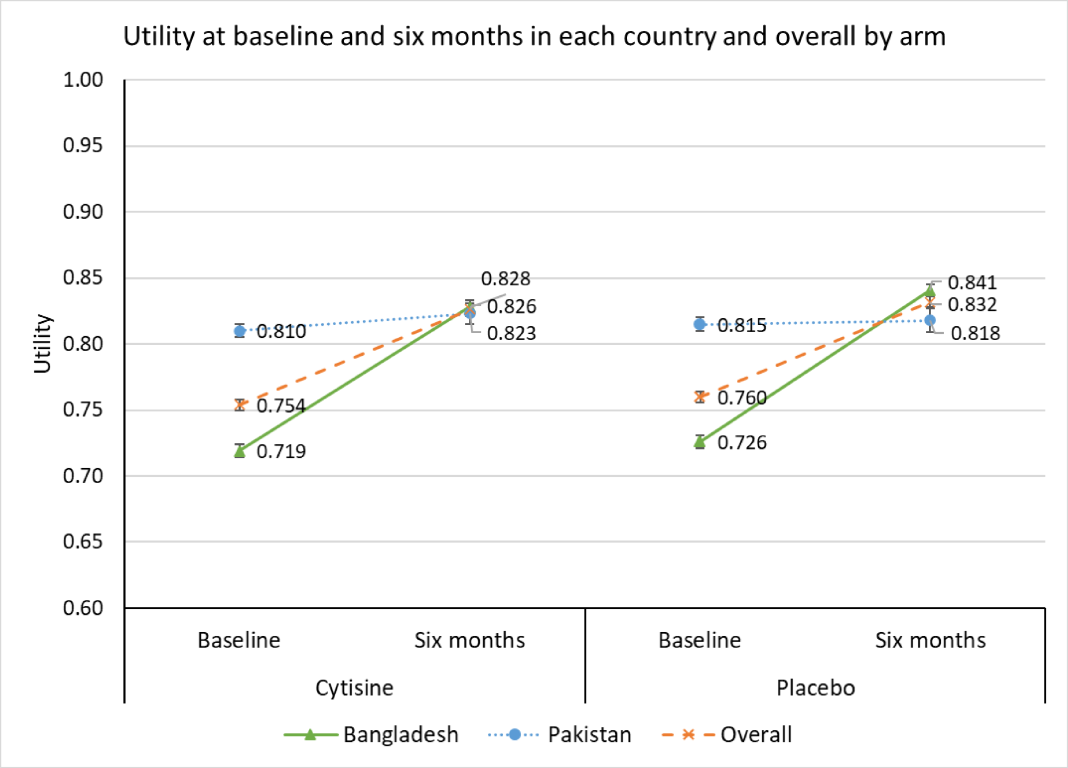


*Figure 2 Mean utility at baseline and six months by country and by arm*

Adjusting for costs of healthcare and smoking cessation services in the six months before baseline, age, gender, with sites as random effect, the incremental costs over the six months post randomisation were PPP US$108.46 (95%CI PPP US$69.69 to PPP US$157.88) in Pakistan and PPP US$37.06 (95% CI PPP US$28.12 to PPP US$43.85) in Bangladesh (Table 5). Adjusting for utility at baseline, age, gender, with sites as random effect, the incremental QALYs were 0.001 (95% CI -0.004 to 0.008) in Pakistan and -0.003 (95% CI -0.006 to 0.000) in Bangladesh. Therefore, in Pakistan, the ICER was calculated in at PPP US$108,464 per QALY and in Bangladesh, the cytisine arm was dominated by the placebo arm (the cytisine arm being more costly but less effective). Figure 3 shows the uncertainty surrounding the ICERs estimated using bootstrap technique. For Bangladesh, 96% (4794/5000) of the bootstrapped replicates fell in the north-west quadrant of the CEP, where the intervention was more costly but less effective in terms of QALYs. This supports the point estimate that the cytisine arm was dominated by the placebo arm. For Pakistan, 71% (3568/5000) of the bootstrapped replicates fell in the north-east quadrant of the CEP, where the intervention was more costly and more effective in terms of QALYs. The rest fell in the north-west quadrant, indicating a more costly but less effective intervention. According to the estimate made by Woods et al., the willingness-to-pay (WTP) threshold for Pakistan was PPP US$314 to PPP US$2146 per QALY in 2013 [13]. Converting to Pakistan Rupees in 2013 then inflating using consumer price index to 2018 [5 14], the estimated WTP in Pakistan was PPP US$356 to PPP US$2431 per QALY. Represented by the red line in Figure 3, it was apparent that none of the estimates fell under the upper boundary of the WTP (i.e. not cost-effective), same as the point estimate of PPP US$108,464 per QALY. The probability of the cytisine intervention being cost-effective was 0% throughout a wide range of WTP values in both

countries, the CEACs were therefore not presented. By these results, the cytisine intervention was unlikely to be cost-effective, comparing with placebo, in either Pakistan or Bangladesh.

*Table 5 Cost-utility analysis results by country (1 PPPUS$ = 30.9 Bangladeshi Taka = 29.3 Pakistani Rupees)*

| **Costs (PPP US$) Mean (SE)** | **Pakistan** | | **Bangladesh** | |
|---|---|---|---|---|
| | **Cytisine (n=476)** | **Placebo (n=469)** | **Cytisine (n=763)** | **Placebo (n=764)** |
| Intervention | 74.37 (0.68) | 15.84 (0.03) | 52.10 (0.13) | 10.23 (0.00) |
| TB treatment | 421.30 (5.43) | 412.97 (5.84) | 232.69 (0.78) | 233.59 (0.61) |
| Doctor visit | 3.17 (0.82) | 2.66 (0.65) | 3.50 (0.29) | 3.37 (0.29) |
| Hospital stay | 78.12 (19.80) | 32.70 (9.76) | 3.07 (1.62) | 7.35 (3.82) |
| Smoking cessation | 0.00 (0.00) | 0.00 (0.00) | 0.74 (0.06) | 0.71 (0.03) |
| **Overall total for six months** | **576.96 (20.65)** | **464.16 (11.81)** | **292.07 (1.84)** | **255.28 (3.88)** |
| Adjusted incremental costs | 108.46 (95%CI 69.69 to 157.88) | | 37.06 (95% CI 28.12 to 43.85) | |
| **QALYs** | **0.408 (0.002)** | **0.408 (0.003)** | **0.387 (0.002)** | **0.392 (0.002)** |
| Adjusted incremental QALYs | 0.001 (95% CI -0.004 to 0.008) | | -0.003 (95% CI -0.006 to 0.000) | |
| ICER | 108,464 per QALY (uncertainty see Figure 3) | | Cytisine dominated by placebo (uncertainty see Figure 3) | |

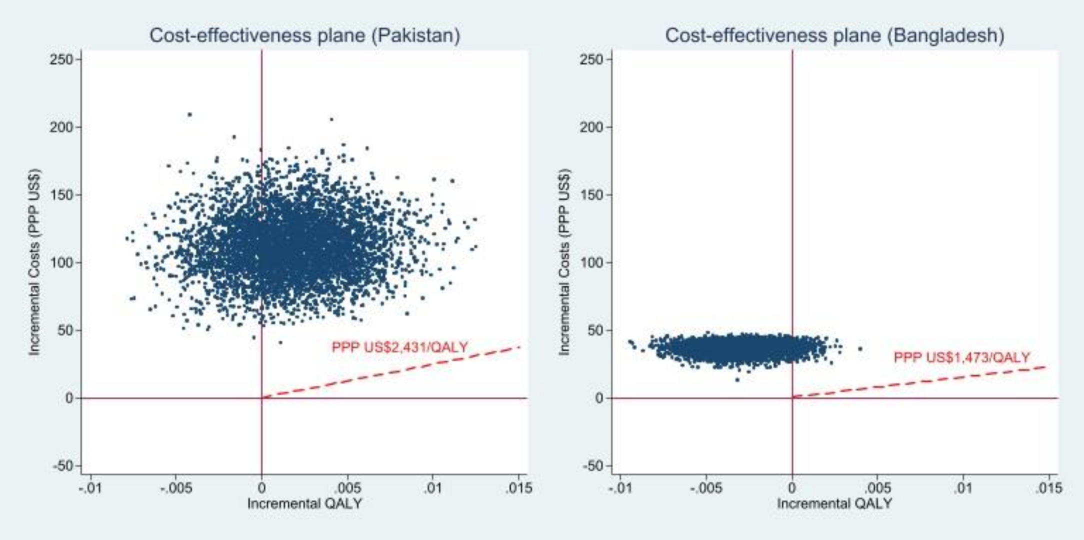


*Figure 3 Cost-effectiveness plane of cost-utility analysis results by country*

# References

1. Bangladesh Bureau of Statistics. Report on Labour Force Survey (LFS) 2016-17, 2018.
2. Pakistan Bureau of Statistics. Labour Force Survey 2017-18, 2018.
3. World Health Organization. Global Tuberculosis report 2018 - TB data: WHO, 2018.
4. Gomez GB, Dowdy DW, Bastos ML, et al. Cost and cost-effectiveness of tuberculosis treatment shortening: a model-based analysis. *BMC Infect Dis* 2016;16(1):726. doi: 10.1186/s12879-016-2064-3 [published Online First: 2016/12/03]
5. The World Bank. DataBank World Development Indicators, 2019.
6. International Monetary Fund. National Currency per U.S. Dollar, period average. Inernational Financial Statistics: International Monetary Fund (IMF), 2019.
7. World Health Organization. Tobacco control country profile Pakistan. Tobacco control country profiles, 2017.
8. World Health Organization. Tobacco control country profile Bangladesh. Tobacco control country profiles, 2017.
9. National Institute for Health and Care Excellence. Putting NICE guidance into practice - Resource impact report: Stop smoking interventions and services (NG92), 2018.
10. Curtis LA, Burns A. Unit Costs of Health and Social Care 2018. Kent: University of Kent, 2018.
11. Economic Analysis and Evaluation Team. Country-specific inpatient and outpatient estimates in 2010 currency: World Health Organisation, 2010.
12. International Monetary Fund. Prices, Consumer Price Index, All items, Index. International Financial Statistics (IFS): International Monetary Fund (IMF), 2019.
13. Woods B, Revill P, Sculpher M, et al. Country-Level Cost-Effectiveness Thresholds: Initial Estimates and the Need for Further Research. *Value Health* 2016;19(8):929-35. doi: 10.1016/j.jval.2016.02.017 [published Online First: 2016/12/19]
14. International Monetary Fund (IMF). Prices, Consumer Price Index, All items, Index. International Financial Statistics (IFS): International Monetary Fund (IMF), 2019.
