# Supplementary material for: Cost-utility of cytisine for smoking cessation over and above behavioural support in people with newly diagnosed pulmonary tuberculosis: an economic evaluation of a multicentre randomised controlled trial
Source: BMJ Open. 2022 Aug 26;12(8):e049644. doi: 10.1136/bmjopen-2021-049644 (PMC9422837; doi:10.1136/bmjopen-2021-049644)
Supplement: Supplementary data [file bmjopen-2021-049644supp002.pdf]

TB & Tobacco

CASE REPORT FORM - Visit (DAY 0)

Site ID: ☐☐ **Trial Number:** ☐☐☐☐☐☐ **Date of Completion:** ☐☐ / ☐☐ / ☐☐☐☐
Day Month Year

Section V

ECONOMIC EVALUATION *(This section is about the patient's wider health care use in the past six months, unless stated as TB-specific, this is for any illness.)*

Please exclude care provided by the trial intervention in your answers to these questions.

All costs should be specified in local currency, please round all costs up to the nearest whole number.

Enter a number for each item, if none, enter "0" (zero).

Please use this information to guide you if the patient gives estimates-

For daily visit: One week= 7 days, one month= 30 days, three months= 90 days, six months= 180 days.
For weekly visit per month= 4 times.

(i.e "I visited a centre daily for 6 months" would be 180 times)

1. Have you visited a TB clinic in the past six months?
(please exclude current visit and include visits to diagnostic centres if separate from clinics)

☐ Yes ☐ No (go to Q2)

If 'Yes'

a. How many times have you visited a public/voluntary TB clinic? ☐☐☐

b. How many times have you visited a private TB clinic? ☐☐☐

In **total** how much did you pay in the past six months (for consultation, diagnostics, procedures, drugs)? *(in local currency)* ☐☐☐☐☐☐

c. How much did you usually pay for your own travel per visit? *(in local currency)* ☐☐☐☐☐☐

d. On how many of these visits were you accompanied by a friend/relative? ☐☐☐

e. How much time in **total** did it usually take per visit *(travel, waiting, procedure)*? ☐☐ hours ☐☐ minutes

Li J, et al. BMJ Open 2022; 12:e049644. doi: 10.1136/bmjopen-2021-049644

TB & Tobacco Trial Number: ☐☐☐☐☐☐

2. Have you visited a doctor in the past six months (*for any illness and exclude TB clinic visits recorded in Q1)*?

☐ Yes ☐ No (go to Q3)

If 'Yes'

a. How many times have you visited a public/voluntary doctor? ☐☐☐

In the past six months, in **total** how much did you pay for public/voluntary visits *(for consultation, diagnostics, procedure, drugs)*? *(in local currency)* ☐☐☐☐☐☐

b. How many times have you visited a private doctor? ☐☐☐

In the past six months, in **total** how much did you pay for private visits *(for consultation, diagnostics, procedure, drugs)*? *(in local currency)* ☐☐☐☐☐☐

c. How much time did you usually spend with the doctor per visit? ☐☐ hours ☐☐ minutes

d. How much did you usually pay for your own travel per visit? *(in local currency)* ☐☐☐☐☐☐

e. On how many of these visits were you accompanied by a friend/relative etc.? ☐☐☐

f. How much time in **total** did it usually take per visit *(travel, waiting, procedure)*? ☐☐ hours ☐☐ minutes

3. Have you been admitted to hospital in the past six months (*for any illness*)?

☐ Yes ☐ No (go to Q4)

If 'Yes'

a. How many nights were you in a public/voluntary hospital? ☐☐☐

In **total** how much did you pay in the past six months at public/voluntary hospitals *(for consultation, diagnostics, procedures, drugs, overnight stay)*? *(in local currency)* ☐☐☐☐☐☐

b. How many nights were you in a private hospital? ☐☐☐

In **total** how much did you pay in the past six months at private hospitals *(for consultation, diagnostics, procedures, drugs, overnight stay)*? *(in local currency)* ☐☐☐☐☐☐

c. How much did you usually pay for your own travel per visit? *(in local currency)* ☐☐☐☐☐☐

Li J, *et al. BMJ Open* 2022; 12:e049644. doi: 10.1136/bmjopen-2021-049644

TB & Tobacco

Trial Number: ☐☐☐☐☐☐

4. Have you received any help to stop smoking in the past six months? *(please exclude the behavioural support session immediately before joining the trial, the session provided by the trial and any medication provided by the trial)*

☐ Yes (go to Q5) ☐ No (go to Q6)

5. How many times in the past six months have you *(this question is only about smoking cessation)*: Enter a number for each item, if none enter '0' (zero).

	Number of times	Amount spent out of pocket *(in local currency)*
Had help or advice about smoking from a public/government clinic/hospital?	☐☐☐	☐☐☐☐☐
Had help or advice about smoking from a private clinic/hospital?	☐☐☐	☐☐☐☐☐
Attended a group or single counselling session on smoking at a public/voluntary clinic?	☐☐☐	☐☐☐☐☐
Attended a group or single counselling session on smoking at a private clinic/hospital?	☐☐☐	☐☐☐☐☐
Been given a prescription for nicotine patches?	☐☐☐	☐☐☐☐☐
Been given a prescription for an alternative form of NRT? (such as gum, lozenge, inhaler, etc)	☐☐☐	☐☐☐☐☐
Bought a refill for an electronic cigarette?	☐☐☐	☐☐☐☐☐
Been given a prescription for Zyban (Bupropion)?	☐☐☐	☐☐☐☐☐
Been given a prescription for Champix (Varenicline)?	☐☐☐	☐☐☐☐☐
Received any traditional medicine? (Hakeem, Homeopathic, Unani etc.)	☐☐☐	☐☐☐☐☐
Other: please describe:	☐☐☐	☐☐☐☐☐

Li J, *et al. BMJ Open* 2022; 12:e049644. doi: 10.1136/bmjopen-2021-049644

TB & Tobacco

Trial Number: ☐☐☐☐☐☐

6. Have you received any medications for TB in the past six months?

☐ Yes (go to Q7) ☐ No (go to Q8)

7. Please detail below the medications for TB related illness in the past six months?
(Use the colour of the packets to indicate each medication)

(If patient answers not in days: one week= 7 days, one month= 30 days, three months= 90 days, six months= 180 days etc.)

**Anti-TB medication**	**Number of tablets per day**	**Duration receiving tablets (days)**
Fixed-dose combination (4 drugs) *(R-150mg/H-75mg/E-275mg/P-400mg)*		
Fixed-dose combination (2 drugs) *(R-150mg/H-75mg)*		

8. Do you have a paid job? *(include self-employed and employed) (Please tick one only)*

☐ I have a full time job (go to Q9)

☐ I have a part time job (go to Q9)

☐ I do not have a job (go to Q10)

9. Have you been off work sick in the past six months *(for any illness)*?

☐ Yes

☐ No (go to Q10)

If 'Yes' how many days were you off work sick in the last six months? ☐☐☐

10. Usually how much did you spend **per day** on tobacco over the past six months?
(In local currency) ☐☐☐☐☐☐

Li J, *et al. BMJ Open* 2022; 12:e049644. doi: 10.1136/bmjopen-2021-049644

TB & Tobacco

Trial Number: ☐☐☐☐☐☐

EURO QOL

This section asks about your health in general.
Under each heading, please tick the ONE box that best describes your health TODAY.

MOBILITY

I have no problems in walking about ☐

I have slight problems in walking about ☐

I have moderate problems in walking about ☐

I have severe problems in walking about ☐

I am unable to walk about ☐

SELF-CARE

I have no problems washing or dressing myself ☐

I have slight problems washing or dressing myself ☐

I have moderate problems washing or dressing myself ☐

I have severe problems washing or dressing myself ☐

I am unable to wash or dress myself ☐

USUAL ACTIVITIES *(e.g. work, study, housework, family or leisure activities)*

I have no problems doing my usual activities ☐

I have slight problems doing my usual activities ☐

I have moderate problems doing my usual activities ☐

I have severe problems doing my usual activities ☐

I am unable to do my usual activities ☐

PAIN/DISCOMFORT

I have no pain or discomfort ☐

I have slight pain or discomfort ☐

I have moderate pain or discomfort ☐

I have severe pain or discomfort ☐

I have extreme pain or discomfort ☐

ANXIETY/DEPRESSION

I am not anxious or depressed ☐

I am slightly anxious or depressed ☐

I am moderately anxious or depressed ☐

I am severely anxious or depressed ☐

I am extremely anxious or depressed ☐

UK (English) © 2009 EuroQol Group. EQ-5D™ is a trade mark of the EuroQol Group

TB & Tobacco

Trial Number: ☐☐☐☐☐☐

The best health you can imagine

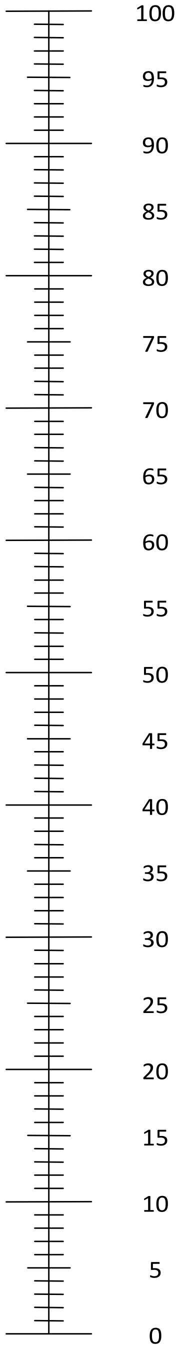

- We would like to know how good or bad your health is TODAY.
- The scale is numbered from 0 to 100.
- 100 means the best health you can imagine.

  0 means the worst health you can imagine.
- Mark an X on the scale to indicate how your health is TODAY.
- Now, please write the number you marked on the scale in the box below.

YOUR HEALTH TODAY = ☐☐☐

The worst health you can imagine

UK (English) © 2009 EuroQol Group. EQ-5D™ is a trade mark of the EuroQol Group

Please dispense medication for 1 week. Instruct the participant to come back for follow up coinciding with their quit date and also to bring the blister packets and the 'dosing schedule card'.

Thank you for your time!

Send data
