# Supplementary material for: Cost-utility of cytisine for smoking cessation over and above behavioural support in people with newly diagnosed pulmonary tuberculosis: an economic evaluation of a multicentre randomised controlled trial
Source: BMJ Open. 2022 Aug 26;12(8):e049644. doi: 10.1136/bmjopen-2021-049644 (PMC9422837; doi:10.1136/bmjopen-2021-049644)
Supplement: Supplementary data [file bmjopen-2021-049644supp003.pdf]

# Supplementary tables

*Table S1 Average hourly wage by occupation in Pakistan and Bangladesh*

| | Average hourly wage (PPP US$) [1-3] | | | | | | |
|---|---|---|---|---|---|---|---|
| | Bangladesh | | | | Pakistan | | |
| Occupation | Male | Female | Total | | Male | Female | Total |
| Managers | 5.68 | 5.13 | 5.62 | | 9.67 | 0.84 | 9.57 |
| Professionals | 4.25 | 3.93 | 4.13 | | 6.06 | 3.84 | 5.30 |
| Technicians and Associate Professionals | 3.35 | 3.21 | 3.32 | | 4.69 | 3.27 | 4.50 |
| Clerical support workers | 2.56 | 2.33 | 2.53 | | 4.69 | 3.16 | 4.66 |
| Service and Sales workers | 1.88 | 1.76 | 1.86 | | 2.85 | 2.37 | 2.83 |
| Skilled Agricultural, forestry and fisheries | 1.50 | 1.24 | 1.46 | | 3.03 | 0.98 | 2.96 |
| Craft and Related Trades workers | 1.69 | 1.55 | 1.65 | | 3.00 | 0.89 | 2.67 |
| Plant and Machine Operators, and Assembler | 1.91 | 1.77 | 1.89 | | 2.96 | 1.95 | 2.95 |
| Elementary Occupations | 1.38 | 1.15 | 1.32 | | 2.39 | 1.11 | 2.15 |
| Overall | 2.14 | 1.93 | 2.09 | | 3.35 | 2.00 | 3.15 |

*Table S2 Number and proportion of missing values of variables by arm*

| | Cytisine (n=1239) | | Placebo (n=1233) | |
|---|---|---|---|---|
| Variables | Number of missing values | Proportion of missing values | Number of missing values | Proportion of missing values |
| Cost of behavioural support | 6 | 0% | 7 | 1% |
| Cost of TB treatment | 102 | 8% | 103 | 8% |
| Cost of doctor visit at d0 | 0 | 0% | 1 | 0% |
| Cost of doctor visit at m6 | 61 | 5% | 67 | 5% |
| Cost of doctor visit at m12 | 89 | 7% | 76 | 6% |
| Cost of hospital stay at d0 | 2 | 0% | 2 | 0% |
| Cost of hospital stay at m6 | 62 | 5% | 67 | 5% |
| Cost of hospital stay at m12 | 89 | 7% | 76 | 6% |
| Cost of smoking cessation at d0 | 0 | 0% | 0 | 0% |
| Cost of smoking cessation at m6 | 63 | 5% | 69 | 6% |
| Cost of smoking cessation at m12 | 103 | 8% | 89 | 7% |
| OOP on TB treatment at d0 | 1 | 0% | 0 | 0% |
| OOP on TB treatment at m6 | 63 | 5% | 69 | 6% |
| OOP on TB treatment at m12 | 89 | 7% | 77 | 6% |
| OOP on smoking cessation at d0 | 3 | 0% | 3 | 0% |
| OOP on smoking cessation at m6 | 146 | 12% | 153 | 12% |
| OOP on smoking cessation at m12 | 127 | 10% | 118 | 10% |
| OOP on doctor visit at d0 | 6 | 0% | 6 | 0% |
| OOP on doctor visit at m6 | 64 | 5% | 75 | 6% |
| OOP on doctor visit at m12 | 89 | 7% | 76 | 6% |
| OOP on hospital stay at d0 | 2 | 0% | 2 | 0% |
| OOP on hospital stay at m6 | 64 | 5% | 69 | 6% |
| OOP on hospital stay at m12 | 89 | 7% | 76 | 6% |
| OOP on tobacco products d0 | 10 | 1% | 9 | 1% |
| OOP on tobacco products m6 | 60 | 5% | 67 | 5% |
| OOP on tobacco products m12 | 89 | 7% | 76 | 6% |
| Productivity loss of company for TB treatment at d0 | 7 | 1% | 5 | 0% |
| Productivity loss of company for TB treatment at m6 | 103 | 8% | 106 | 9% |
| Productivity loss of company for TB treatment at m12 | 92 | 7% | 81 | 7% |
| Productivity loss of company for doctor at d0 | 36 | 3% | 37 | 3% |
| Productivity loss of company for doctor at m6 | 111 | 9% | 117 | 9% |
| Productivity loss of company for doctor at m12 | 94 | 8% | 82 | 7% |
| Productivity loss of sick leave at d0 | 9 | 1% | 6 | 0% |
| Productivity loss of sick leave at m6 | 44 | 4% | 62 | 5% |

| | Cytisine (n=1239) | | Placebo (n=1233) | |
|---|---|---|---|---|
| Variables | Number of missing values | Proportion of missing values | Number of missing values | Proportion of missing values |
| Productivity loss of sick leave at m12 | 75 | 6% | 73 | 6% |
| EQ-5D-5L at d0 | | | | |
| 1 Mobility | 0 | 0% | 0 | 0% |
| 2 Self-care | 0 | 0% | 2 | 0% |
| 3 Usual activities | 2 | 0% | 0 | 0% |
| 4 Pain and discomfort | 1 | 0% | 1 | 0% |
| 5 Anxiety or depression | 2 | 0% | 1 | 0% |
| EQ-5D-5L at m6 | | | | |
| 1 Mobility | 60 | 5% | 67 | 5% |
| 2 Self-care | 60 | 5% | 67 | 5% |
| 3 Usual activities | 60 | 5% | 68 | 6% |
| 4 Pain and discomfort | 60 | 5% | 68 | 6% |
| 5 Anxiety or depression | 60 | 5% | 68 | 6% |
| EQ-5D-5L at m12 | | | | |
| 1 Mobility | 89 | 7% | 76 | 6% |
| 2 Self-care | 89 | 7% | 78 | 6% |
| 3 Usual activities | 89 | 7% | 78 | 6% |
| 4 Pain and discomfort | 90 | 7% | 78 | 6% |
| 5 Anxiety or depression | 94 | 8% | 78 | 6% |
| VAS at d0 | 0 | 0% | 0 | 0% |
| VAS at m6 | 60 | 5% | 68 | 6% |
| VAS at m12 | 89 | 7% | 77 | 6% |
| TB score at d0 | 0 | 0% | 0 | 0% |
| TB score at m6 | 60 | 5% | 66 | 5% |

*Table S3 Logistic regression for missingness of costs, OOPs, productivity loss and outcomes on arm and baseline covariates*

| Missing on: | Allocation | Age | Country |
|---|---|---|---|
| Cost of TB treatment | 1.02 (0.76-1.35) | 1.02 (1.01-1.03) | **0.26 (0.19-0.36)*** |
| Cost of doctor visit at m6 | 1.07 (0.75-1.53) | 1.00 (0.99-1.01) | **0.15 (0.10-0.24)*** |
| Cost of doctor visit at m12 | 0.83 (0.61-1.14) | 1.00 (0.99-1.01) | **0.17 (0.12-0.25)*** |
| Cost of hospital stay at m6 | 1.05 (0.74-1.50) | 1.00 (0.99-1.01) | **0.16 (0.11-0.24)*** |
| Cost of hospital stay at m12 | 0.83 (0.61-1.14) | 1.00 (0.99-1.01) | **0.17 (0.12-0.25)*** |
| Cost of smoking cessation at m6 | 1.07 (0.76-1.52) | 1.00 (0.99-1.01) | **0.17 (0.11-0.26)*** |
| Cost of smoking cessation at m12 | 0.84 (0.63-1.13) | 1.00 (0.99-1.01) | **0.28 (0.21-0.39)*** |
| OOP on TB treatment at m6 | 1.07 (0.76-1.52) | 1.00 (0.99-1.01) | **0.16 (0.10-0.24)*** |
| OOP on TB treatment at m12 | 0.84 (0.62-1.15) | 1.00 (0.99-1.01) | **0.17 (0.12-0.25)*** |
| OOP on smoking cessation at m6 | 1.04 (0.82-1.33) | **0.99 (0.98-1.00)*** | 1.14 (0.89-1.47) |
| OOP on smoking cessation at m12 | 0.91 (0.70-1.18) | 0.99 (0.98-1.00) | **0.52 (0.40-0.68)*** |
| OOP on doctor visit at m6 | 1.15 (0.82-1.62) | 1.00 (0.99-1.01) | **0.16 (0.10-0.24)*** |
| OOP on doctor visit at m12 | 0.83 (0.61-1.14) | 1.00 (0.99-1.01) | **0.17 (0.12-0.25)*** |
| OOP on hospital stay at m6 | 1.05 (0.74-1.49) | 1.00(0.99-1.01) | **0.16 (0.11-0.24)*** |
| OOP on hospital stay at m12 | 0.83 (0.61-1.14) | 1.00 (0.99-1.01) | **0.17 (0.12-0.25)*** |
| OOP on tobacco products m6 | 1.09 (0.76-1.55) | 1.00 (0.99-1.01) | **0.16 (0.10-0.24)*** |
| OOP on tobacco products m12 | 0.83 (0.61-1.14) | 1.00 (0.99-1.01) | **0.17 (0.12-0.25)*** |
| Productivity loss of company for TB treatment at m6 | 1.01 (0.77-1.35) | 0.99 (0.98-1.00) | **0.55 (0.41-0.73)*** |
| Productivity loss of company for TB treatment at m12 | 0.86 (0.63-1.16) | 1.00 (0.99-1.01) | **0.18 (0.13-0.26)*** |
| Productivity loss of company for doctor at m6 | 1.04 (0.80-1.37) | 0.99 (0.98-1.00) | **0.52 (0.40-0.69)*** |
| Productivity loss of company for doctor at m12 | 0.85 (0.63-1.15) | 1.00 (0.99-1.01) | **0.18 (0.12-0.25)*** |
| Productivity loss of sick leave at m6 | 1.40 (0.95-2.08) | 0.99 (0.97-1.00) | **0.22 (0.14-0.33)*** |
| Productivity loss of sick leave at m12 | 0.95 (0.68-1.32) | 0.99 (0.98-1.00) | **0.27 (0.19-0.38)*** |
| EQ-5D-5L at m6 | 1.09 (0.76-1.55) | 1.00 (0.99-1.01) | **0.16 (0.10-0.24)*** |
| EQ-5D-5L at m12 | 0.83 (0.61-1.14) | 1.00 (0.99-1.01) | **0.17 (0.12-0.25)*** |
| TB score at m6 | 1.02 (0.72-1.44) | 1.00 (0.99-1.01) | **0.18 (0.12-0.26)*** |

***P<0.05**

## References

1. Bangladesh Bureau of Statistics. Report on Labour Force Survey (LFS) 2016-17, 2018.
2. Pakistan Bureau of Statistics. Labour Force Survey 2017-18, 2018.
3. The World Bank. DataBank World Development Indicators, 2019.
