# Supplementary material for: Cost-utility of cytisine for smoking cessation over and above behavioural support in people with newly diagnosed pulmonary tuberculosis: an economic evaluation of a multicentre randomised controlled trial
Source: BMJ Open. 2022 Aug 26;12(8):e049644. doi: 10.1136/bmjopen-2021-049644 (PMC9422837; doi:10.1136/bmjopen-2021-049644)
Supplement: Author's manuscript [file bmjopen-2021-049644.draft_revisions.pdf]

BMJ Open is committed to open peer review. As part of this commitment we make the peer review history of every article we publish publicly available.

When an article is published we post the peer reviewers' comments and the authors' responses online. We also post the versions of the paper that were used during peer review. These are the versions that the peer review comments apply to.

The versions of the paper that follow are the versions that were submitted during the peer review process. They are not the versions of record or the final published versions. They should not be cited or distributed as the published version of this manuscript.

BMJ Open is an open access journal and the full, final, typeset and author-corrected version of record of the manuscript is available on our site with no access controls, subscription charges or pay-per-view fees (http://bmjopen.bmj.com).

If you have any questions on BMJ Open's open peer review process please email
info.bmjopen@bmj.com

BMJ Open

Cost-effectiveness of cytisine for smoking cessation over and above behavioural support in people with newly diagnosed pulmonary tuberculosis: An economic evaluation of a multi-centre randomised controlled trial

Journal:	*BMJ Open*
Manuscript ID	bmjopen-2021-049644
Article Type:	Original research
Date Submitted by the Author:	28-Jan-2021
Complete List of Authors:	Li, Jinshuo; University of York Department of Health Sciences<br>Parrott, Steve; University of York Department of Health Sciences<br>Keding, Ada; University of York Department of Health Sciences<br>Dogar, Omara; University of York Department of Health Sciences; The University of Edinburgh Usher Institute of Population Health Sciences and Informatics<br>Gabe, Rhian; Queen Mary University of London, Centre for Cancer Prevention, Wolfson Institute of Preventive Medicine<br>Marshall, Anna-Marie; University of York Department of Health Sciences<br>Huque, Rumana; University of Dhaka Department of Economics; ARK Foundation<br>Barua, Deepa; ARK Foundation<br>Fatima, Razia; Global Fund Grant, TB/HIV and Malaria Common Management Unit<br>Khan, Amina; The Initiative<br>Zahid, Raana; The Initiative<br>Mansoor, Sonia; Rawalpindi Medical University, Institute of Psychiatry<br>Kotz, Daniel; Heinrich-Heine-Universitat Dusseldorf, Institute of General Practice, Addiction Research and Clinical Epidemiology Unit; The University of Edinburgh Usher Institute of Population Health Sciences and Informatics<br>Boeckmann, Melanie; Heinrich-Heine-Universitat Dusseldorf, Institute of General Practice, Addiction Research and Clinical Epidemiology Unit; Bielefeld University, School of Public Health, Department of Environment and Health<br>Elsey, Helen ; University of York Department of Health Sciences<br>Kralikova, Eva; Charles University, Institute of Hygiene and Epidemiology; General University Hospital in Prague, 3rd Medical Department<br>Readshaw, Anne; University of York Department of Health Sciences<br>Sheikh, Aziz; The University of Edinburgh Usher Institute of Population Health Sciences and Informatics<br>Siddiqi, Kamran; University of York Department of Health Sciences; Hull York Medical School
Keywords:	Tuberculosis < INFECTIOUS DISEASES, HEALTH ECONOMICS, Clinical trials < THERAPEUTICS, International health services < HEALTH SERVICES ADMINISTRATION & MANAGEMENT

SCHOLARONE™
Manuscripts

I, the Submitting Author has the right to grant and does grant on behalf of all authors of the Work (as defined in the below author licence), an exclusive licence and/or a non-exclusive licence for contributions from authors who are: i) UK Crown employees; ii) where BMJ has agreed a CC-BY licence shall apply, and/or iii) in accordance with the terms applicable for US Federal Government officers or employees acting as part of their official duties; on a worldwide, perpetual, irrevocable, royalty-free basis to BMJ Publishing Group Ltd ("BMJ") its licensees and where the relevant Journal is co-owned by BMJ to the co-owners of the Journal, to publish the Work in this journal and any other BMJ products and to exploit all rights, as set out in our licence.

The Submitting Author accepts and understands that any supply made under these terms is made by BMJ to the Submitting Author unless you are acting as an employee on behalf of your employer or a postgraduate student of an affiliated institution which is paying any applicable article publishing charge ("APC") for Open Access articles. Where the Submitting Author wishes to make the Work available on an Open Access basis (and intends to pay the relevant APC), the terms of reuse of such Open Access shall be governed by a Creative Commons licence – details of these licences and which Creative Commons licence will apply to this Work are set out in our licence referred to above.

Other than as permitted in any relevant BMJ Author's Self Archiving Policies, I confirm this Work has not been accepted for publication elsewhere, is not being considered for publication elsewhere and does not duplicate material already published. I confirm all authors consent to publication of this Work and authorise the granting of this licence.

For peer review only

Cost-effectiveness of cytisine for smoking cessation over and above behavioural support in people with newly diagnosed pulmonary tuberculosis: An economic evaluation of a multi-centre randomised controlled trial

Jinshuo Li, M.Phil[1], Steve Parrott, MSc[1], Ada Keding, MSc[1], Omara Dogar, PhD[1,2], Rhian Gabe, PhD[3], Anna-Marie Marshall, PhD[1], Rumana Huque, PhD[4, 5], Deepa Barua, MPH[4], Razia Fatima, PhD[6], Amina Khan, MPH[7], Raana Zahid, MPH[7], Sonia Mansoor, MSc[8], Daniel Kotz, PhD[2,9,10], Melanie Boeckmann, DrPH[1,9,11], Helen Elsey PhD, FFPH[1], Eva Kralikova, PhD, MD[12, 13], Anne Readshaw, PhD[1], Aziz Sheikh, MD[2] & Kamran Siddiqi, PhD[1,14], *on behalf of the TB & Tobacco Consortium*

Authors' affiliations

[1]University of York, Department of Health Sciences, Faculty of Sciences, YO10 5DD, York, UK

[2]Usher Institute, The University of Edinburgh, EH8 9AG, Edinburgh, UK

[3] Centre for Cancer Prevention, Wolfson Institute of Preventive Medicine, Queen Mary University of London, United Kingdom

[4]ARK Foundation, Dhaka, Bangladesh

[5]Department of Economics, University of Dhaka, Bangladesh

[6]TB/HIV and Malaria Common Management Unit, Global Fund Grant, Islamabad, Pakistan

[7]The Initiative, Islamabad, Pakistan

[8] Institute of Psychiatry (IOP) Rawalpindi Medical University, Pakistan

[9]Institute of General Practice, Addiction Research and Clinical Epidemiology Unit, Medical Faculty of the Heinrich-Heine-University, Düsseldorf, Germany

[10]Department of Family Medicine, CAPHRI School for Public Health and Primary Care, Maastricht University, The Netherlands

[11]Bielefeld University, School of Public Health, Department of Environment and Health, Bielefeld, Germany

[12]3rd Medical Department, First Faculty of Medicine, Charles University, and the General University Hospital in Prague, Czech Republic

[13]Institute of Hygiene and Epidemiology, First Faculty of Medicine, Charles University, and the General University Hospital in Prague, Czech Republic

[14]Hull York Medical School, University of York, YO10 5DD, York, UK

For peer review only - http://bmjopen.bmj.com/site/about/guidelines.xhtml

Corresponding author: Jinshuo Li, e-mail: jinshuo.li@york.ac.uk, tel: +44 1904 32 1358, Area 4 ARRC building, Department of Health Sciences, University of York, Heslington, YO10 4DU, York, United Kingdom

Word count: 3880

For peer review only

For peer review only - http://bmjopen.bmj.com/site/about/guidelines.xhtml

Abstract

Objectives

To assess the cost-effectiveness of cytisine over and above brief behavioural support (BS) for smoking cessation among newly diagnosed pulmonary tuberculosis (TB) patients in low- and middle-income countries.

Design

An incremental cost-effectiveness analysis was undertaken alongside a 12-month double-blind two-arm individually randomised controlled trial from a public/voluntary health services perspective, with the primary endpoint at six months post-randomisation.

Setting

Thirty-two sites across two countries: 17 sub-district hospitals in Bangladesh and 15 secondary care hospitals in Pakistan.

Participants

Adults (aged ≥18 years in Bangladesh and ≥15 years in Pakistan) with pulmonary TB diagnosed within the last four weeks, who smoked tobacco daily (n=2472).

Interventions

Two brief smoking cessation BS sessions with a trained TB health worker were offered to all participants. Participants in the intervention arm (n=1239) were given cytisine (25-day course) while those in the control arm (n=1233) were given placebo.

Primary and secondary outcome measures

Costs of cytisine and BS sessions were estimated based on research team records. TB treatment costs were estimated based on TB registry records. Additional smoking cessation and healthcare costs and EQ-5D-5L data were collected at baseline, six- and 12-month follow-ups. Costs were presented in Purchasing Power Parity (PPP) adjusted US dollars (US$). Quality-adjusted life years (QALYs) were derived from the EQ-5D-5L. Incremental total costs and incremental QALYs were estimated using regressions adjusting for respective baseline values and other baseline covariates. Uncertainty was assessed using bootstrapping.

Results

Mean total costs were PPP US$57.74 (95% CI 49.40 – 83.36) higher in the cytisine arm than in the placebo arm while the mean QALYs were -0.001 (95% CI -0.004 – 0.002) lower over six months, hence the cytisine arm was dominated by the placebo arm.

Conclusions

Cytisine *plus* BS for smoking cessation among TB patients was not cost-effective in comparison with placebo *plus* BS.

Clinical trial registration: International Standard Randomized Clinical Trial Number, ISRCTN43811467

For peer review only - http://bmjopen.bmj.com/site/about/guidelines.xhtml

Strength and limitations of this study

- Large sample size and high follow-up rate ensures robustness of the conclusion
- Comprehensive patient-level data collection provides possibilities of further exploration or updating of the analyses
- Trial across two countries posed challenges to value both costs and quality-adjusted life years comparably
- Lack of up-to-date data sources of unit costs of healthcare services may affect the accuracy of the costs estimation
- Eagerness of local staff participating the trial may affect the generalisability of the intervention delivery

For peer review only

INTRODUCTION

Evidence suggests that smoking is associated with unfavourable tuberculosis (TB) treatment outcomes.[1] While Bangladesh and Pakistan were among the eight high-TB burden countries in 2018,[2] and tobacco use is also common in both countries,[3, 4] support for smoking cessation for TB patients is mostly absent.[5]

TB treatment, lasting six months or longer, offers an opportunity for regular support for quitting smoking, if integrated properly. Newly diagnosed TB patients who smoke might be more receptive to advice to quit than those without TB.[6] Aware of the limited time available in routine TB appointments in many low- and middle-income countries (LMICs), we developed a short optimised and integrated behavioural support (BS) intervention for smoking cessation for TB patients. In terms of cessation aids, cytisine has been recommended in the general population in LMICs due to its relative low cost.[7] However, whether it is effective and cost-effective in TB patients who smoke had not been studied.

We conducted a 12-month, two-arm, parallel, double blind, placebo-controlled, multicentre, individually randomised trial in Bangladesh and Pakistan to compare cytisine plus BS for smoking cessation (cytisine arm: n=1239) with placebo plus BS (placebo arm: n=1233) among pulmonary TB patients who smoke daily.[8] Biochemically-verified continuous abstinence at 6 months (primary endpoint) was 32.4% (401/1239) in the cytisine arm and 29.7% (366/1233) in the placebo arm (RR=1.09, 95% CI 0.97-1.23) and at 12 months, it was 25% (309/1239) and 22% (275/1233), respectively (RR=1.22, 95% CI 0.95-5.98).[9] This article reports the analyses to: 1) evaluate the cost-effectiveness, from a public or voluntary sector perspective, of adding cytisine to BS for smoking cessation in TB patients who smoke; and 2) assess the financial burden in relation to tobacco use, healthcare and productivity loss of participants and their families from a societal perspective.

METHODS

Design

An incremental cost-utility analysis was conducted alongside the randomised controlled trial (RCT) described above and elsewhere.[8, 9] The scheduled follow-ups were at 6 and 12 months post-randomisation, with 6 months as the primary endpoint. Neither participants nor TB health workers were aware of participants' arm allocation. Allocation was not revealed to health economists until database lock. Detailed information please see study protocol attached as supplementary file.

Ethical approval was granted by the Health Sciences Research Governance Committee (HSRGC) at the University of York, UK (HSRGC/2016/144/B), the National Bioethics Committee, Pakistan Medical Research Council (no. 4–87/16/NBC-200 Part-B/RDC/4197) and the National Research Ethics Committee, Bangladesh Medical Research Council (BMRC/NREC/2016–2019/1475).

Participants

Adults (aged ≥18 in Bangladesh and ≥15 in Pakistan) with pulmonary TB diagnosed within the last four weeks who smoked tobacco on a daily basis and were interested in quitting, were eligible.[8] We excluded those who were receiving retreatment for TB, diagnosed with multi-drug resistance, miliary or extra-pulmonary TB, receiving Streptomycin and/or Para Amino Salicylic Acid, using any pharmacotherapy for tobacco dependence, pregnant or planning to become pregnant, lactating, or suffering from schizophrenia or known to be diagnosed with epilepsy. Those who had myocardial

For peer review only - http://bmjopen.bmj.com/site/about/guidelines.xhtml

infarction, stroke, or an attack of severe angina within the previous two weeks, uncontrolled high blood pressure despite being on medication, or severe renal impairment (requiring dialysis), were also excluded.

Between June 2017 and April 2018, 1527 participants from 17 sub-district hospitals in Bangladesh and 945 participants from 15 secondary care hospitals in Pakistan were randomised to the cytisine arm (n=1239) and the placebo arm (n=1233). The mean age was 42.5 (SD 14.3) years in the cytisine arm and 42.4 (SD 14.2) years in the placebo arm. Males made up 99% of each arm (1227 in the cytisine arm, 1221 in the placebo arm). By 6 months follow-up, 70 participants died (36 in the cytisine arm and 34 in the placebo arm). A further 21 participants died after 6 months (13 in the cytisine arm and eight in the placebo arm).

Intervention and comparator

Each participant was given a leaflet with information on tobacco use and its interactions with TB. Trained TB health workers offered brief BS for smoking cessation to both arms. It was designed to be delivered in two face-to-face sessions on days 0 (10 minutes) and 5 (5 minutes).

The standard regimen for cytisine (Desmoxan, Aflofarm) was a 25-day course with 1.5mg hard capsules for oral administration, totalling 100 capsules.[8, 9] The quit date was set on day 5. Both arms were provided with 38 capsules on day 0 and another 62 capsules on day 5. While the cytisine arm received cytisine, the placebo arm was given placebo capsules with identical appearance.

Measures

All monetary outcomes were collected or valued in local currencies and inflated to their respective 2018 values [10] where necessary, and converted to purchasing power parity adjusted US dollars (PPP US$) using the World Bank exchange rate (1 PPPUS$ = 30.9 Bangladeshi Taka = 29.3 Pakistani Rupees).[11] PPP US$ accounts for the price and income difference between the two countries so that the monetary outcomes could be pooled together.

Costs

Intervention costs

Intervention costs included costs of training and delivery (Details see Supplementary file 1). TB health workers were trained in brief BS for smoking cessation in a two-day programme. The costs of training were estimated by the research team to be PPP US$14,183 in Bangladesh and PPP US$12,837 in Pakistan. Since all participants were scheduled to receive BS, the training cost was allocated to each participant evenly.

The uptake of BS was recorded on the Case Report Form (CRF) on day 0. Staff costs for BS were estimated by multiplying the duration by the hourly wage rate. The cost of BS for the first and second session was PPP US$0.52 and PPP US$0.26 in Bangladesh, and PPP US$0.75 and PPP US$0.38 in Pakistan. For those whose CRF showed not taking up BS, the cost of BS delivery was considered null. For those who accepted BS, the cost of the first session was applied and the cost of the second session was added provided they attended the follow-up on day 5. The smoking cessation information leaflet costed PPP US$0.16 in Bangladesh and PPP US$1.71 in Pakistan, per participant.

The manufacturer provided the distributor price as 72.63 Polish Złoty for 100 capsule pack (PPP US$42.27 in Bangladesh, PPP US$65.09 in Pakistan). By dispensing schedule, the medication dispensed on day 0 costed PPP US$16.05 in Bangladesh and PPP US$24.74 in Pakistan, and on day 5

For peer review only - http://bmjopen.bmj.com/site/about/guidelines.xhtml

it costed PPP US$26.21 and PPP US$40.34, respectively. The placebo capsules were assumed to incur no cost. All participants had at least the first dispense and those who missed follow-up on day 5 were assumed not to receive the second dispense.

Costs of TB treatment, additional smoking cessation help, and general healthcare services

Table 1 presents the unit costs of TB treatment by phase, additional smoking cessation services, and general healthcare services, estimated based on secondary sources and some assumptions and converted to PPP US$ 2018.[11-21] Detailed methods of estimation see Supplementary file 1. TB treatment progression was estimated according to the TB registry card. The quantities of services use were collected by self-report at baseline, 6- and 12-month follow-ups (See Supplementary file 2 for CRF).

[Insert Table 1 here]

Out-of-pocket payments (OOPs) and productivity loss

Participants reported any spending in monetary form related to TB treatment, smoking cessation products, and general healthcare services use, including travel, on CRFs at baseline, 6- and 12-month follow-ups.

CRFs also collected participants' time spent in TB clinic and doctor visits, including travel and waiting time, and if and how many times they were accompanied by a friend or relative. The productivity loss of a companion was estimated by multiplying the overall time spent by the companion by societal average hourly wage in the country.[19, 20] We assumed that all companions were employed. Participants' productivity loss was estimated based on their self-reported duration of sick leave from work. Participants' hourly wage were extracted from secondary sources based on their occupation category and gender,[19, 20] with those reported in open question re-classified according to the International Standard Classification of Occupations ISCO-08 (Supplementary file 3 Table S1).[22] Those who were unemployed, retired, students or home makers, were assumed to incur no productivity loss in the case of sick leave.

Quality-adjusted life years (QALYs)

The EQ-5D-5L developed by the EuroQol Group was used to measure health-related quality of life,[23] at baseline, 6- and 12-month follow-ups, as part of the CRFs. The EQ-5D-5L consists of a descriptive system of five domains (Mobility, Self-care, Usual activities, Pain/Discomfort and Anxiety/Depression), and a Visual Analogue Scale (VAS) valuing the overall health on the day. The VAS score ranges from 0 (death) to 100 (perfect health). Each domain of the descriptive system has five levels of capacity, ranging from having no problem to having severe problems. A complete descriptive system could be converted to a utility value using an appropriate tariff.

In the absence of country-specific valuation sets for Bangladesh and Pakistan, we used the valuation set of Zimbabwe based on crosswalk function to calculate utility,[24] as its Gross Domestic Product per capita in PPP US$ (2,381.22) was the closest to that of the two countries of interest (Bangladesh: 4,598.39; Pakistan: 5,714.03) at the time of the analysis.[25] QALYs were derived using the area under the curve approach.[26]

Analyses

All analyses were performed using STATA 16.0 SE.

For peer review only - http://bmjopen.bmj.com/site/about/guidelines.xhtml

For peer review only

Missing data

For the baseline covariates, missing values were imputed by the mean of the variable in the pooled sample in the same country. This was the information that was unrelated to the intervention and the randomisation functioned to balance the two arms.[27] The missing values in the follow-up variables were handled using multiple imputation method, following Rubin's rule and assuming missing at random (MAR),[28] unless it was due to death. Missing values due to death were replaced with zero or not applicable (n/a) depending on the nature of variable. An imputation model was developed to include all the variables necessary for the analysis and the number of imputations was set as approximately the highest percentage figure of missing data.[29] The imputation was performed by trial arms and on condition of being alive.

Primary analysis

The primary analysis was an incremental cost-utility analysis over six months post-randomisation from public or voluntary sector perspectives. It was undertaken on an intention-to-treat basis, including all randomised participants in the arms to which they were allocated.

Total costs at 6 months consisted of intervention costs, TB treatment costs, additional public/voluntary smoking cessation costs, and public/voluntary healthcare services costs in the six months post-randomisation. Mean total costs and mean QALYs were estimated for each arm and no discounting was applied for the six months period. Incremental mean total costs and incremental mean QALYs was estimated by a mixed effect generalised linear regression model, adjusting for their respective baseline values (total costs in the six months before randomisation for total costs; baseline EQ-5D-5L utility for QALYs), age, gender, country, with study site as random-effects. An incremental cost-effectiveness ratio (ICER) was calculated by dividing the incremental mean total costs by the incremental mean QALYs.

Since there are no official willingness-to-pay (WTP) thresholds in either Bangladesh or Pakistan, the estimated WTPs for Bangladesh and Pakistan based on income elasticity of value of health, inflated to 2018, were used to compare with the ICERs, if applicable.[30]

Because neither costs nor QALYs were normally distributed, we used a non-parametric bootstrap technique to assess the uncertainty, generating 5000 replicate samples. The results were used to construct 95% confidence intervals (CIs) of the incremental costs and QALYs. They were then plotted on a cost-effectiveness plane (CEP) to demonstrate the uncertainty surrounding the ICER. Cost-effectiveness acceptability curves (CEACs) were constructed from these bootstrapped replicates by converting ICER to net monetary benefit.[31]

Sensitivity analyses

We undertook a complete case analysis (CCA) on the participants who had complete outcome and covariates data to provide a comparison with the primary analysis based on imputed data. We examined the MAR assumption that supports the multiple imputation by undertaking sensitivity analyses based on missing not at random (MNAR) assumptions using a practical approximation to the pattern mixture model:[27] (1) imputed total costs were increased by 10%, 20% and 30%; (2) imputed QALYs were reduced by between 10%, 20%, and 30%. To assess the impact of choice of EQ-5D-5L tariff, we took the validated population valuation sets from countries in the southeast Asia area (i.e. Indonesia, Malaysia, Thailand) and the crosswalk functions of the UK and Thailand to calculate utility for comparison.[24, 32-34]

For peer review only - http://bmjopen.bmj.com/site/about/guidelines.xhtml

For peer review only

Secondary analyses

The first secondary analysis followed the methods of the primary analysis, extending time horizon to a 12-month period. No discounting was applied as this was not longer than one year. We summarised participants' OOPs in relation to TB treatment, smoking cessation, and healthcare services by arm, at both 6 and 12 months. Productivity losses of participants' sick leave and their companion to treatment, and money spent on any forms of tobacco were also summarised.

RESULTS

Missing data

The results of observed cases are presented in Supplementary file 1. The proportion of missing data at baseline was low (Supplementary file 3 Table S2). The greatest percentage of missing data level was 12% of participants' OOPs for smoking cessation at 6 months follow-up, followed by the same variable at 12 months (10%).

Although missing data did not differentiate between arms, most of the missingness of follow-up variables was significantly associated with country. The missingness of OOP for smoking cessation in months 1-6 was weakly associated with participants' age (Supplementary file 3 Table S3). Using a logistic regression for missingness of follow-up variables on their respective previously observed values (e.g. missingness of costs at six months on costs at baseline), most results were not statistically significant (p>0.05), with few exceptions. These results supported the MAR assumption. The imputation number was set to 15.

Primary analysis

The mean costs of smoking cessation and healthcare services in the six months before baseline were PPP US$10.36 (SE PPP US$1.74) in the cytisine arm and PPP US$8.52 (SE PPP US$1.41) in the placebo arm. The mean total costs over the six months post-randomisation were PPP US$401.52 (SE PPPUS$8.91) in the cytisine arm and PPP US$334.73 (SE PPP US$5.85) in the placebo arm (Table 2). Costs of additional smoking cessation were negligible in both arms. The mean costs of hospital stay in the cytisine arm were almost twice of those in the placebo arm. The incremental total costs were PPP US$57.74 (95% CI PPP US$49.40 to PPP US$83.36). The mean QALYs were 0.395 (SE 0.002) in the cytisine arm and 0.398 (SE 0.002) in the placebo arm. The incremental QALYs were -0.001 (95% CI -0.004 to 0.002). The majority (78.1%, 3905/5000) of the bootstrapped replicates fell in the north-west quadrant of CEP, indicating a more costly, but less effective intervention (Figure 1 left). The CEAC was not presented as it was a straight line at 0% probability of cost-effectiveness.

[Insert Table 2 here]

[Insert Figure 1 here]

Sensitivity analyses

The CCA was performed on 1122 participants in the cytisine arm and 1116 participants in the placebo arm. The results were similar to that of the primary analysis (Table 2 right). The overall majority (91%, 4550/5000) of the bootstrapped replicates fell in the north-west quadrant of CEP (Figure 1 right), indicating a more costly, but less effective intervention. This was consistent with the primary analysis.

For peer review only - http://bmjopen.bmj.com/site/about/guidelines.xhtml

Under scenario (1), when the imputed costs were increased by 10%, 20% and 30%, the incremental costs became PPP US$58.32, PPP US$58.91 and PPP US$59.51, respectively. Under scenario (2), when the imputed QALYs were reduced by 10%, 20% and 30%, the incremental QALYs were -0.001, -0.001 and -0.000, respectively. None differed far from the primary analysis results.

Using tariffs derived in different countries or with different approaches, the incremental QALYs between arms varied (Figure 2), but the level of difference was not prominent and the general pattern between arms remained the same.

[Insert Figure 2 here]

Secondary analyses

The addition of the costs in months 7-12 increased the mean total costs over 12 months to PPP US$408.31 (SE PPP US$10.03) in the cytisine arm and PPP US$341.83 (SE PPP US$6.50) in the placebo arm. The incremental costs were PPP US$56.72 (95% CI PPP US$46.58 to PPP US$86.00), similar to those over the six months post randomisation. By contrast, as the time horizon doubled, the QALYs became almost twice as high as over six-month period, which led to a larger difference in mean QALYs between arms. The mean QALYs were 0.808 (SE 0.004) in the cytisine arm and 0.814 (SE 0.004) in the placebo arm. The incremental QALYs were -0.004 (95% CI -0.013 to 0.005). The cytisine arm remained dominated by the placebo arm, with 77% (4007/5000) of the bootstrapped estimates indicating a less effective, but more costly intervention.

Over the 12 months follow-up period, the mean OOPs were PPP US$108.91 (SE PPP US$19.79) in the cytisine arm and PPP US$81.74 (SE PPP US$11.73) in the placebo arm. The main cost driver was OOP for doctor visits in both arms, while in the cytisine arm participants also spent more on hospital stays (Table 3). This pattern was consistent with costs from the healthcare provider's perspective. Productivity losses mostly occurred before and during TB treatment period and decreased considerably in the last six months of the trial. The OOP for tobacco products dropped after randomisation in both arms but remained stable throughout the 12 months period post-randomisation, which was consistent with the quit rates observed in both arms.

[Insert Table 3 here]

DISCUSSION

The intervention cost was PPP US$60.65 (SE PPP US$0.41) per participant in the cytisine arm and PPP US$12.37 (SE PPP US$0.08) per participant in the placebo arm. The difference was mainly attributed to cytisine medicine. The incremental total costs at six months post-randomisation were estimated at PPP US$57.74 (95% CI PPP US$49.40 to PPP US$83.36) while the incremental QALYs were estimated at -0.001 (95% CI -0.004 to 0.002). These results indicated that adding cytisine to brief BS for quitting smoking was unlikely to be cost-effective. The sensitivity analyses confirmed the robustness of this conclusion. Extending the time horizon to 12 months did not change the conclusion.

While the observed quit rates were not statistically significantly different between arms, participants' OOP for tobacco products on average dropped by nearly two-thirds after randomisation, indicating a reduction of tobacco consumption. The higher than expected productivity loss, OOPs for doctor visits and TB treatment before baseline might be because participants had experienced some symptoms and sought medical attention before TB was diagnosed. It was unclear, however, why the cytisine arm reported more and longer hospital stays

For peer review only - http://bmjopen.bmj.com/site/about/guidelines.xhtml

than the placebo arm, given that we did not find evidence of differential TB treatment outcomes and adverse event rates between trial arms.[9]

The strength of the study stems from the large sample size and high follow-up rates. Despite limitations of published data availability, patient level measures were collected using a comprehensive questionnaire to enable a full cost-effectiveness analysis to be undertaken. However, several limitations could potentially affect the results. Firstly, our estimated costs could be an underestimation. We observed that some health workers discussed smoking cessation during several routine TB consultations and some research assistants delivered the study drug to participants if they had missed day 5 follow-up. TB treatment costs were estimated based on simplified scenarios. Intensive treatments in the case of deterioration, death or retreatment were not considered. Costs of general medication were not included because our unit costs data source for healthcare services did not include them. However, this should not bias the results towards either arm. Secondly, the data source of unit costs of healthcare services was last updated in 2010. Certain changes may not be accounted for by simple inflation. While up-to-date data source was not available at the time of analysis, the results could be updated when it becomes available as the service use was collected in quantities. Thirdly, productivity loss in the case of death was considered zero but if a life-time observation or modelling were undertaken, productivity loss due to premature death should be included. Given the large sample size and few deaths occurred, this was unlikely to affect the conclusions.

To our knowledge, this is the first cost-effectiveness study of cytisine as a smoking cessation aid alongside an RCT and one of few for smoking cessation intervention in LMICs. A systematic review published in 2019 identified eight placebo-controlled trials and one non-inferiority trial (using nicotine replacement therapies) that used cytisine for smoking cessation, all of which were among smokers in general population and only one was conducted in LMICs.[7] Although cytisine has been identified as affordable globally [35] its cost-effectiveness in smoking cessation was based on modelled economic evaluation not empirical evidence.[36] Our study contributed to fill this gap of empirical evidence in LMICs.

In summary, our findings do not support the cost-effectiveness of adding cytisine to BS for smokers who are newly diagnosed with pulmonary TB. Future studies might explore non-medical interventions in LMICs, given the relatively lower costs of labour.

FUNDING

This work was supported by the European Union’s Horizon 2020 research and innovation programme, under Grant Agreement No. 680995. The European Commission is not responsible for any of the content of this article. The manufacturer, Aflofarm, supplied trial medication at no cost.

PATIENT AND PUBLIC INVOLVEMENT

Patient groups were consulted on the intervention materials for their lucidness during the intervention development stage. No other patient and public involvement occurred in the study process.

For peer review only - http://bmjopen.bmj.com/site/about/guidelines.xhtml

CONFLICT OF INTERESTS

Kamran Siddiqi received a research grant from Pfizer (2015-2017) to study the effects of varenicline (a smoking cessation medicine) on waterpipe smoking cessation. Daniel Kotz received an unrestricted grant from Pfizer in 2009 for an investigator-initiated trial on the effectiveness of practice nurse counselling and varenicline for smoking cessation in primary care (Dutch Trial Register NTR3067; DOI: 10.1111/add.13927). The medication for the trial were provided by Aflofarm free of charge. Aziz Sheikh is supported by Health Data Research UK's BREATHE Hub.

DATA SHARING STATEMENT

Access to partial anonymised datasets from the study can be provided upon request to KS (Chief Investigator) through corresponding author of this manuscript. Statistical code is available on GitHub (Clearice84/TBTobacco: Health economic analysis STATA code for TB&Tobacco trial (github.com)).

AUTHORS' CONTRIBUTION

JL conducted the cost-effectiveness analysis and drafted the manuscript under the supervision of SP. SP also contributed to the analysis design. AKe contributed to data management and statistical analysis, including some clinical measures used in this manuscript. OD and RG contributed to study design, conduct and interpretation of findings. AR and AMM managed the study and contributed to interpretation of findings. RH, DB, RF, AK, RZ and SM conducted the study in Bangladesh/Pakistan, collected and managed the data in countries and provided critical inputs to data analysis and interpretation. DK, EK, MB and HE provided insights to study design on aspects of behavioural support implementation, evaluation of its delivery and interpretation of findings. AS provided critical oversight to study design, trial conduct, interpretation of findings and discussion. KS conceptualised the study, contributed to the study design, conduct, and interpretation of findings.

All authors provided critical revisions and approved the final manuscript.

For peer review only

For peer review only - http://bmjopen.bmj.com/site/about/guidelines.xhtml

REFERENCES

1. Chaves Torres NM, Quijano Rodriguez JJ, Porras Andrade PS, et al. Factors predictive of the success of tuberculosis treatment: A systematic review with meta-analysis. *PloS one*. 2019;14(12):e0226507. https://www.ncbi.nlm.nih.gov/pubmed/31881023.
2. World Health Organization (WHO). Gobal Tuberculosis Report 2019. Geneva; 2019. https://www.who.int/tb/publications/global_report/en/.
3. Bangladesh Ministry of Health & Family Welfare, Bangladesh Bureau of Statistics, World Health Organization, et al. Global Adult Tobacco Survey: Bangladesh 2017 - Fact Sheet. 2018 Aug. https://www.who.int/tobacco/surveillance/survey/gats/fact-sheet-gats-bangladesh-2017.pdf?ua=1.
4. Pakistan Ministry of National Health Services Regulation and Coordination, Pakistan Bureau of Statistics, Pakistan Health Research Council, et al. Global Adult Tobacco Survey: Pakistan 2014 - Fact Sheet. 2015 19 May. https://www.who.int/tobacco/surveillance/survey/gats/pakfactsheet.pdf?ua=1.
5. World Health Organization (WHO). Tobacco control profiles - countries, territories, and areas: Tobacco Free Initiative; 2019 [cited 2020 6th July]. https://www.who.int/tobacco/surveillance/policy/country_profile/en/#P.
6. Lawson PJ, Flocke SA. Teachable moments for health behavior change: a concept analysis. *Patient Educ Couns*. 2009;76(1):25-30. https://www.ncbi.nlm.nih.gov/pubmed/19110395.
7. Tutka P, Vinnikov D, Courtney RJ, et al. Cytisine for nicotine addiction treatment: a review of pharmacology, therapeutics and an update of clinical trial evidence for smoking cessation. *Addiction*. 2019;114(11):1951-69. https://www.ncbi.nlm.nih.gov/pubmed/31240783.
8. Dogar O, Barua D, Boeckmann M, et al. The safety, effectiveness and cost-effectiveness of cytisine in achieving six-month continuous smoking abstinence in tuberculosis patients-protocol for a double-blind, placebo-controlled randomized trial. *Addiction*. 2018. https://www.ncbi.nlm.nih.gov/pubmed/29676824.
9. Dogar O, Keding A, Gabe R, et al. Cytisine for smoking cessation in patients with tuberculosis: a multicentre, randomised, double-blind, placebo-controlled phase 3 trial. *Lancet Glob Health*. 2020;8(11):e1408-e17. https://www.ncbi.nlm.nih.gov/pubmed/33069301.
10. Prices, Consumer Price Index, All items, Index [Internet]. International Monetary Fund (IMF). 2019 [cited 08/05/2019]. Available from: http://data.imf.org/regular.aspx?key=61545861 Archived at http://www.webcitation.org/78DlAn9Cu.
11. DataBank World Development Indicators [Internet]. 2019 [cited 26/06/2019]. Available from: https://databank.worldbank.org/reports.aspx?source=2&series=PA.NUS.PPP&country=PAK#.
12. Global Tuberculosis report 2018 - TB data [Internet]. WHO. 2018 [cited 20/05/2019]. Available from: https://www.who.int/tb/country/data/download/en/ Archived at: http://www.webcitation.org/78VxUbGR1.
13. Gomez GB, Dowdy DW, Bastos ML, et al. Cost and cost-effectiveness of tuberculosis treatment shortening: a model-based analysis. *BMC Infect Dis*. 2016;16(1):726. https://www.ncbi.nlm.nih.gov/pubmed/27905897.
14. National Currency per U.S. Dollar, period average [Internet]. International Monetary Fund (IMF). 2019 [cited 08/05/2019]. Available from: http://data.imf.org/regular.aspx?key=61545862 Archived at http://www.webcitation.org/78DkMANRB.
15. World Health Organization. Tobacco control country profile Pakistan. Initiative TF; 2017. https://www.who.int/tobacco/surveillance/policy/country_profile/pak.pdf?ua=1.
16. World Health Organization. Tobacco control country profile Bangladesh. Initiative TF; 2017. https://www.who.int/tobacco/surveillance/policy/country_profile/bgd.pdf?ua=1.
17. National Institute for Health and Care Excellence. Putting NICE guidance into practice - Resource impact report: Stop smoking interventions and services (NG92). 2018 March. Available at: https://www.nice.org.uk/guidance/ng92/resources/resource-impact-report-pdf-4788823645 Archived at: http://www.webcitation.org/766hqbhTb.

For peer review only - http://bmjopen.bmj.com/site/about/guidelines.xhtml

18. Curtis LA, Burns A. Unit Costs of Health and Social Care 2018. Project report. Kent: University of Kent; 2018 14th January. Available at: https://kar.kent.ac.uk/70995/1/Unit%20Costs%202018%20-%20FINAL%20with%20bookmarks%20and%20covers.pdf Archived at: http://www.webcitation.org/75n7YG9xI.
19. Bangladesh Bureau of Statistics. Report on Labour Force Survey (LFS) 2016-17. Statistics, Informatics Division MoP; 2018 January. https://www.ilo.org/surveydata/index.php/catalog/2047/download/16818.
20. Pakistan Bureau of Statistics. Labour Force Survey 2017-18. Ministry of Statistics GoP; 2018 December. http://www.pbs.gov.pk/sites/default/files//Labour%20Force/publications/lfs2017_18/Annual%20Report%20of%20LFS%202017-18.pdf.
21. Economic Analysis Evaluation Team. Country-specific inpatient and outpatient estimates in 2010 currency. World Health Organisation, Department of Health Systems G, Financing; 2010. https://www.who.int/choice/cost-effectiveness/inputs/country_inpatient_outpatient_2010.pdf?ua=1.
22. International Labour Office. International Standard Classification of Occupations: ISCO-08. Geneva: ILO; 2012.
23. The EuroQol Group. EQ-5D-5L User Guide: Basic information on how to use the EQ-5D-5L instrument (version 2.1)2015 09/10/2018. Available at: https://euroqol.org/wp-content/uploads/2016/09/EQ-5D-5L_UserGuide_2015.pdf Archived at: http://www.webcitation.org/6xHG7nFyg.
24. van Hout B, Janssen MF, Feng YS, et al. Interim scoring for the EQ-5D-5L: mapping the EQ-5D-5L to EQ-5D-3L value sets. *Value Health*. 2012;15(5):708-15. https://www.ncbi.nlm.nih.gov/pubmed/22867780.
25. IMF DataMapper - GDP per capita, current prices (Purchasing power parity; international dollars per capita) [Internet]. International Monetary Fund (IMF). 2018 [cited 4th April 2019]. Available from: https://www.imf.org/external/datamapper/PPPPC@WEO/BGD/PAK/ZWE/GBR/THA/MYS/IDN/SEQ?year=2018.
26. Richardson G, Manca A. Calculation of quality adjusted life years in the published literature: a review of methodology and transparency. *Health Econ*. 2004;13(12):1203-10. https://www.ncbi.nlm.nih.gov/pubmed/15386669.
27. Faria R, Gomes M, Epstein D, et al. A guide to handling missing data in cost-effectiveness analysis conducted within randomised controlled trials. *PharmacoEconomics*. 2014;32(12):1157-70. https://www.ncbi.nlm.nih.gov/pubmed/25069632.
28. Rubin DB. Statistical Matching Using File Concatenation with Adjusted Weights and Multiple Imputations. *J Bus Econ Stat*. 1986;4(1):87-94. <Go to ISI>://WOS:A1986AXF5400013.
29. White IR, Royston P, Wood AM. Multiple imputation using chained equations: Issues and guidance for practice. *Stat Med*. 2011;30(4):377-99. https://www.ncbi.nlm.nih.gov/pubmed/21225900.
30. Woods B, Revill P, Sculpher M, et al. Country-Level Cost-Effectiveness Thresholds: Initial Estimates and the Need for Further Research. *Value Health*. 2016;19(8):929-35. https://www.ncbi.nlm.nih.gov/pubmed/27987642.
31. Fenwick E, Claxton K, Sculpher M. Representing uncertainty: the role of cost-effectiveness acceptability curves. *Health Econ*. 2001;10(8):779-87. https://www.ncbi.nlm.nih.gov/pubmed/11747057.
32. Pattanaphesaj J, Thavorncharoensap M, Ramos-Goni JM, et al. The EQ-5D-5L Valuation study in Thailand. *Expert Rev Pharmacoecon Outcomes Res*. 2018;18(5):551-8. https://www.ncbi.nlm.nih.gov/pubmed/29958008.
33. Purba FD, Hunfeld JAM, Iskandarsyah A, et al. The Indonesian EQ-5D-5L Value Set. *PharmacoEconomics*. 2017;35(11):1153-65. https://www.ncbi.nlm.nih.gov/pubmed/28695543.

For peer review only - http://bmjopen.bmj.com/site/about/guidelines.xhtml

34. Shafie AA, Vasan Thakumar A, Lim CJ, et al. EQ-5D-5L Valuation for the Malaysian Population. *PharmacoEconomics*. 2019;37(5):715-25. https://www.ncbi.nlm.nih.gov/pubmed/30535779.
35. West R, Raw M, McNeill A, et al. Health-care interventions to promote and assist tobacco cessation: a review of efficacy, effectiveness and affordability for use in national guideline development. *Addiction*. 2015;110(9):1388-403. https://www.ncbi.nlm.nih.gov/pubmed/26031929.
36. Leaviss J, Sullivan W, Ren S, et al. What is the clinical effectiveness and cost-effectiveness of cytisine compared with varenicline for smoking cessation? A systematic review and economic evaluation. *Health Technol Assess*. 2014;18(33):1-120. https://www.ncbi.nlm.nih.gov/pubmed/24831822.

For peer review only

For peer review only - http://bmjopen.bmj.com/site/about/guidelines.xhtml

Table 1 Unit costs of TB treatment, smoking cessation services and healthcare services

**Cost items**	**Unit Cost (PPP US$, 2017/18)**		**Sources**
	**Bangladesh**	**Pakistan**	
**TB treatment**			
First-line treatment, intensive phase, including drugs	54.21 per month	108.40 per month	[11-14]
First-line treatment, continuation phase, including drugs	31.62 per month	63.24 per month	
**Smoking cessation services**			
Help or advice from public/government clinic/hospital	0.68/use	0.89/use	[11, 18-20]
Group or single counselling session at public/voluntary clinic	0.94/session	1.26/session	[11, 17, 19, 20]
**General healthcare services**			
Doctor visit	4.60/visit	6.83/visit	[10, 11, 21]
Hospital inpatient	19.06/bed-day	33.14/bed-day	[10, 11, 21]

Table 2 Results of primary and complete cases analyses at six months post-randomisation

	**Primary analysis**		**Complete case analysis**	
	**Cytisine (n=1239)**	**Placebo (n=1233)**	**Cytisine (n=1122)**	**Placebo (n=1116)**
Costs (PPP US$)	Mean (SE)		Mean (SD)	
Intervention	60.65 (0.41)	12.37 (0.08)	61.25 (13.83)	12.15 (2.69)
TB treatment	305.15 (3.36)	301.83 (3.36)	306.53 (109.96)	301.36 (108.09)
Doctor visit	3.36 (0.37)	3.10 (0.31)	3.47 (13.17)	3.14 (10.58)
Hospital stay	31.91 (7.73)	16.98 (4.41)	33.08 (275.18)	17.26 (151.58)
Smoking cessation	0.46 (0.03)	0.45 (0.03)	0.49 (1.19)	0.49 (1.13)
Overall total for six months	*401.52 (8.91)*	*334.73 (5.85)*	*404.82 (311.99)*	*334.39 (196.52)*
**PPP US$, Mean (95% CI)**				
**Adjusted incremental costs**	**57.74 (49.40 to 83.36)**		**59.49 (51.95 to 89.30)**	
	Mean (SE)		Mean (SD)	
QALYs over six months	*0.395 (0.002)*	*0.398 (0.002)*	*0.401 (0.041)*	*0.403 (0.039)*
**Mean (95% CI)**				
**Adjusted incremental QALYs**	**-0.001 (-0.004 to 0.002)**		**-0.001 (-0.003 to 0.000)**	
**ICER**	**Cytisine dominated (uncertainty see Figure 1 left)**		**Cytisine dominated (uncertainty see Figure 1 right)**	

For peer review only - http://bmjopen.bmj.com/site/about/guidelines.xhtml

Table 3 Mean Out-of-pocket payments for health-related services, productivity loss and payments for tobacco products at three time points, by arm

**PPP US$ Mean (SE)**	**Cytisine (n=1239)**	**Placebo (n=1233)**
**Six months before baseline**		
OOPs for health-related services	84.90 (7.91)	86.70 (6.80)
TB treatment	15.60 (1.69)	19.71 (3.42)
Doctor visit	62.29 (6.90)	63.96 (5.67)
Hospital stay	6.97 (2.87)	3.02 (0.80)
Smoking cessation	0.04 (0.02)	0.01 (0.01)
Productivity loss	34.01 (2.14)	30.41 (1.81)
OOPs for tobacco products	1.79 (0.14)	1.64 (0.07)
**Months 1 – 6**		
OOPs for health-related services	69.70 (10.62)	51.08 (9.32)
TB treatment	22.16 (2.51)	16.24 (1.30)
Doctor visit	29.49 (7.52)	22.65 (6.08)
Hospital stay	17.65 (5.90)	11.89 (6.53)
Smoking cessation	0.40 (0.09)	0.30 (0.06)
Productivity loss	48.83 (3.00)	43.52 (3.14)
OOPs for tobacco products	0.51 (0.03)	0.50 (0.03)
**Months 7 – 12**		
OOPs for health-related services	39.21 (16.11)	30.66 (6.72)
TB treatment	5.03 (1.43)	4.55 (0.92)
Doctor visit	13.05 (2.41)	20.42 (5.22)
Hospital stay	21.08 (15.80)	5.64 (2.89)
Smoking cessation	0.04 (0.02)	0.05 (0.02)
Productivity loss	6.06 (0.58)	8.32 (0.97)
OOPs for tobacco products	0.61 (0.03)	0.58 (0.02)

For peer review only - http://bmjopen.bmj.com/site/about/guidelines.xhtml

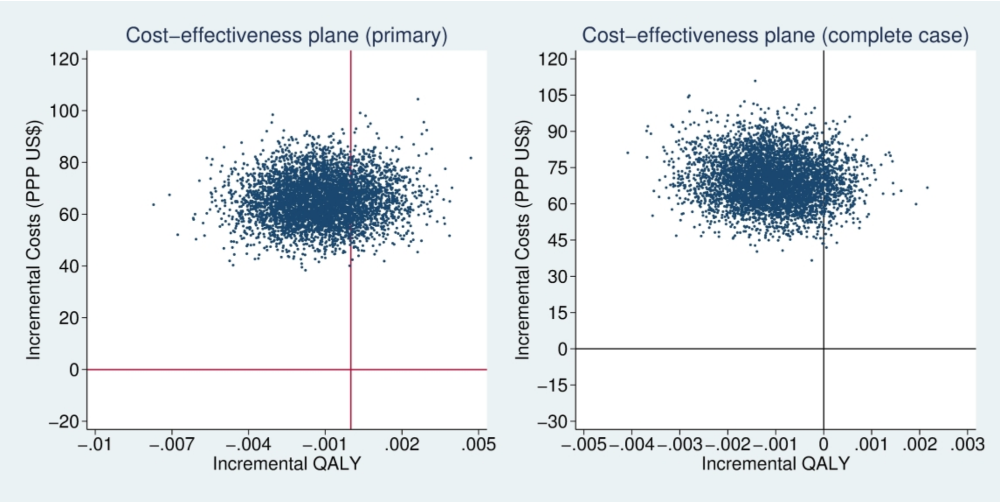

Figure 1: Cost-effectiveness plane of primary and complete case analyses at six months post-randomisation

254x127mm (300 x 300 DPI)

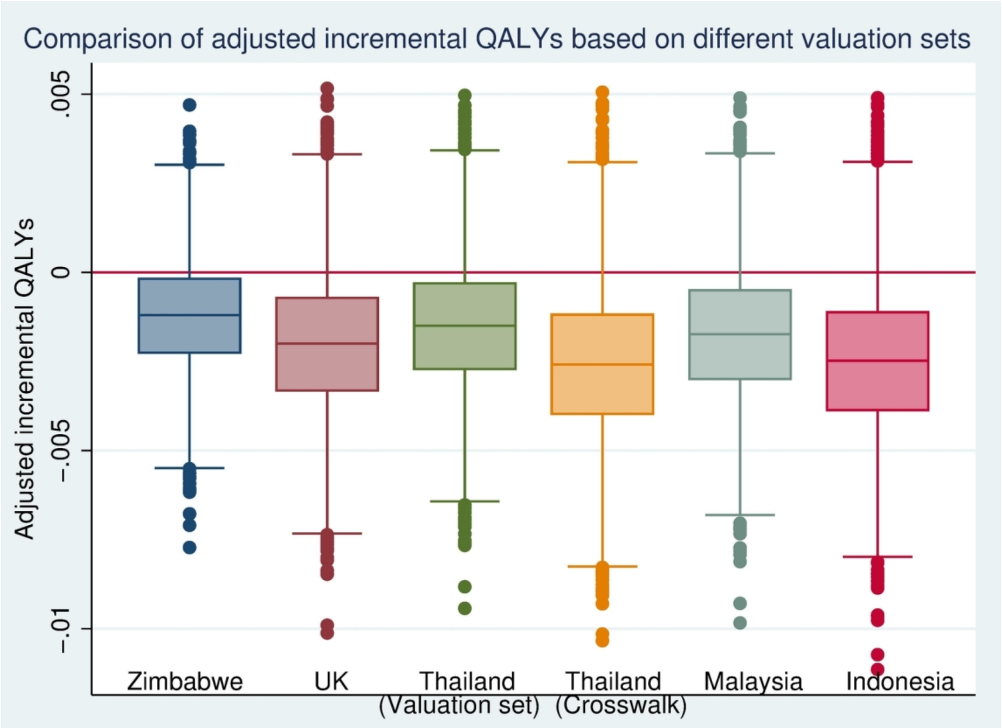

Figure 2: Comparison of adjusted incremental QALYs over six months post-randomisation derived from different methods

139x101mm (300 x 300 DPI)

Methods

Cytisine dosage schedule

The standard regimen for cytisine (Desmoxan, Aflofarm) was a 25-day course with 1.5mg hard capsules for oral administration, with six per day on days 1-3, five per day on days 4-12, four per day on days 13-16, three per day on days 17-20, two per day on days 21-24 and one on the last day.

Intervention costs

Training for the delivery of brief behavioural support was given to TB health workers before the trial began. In Bangladesh, it was a one-day training programme with a one-day refresher training and the total cost was estimated to be €4499 in 2017. In Pakistan, this consisted of a two-day training programme for DOTS facilitators and the total cost was estimated at €2324 in 2016.

In Bangladesh, the average monthly salary of a TB health worker (local salary grades G-11 to G-13) was, and average working hours per week was 48 hours.[1] In Pakistan, the average monthly salary of a TB health worker was PPP US$921.50 and average working hours per week was 47.4 hours.[2] We assumed a 30-day month as 4.3 weeks. The estimated hourly wage was therefore PPP US$3.17 in Bangladesh and PPP US$4.54 in Pakistan. The cost of BS was PPP US$0.52 for the first session and PPP US$0.26 for the second session in Bangladesh and PPP US$0.75 and PPP US$0.38 in Pakistan.

TB treatment costs

The standard treatment for pulmonary TB consisted of a two-month intensive phase and a four-month continuation phase. We extracted the overall costs of a six-month TB treatment for the two countries from the World Health Organization (WHO) TB database[3] and applied a ratio of costs of the two phases, based on a TB treatment modelling study,[4] to produce an estimate of monthly cost of intensive phase and continuation phase respectively. They were then converted to PPP US$.[5 6] The TB treatment costs were then estimated based on the participants' treatment progression on their TB registry cards.

Smoking cessation costs outside of the trial

Due to the limited smoking cessation services in the two countries,[7 8] we made assumptions on duration, based on usual practice in the UK:[9 10] 10-minute brief intervention with professionals (physician or professional nurse) for help/advice from a public/government clinic/hospital; one hour group session of 15 people or 30-minute individual session led by medical technicians/auxiliary nurses for counselling sessions in public/voluntary hospital. The ratio of group and individual sessions was assumed to be 1:1. The average hourly wage was PPP US$4.14 for "professionals" and PPP US$3.33 for "technicians and associate professionals" in Bangladesh, and PPP US$5.29 for "professionals" and PPP US$4.51 for "technicians and associate professionals" in Pakistan.[1 2 5]

General healthcare services costs

Participants' visits to a public/voluntary doctor and length of stay in a public hospital in the previous six months were collected by self-report at baseline, 6- and 12-month follow-ups. The unit costs of these services were extracted from the WHO country specific in- and out-patient costs, inflated to 2018 and converted to PPP US$.[5 11 12] The unit cost of hospital inpatient stay was the average of all hospital levels and the unit cost of a visit to doctor was the average of all settings for outpatient. These costs did not include drugs.

Out-of-pocket payments (OOPs)

Participants' spending related to following items were collected: TB treatment, public/voluntary doctor and hospital visits, and private doctor and hospital visits, including travel, smoking cessation services in public/voluntary facilities and private settings, purchasing Nicotine Replacement Therapy (NRT) or e-cigarette refills, purchasing other traditional medicine for quitting, and purchasing tobacco products.

Results

Costs

Mean training costs were PPP US$10.94 (SD PPP US$2.09) per participant in the cytisine arm and PPP US$10.92 (SD PPP US$2.09) per participant in the placebo arm. Mean cost of the information leaflet was PPP US$0.76 (SD PPP US$0.75) in the cytisine arm and PPP US$0.75 (SD PPP US$0.75) in the placebo arm. Mean cost of BS was PPP US$0.68 (SD PPP US$0.36) among 1233 participants in the cytisine arm and PPP US$0.70 (SD US$0.36) among 1226 participants in the placebo arm. Mean cost of cytisine was PPP US$48.27 (SD PPP US$12.54) while the cost of placebo was assumed at zero.

Mean costs of TB treatment were estimated to be PPP US$307.39 (SD PPP US$110.25) in the cytisine arm and PPP US$302.45 (SD PPP US$108.53) in the placebo arm, excluding 102 (8.2%) participants in the cytisine arm and 103 (8.4%) in the placebo arm who did not have information from TB cards at six-month follow-up (Table 1). The use of smoking cessation support was reported by a small group of participants in both arms. Mean costs of public/voluntary smoking cessation services were low in both arms throughout the 12 months period. Most participants reported neither visiting a doctor other than for their TB treatment nor being admitted to hospital for any reason. While mean costs of doctor visits were similar between respondents in both arms throughout the trial period, mean costs of hospital stay in the cytisine arm were nearly twice as high as in the placebo arm in months 1-6.

Out-of-pocket payments

The respondents reported an increase of spending on smoking cessation in months 1-6 compared to close to none before and after, corresponding with the intervention delivery and TB treatment period. Mean spending on tobacco was lower during the trial period than before among respondents. However, in comparison with the spending on smoking cessation, the spending on tobacco was consistently higher. The OOPs for healthcare services, including travel, loosely followed the same pattern of the costs of the services (Table 1).

Table 1 Mean (SD) costs and OOPs of TB treatment, additional smoking cessation services and general healthcare services, and OOPs on tobacco products, by arm

	**Cytisine (n=1239)**		**Placebo (n=1233)**	
	**n**	**Mean (SD) PPP US$**	**n**	**Mean (SD) PPP US$**
**TB treatment costs**				
TB registry	1137	307.39 (110.25)	1130	302.45 (108.53)
**Additional smoking cessation costs**				
Six months before baseline	1239	0.00 (0.10)	1233	0.00 (0.09)
Months 1-6	1174	0.47 (1.17)	1164	0.47 (1.11)
Months 7-12	1134	0.22 (0.75)	1144	0.21 (0.77)
**Doctor visit costs**				

Six months before baseline	1239	3.26 (14.27)	1232	3.48 (23.44)
Months 1-6	1176	3.39 (12.96)	1166	3.04 (10.39)
Months 7-12	1148	1.27 (4.73)	1157	1.12 (4.58)
**Hospital stay costs**				
Six months before baseline	1237	6.77 (57.79)	1231	4.84 (43.25)
Months 1-6	1175	31.58 (268.99)	1166	16.52 (148.33)
Months 7-12	1148	5.01 (80.30)	1157	5.87 (94.86)
**Additional smoking cessation OOPs**				
Six months before baseline	1236	0.04 (0.75)	1230	0.00 (0.09)
Months 1-6	1091	0.34 (2.72)	1080	0.28 (1.95)
Months 7-12	1110	0.05 (0.61)	1115	0.05 (0.56)
**Tobacco OOPs**				
Six months before baseline	1229	1.79 (5.05)	1224	1.64 (2.35)
Months 1-6	1177	0.50 (1.03)	1166	0.48 (0.91)
Months 7-12	1148	0.58 (0.92)	1157	0.57 (0.75)
**TB treatment OOPs**				
Six months before baseline	1238	15.45 (59.42)	1233	19.71 (119.96)
Months 1-6	1174	22.00 (85.28)	1164	15.77 (42.34)
Months 7-12	1148	5.03 (48.72)	1156	4.36 (30.50)
**Doctor visit OOPs**				
Six months before baseline	1233	61.53 (243.17)	1227	63.21 (199.10)
Months 1-6	1173	27.49 (238.38)	1158	22.07 (216.35)
Months 7-12	1148	13.28 (84.58)	1157	19.07 (162.71)
**Hospital stay OOPs**				
Six months before baseline	1237	6.91 (101.21)	1231	3.01 (28.19)
Months 1-6	1173	16.65 (200.58)	1164	11.72 (220.92)
Months 7-12	1148	17.20 (460.84)	1157	5.65 (99.21)

Productivity loss

Among the respondents, while the mean productivity loss peaked in months 1-6 as expected, it was higher than expected in the six months before baseline, most prominently reflected by productivity loss due to participants' sick leave (Table 2). This might correspond with productivity loss due to companion to TB clinic in the six months before baseline, which was consistent with participants' OOPs for TB clinic during the same period.

Table 2 Mean (SD) productivity loss of companion to TB clinic, doctor, and participants' sick leave, by arm

	**Cytisine (n=1239)**		**Placebo (n=1233)**	
	**n**	**Mean (SD) PPP US$**	**n**	**Mean (SD) PPP US$**
**Companion to TB clinic**				
Six months before baseline	*1232*	*4.62 (9.01)*	*1228*	*4.48 (7.73)*
Month 1 – 6	*1134*	*13.45 (21.55)*	*1127*	*12.43 (19.86)*
Month 7 – 12	*1145*	*2.01 (7.40)*	*1152*	*2.33 (7.19)*
**Companion to doctor**				
Six months before baseline	*1203*	*2.10 (9.15)*	*1196*	*1.87 (6.05)*
Month 1 – 6	*1126*	*3.35 (13.22)*	*1116*	*2.65 (8.44)*
Month 7 – 12	*1143*	*0.37 (2.82)*	*1151*	*0.56 (4.82)*
**Sick leave**				

For peer review only

	Cytisine (n=1239)		Placebo (n=1233)	
	**n**	**Mean (SD) PPP US$**	**n**	**Mean (SD) PPP US$**
Six months before baseline	*1230*	*27.14 (73.17)*	*1227*	*23.82 (61.12)*
Month 1 – 6	*1194*	*31.98 (100.27)*	*1171*	*28.52 (107.49)*
Month 7 – 12	*1163*	*3.62 (18.24)*	*1160*	*5.14 (28.21)*

Quality-adjusted life years

In the EQ-5D-5L descriptive system, the domains with least proportion of respondents scoring no problem were Pain/Discomfort and Anxiety/Depression at all three time points although the proportion increased after baseline (Table 3).

Table 3 Number and percentage of respondents scoring five levels of each domain of EQ-5D-5L, by arm and time point

Domains	Mobility		Self-care		Usual activities		Pain/ Discomfort		Anxiety/ Depression	
	Cytisine	Placebo	Cytisine	Placebo	Cytisine	Placebo	Cytisine	Placebo	Cytisine	Placebo
Baseline										
1	731	746	985	993	655	654	413	426	407	411
	59%	61%	79%	81%	53%	53%	33%	35%	33%	33%
2	315	291	190	163	380	373	447	462	453	463
	25%	24%	15%	13%	31%	30%	36%	38%	37%	38%
3	140	143	49	58	133	146	250	227	232	231
	11%	12%	4%	5%	11%	12%	20%	18%	19%	19%
4	50	51	12	16	55	52	114	104	112	98
	4%	4%	1%	1%	4%	4%	9%	8%	9%	8%
5	3	2	3	1	14	8	14	13	33	29
	0%	0%	0%	0%	1%	1%	1%	1%	3%	2%
Total	1239	1233	1239	1231	1237	1233	1238	1232	1237	1232
Six months										
1	985	992	1077	1078	945	960	753	778	818	829
	86%	88%	94%	95%	83%	85%	66%	69%	72%	73%
2	119	116	56	44	171	147	364	325	287	260
	10%	10%	5%	4%	15%	13%	32%	29%	25%	23%
3	25	13	7	8	19	18	19	20	28	29
	2%	1%	1%	1%	2%	2%	2%	2%	2%	3%
4	12	10	2	2	5	5	6	7	8	12
	1%	1%	0%	0%	0%	0%	1%	1%	1%	1%
5	2	1	1	0	3	1	1	1	2	1
	0%	0%	0%	0%	0%	0%	0%	0%	0%	0%
Total	1143	1132	1143	1132	1143	1131	1143	1131	1143	1131
12 months										
1	994	1020	1059	1082	968	996	755	780	826	833
	90%	91%	96%	97%	88%	89%	69%	70%	75%	75%
2	86	75	33	24	115	101	299	284	226	238

For peer review only

	8%	7%	3%	2%	10%	9%	27%	26%	21%	21%
3	11	12	6	2	12	10	35	34	33	28
	1%	1%	1%	0%	1%	1%	3%	3%	3%	3%
4	8	6	3	4	4	5	9	13	8	13
	1%	1%	0%	0%	0%	0%	1%	1%	1%	1%
5	3	2	1	1	3	1	3	2	4	1
	0%	0%	0%	0%	0%	0%	0%	0%	0%	0%
Total	1102	1115	1102	1113	1102	1113	1101	1113	1097	1113

Levels for each domain: 1=no problem, 2=slight problem, 3=moderate problem, 4=severe problem, 5=extreme problem/inability

Table 4 shows mean EQ-5D-5L utility and VAS among observed cases at baseline, 6 and 12 months follow-ups and QALYs over 6 and 12 months period. Mean utility in the cytisine arm appeared to be consistently lower than in the placebo arm at all timepoints though the difference was small. The mean QALYs were therefore lower in the cytisine arm than in the placebo arm. However, it should be noted, only those who had data on all relevant timepoints were included in calculating QALYs. The EQ-5D VAS showed a similar pattern where both arms began at similar level but in the cytisine arm, the observed cases scored slightly lower than those in the placebo arm in the follow-ups.

Table 4 Mean (SD) EQ-5D-5L utility, EQ-5D VAS and QALYs, by arm

	**Cytisine (n=1239)**		**Placebo (n=1233)**	
	**n**	**Mean (SD)**	**n**	**Mean (SD)**
**Utility**				
Baseline	1234	0.754 (0.133)	1229	0.759 (0.130)
6 months	1179	0.825 (0.165)	1164	0.831 (0.161)
12 months	1144	0.822 (0.189)	1149	0.829 (0.176)
**QALYs**				
Over 6 months	1174	0.394 (0.056)	1160	0.397 (0.054)
Over 12 months	1129	0.805 (0.134)	1122	0.810 (0.128)
**VAS**				
Baseline	1239	53.5 (15.4)	1233	53.5 (16.0)
6 months	1179	80.5 (20.3)	1165	81.3 (19.8)
12 months	1150	84.0 (21.8)	1156	84.7 (20.7)

For peer review only

References

1. Bangladesh Bureau of Statistics. Report on Labour Force Survey (LFS) 2016-17, 2018.
2. Pakistan Bureau of Statistics. Labour Force Survey 2017-18, 2018.
3. World Health Organization. Global Tuberculosis report 2018 - TB data: WHO, 2018.
4. Gomez GB, Dowdy DW, Bastos ML, et al. Cost and cost-effectiveness of tuberculosis treatment shortening: a model-based analysis. *BMC Infect Dis* 2016;16(1):726. doi: 10.1186/s12879-016-2064-3 [published Online First: 2016/12/03]
5. The World Bank. DataBank World Development Indicators, 2019.
6. International Monetary Fund. National Currency per U.S. Dollar, period average. Inernational Financial Statistics: International Monetary Fund (IMF), 2019.
7. World Health Organization. Tobacco control country profile Pakistan. Tobacco control country profiles, 2017.
8. World Health Organization. Tobacco control country profile Bangladesh. Tobacco control country profiles, 2017.
9. National Institute for Health and Care Excellence. Putting NICE guidance into practice - Resource impact report: Stop smoking interventions and services (NG92), 2018.
10. Curtis LA, Burns A. Unit Costs of Health and Social Care 2018. Kent: University of Kent, 2018.
11. Economic Analysis and Evaluation Team. Country-specific inpatient and outpatient estimates in 2010 currency: World Health Organisation, 2010.
12. International Monetary Fund. Prices, Consumer Price Index, All items, Index. International Financial Statistics (IFS): International Monetary Fund (IMF), 2019.

For peer review only

TB & Tobacco

CASE REPORT FORM - Visit (DAY 0)

Site ID: ☐☐ **Trial Number:** ☐☐☐☐☐☐ **Date of Completion:** ☐☐ / ☐☐ / ☐☐☐☐
Day *Month* *Year*

Section V

ECONOMIC EVALUATION *(This section is about the patient's wider health care use in the past six months, unless stated as TB-specific, this is for any illness.)*

Please exclude care provided by the trial intervention in your answers to these questions.

All costs should be specified in local currency, please round all costs up to the nearest whole number.

Enter a number for each item, if none, enter "0" (zero).

Please use this information to guide you if the patient gives estimates-

For daily visit: One week= 7 days, one month= 30 days, three months= 90 days, six months= 180 days.
For weekly visit per month= 4 times.

(i.e "I visited a centre daily for 6 months" would be 180 times)

1. Have you visited a TB clinic in the past six months?
(please exclude current visit and include visits to diagnostic centres if separate from clinics)

☐ Yes ☐ No (go to Q2)

If 'Yes'

a. How many times have you visited a public/voluntary TB clinic? ☐☐☐

b. How many times have you visited a private TB clinic? ☐☐☐

In **total** how much did you pay in the past six months (for consultation, diagnostics, procedures, drugs)? *(in local currency)* ☐☐☐☐☐☐

c. How much did you usually pay for your own travel per visit? *(in local currency)* ☐☐☐☐☐☐

d. On how many of these visits were you accompanied by a friend/relative? ☐☐☐

e. How much time in **total** did it usually take per visit *(travel, waiting, procedure)*? ☐☐ hours ☐☐ minutes

For peer review only - http://bmjopen.bmj.com/site/about/guidelines.xhtml

TB & Tobacco Trial Number: ☐☐☐☐☐☐

2. Have you visited a doctor in the past six months (*for any illness and exclude TB clinic visits recorded in Q1)*?

☐ Yes ☐ No (go to Q3)

If 'Yes'

a. How many times have you visited a public/voluntary doctor? ☐☐☐

In the past six months, in **total** how much did you pay for public/voluntary visits *(for consultation, diagnostics, procedure, drugs)*? *(in local currency)* ☐☐☐☐☐☐

b. How many times have you visited a private doctor? ☐☐☐

In the past six months, in **total** how much did you pay for private visits *(for consultation, diagnostics, procedure, drugs)*? *(in local currency)* ☐☐☐☐☐☐

c. How much time did you usually spend with the doctor per visit? ☐☐ hours ☐☐ minutes

d. How much did you usually pay for your own travel per visit? *(in local currency)* ☐☐☐☐☐☐

e. On how many of these visits were you accompanied by a friend/relative etc.? ☐☐☐

f. How much time in **total** did it usually take per visit *(travel, waiting, procedure)*? ☐☐ hours ☐☐ minutes

3. Have you been admitted to hospital in the past six months (*for any illness*)?

☐ Yes ☐ No (go to Q4)

If 'Yes'

a. How many nights were you in a public/voluntary hospital? ☐☐☐

In **total** how much did you pay in the past six months at public/voluntary hospitals *(for consultation, diagnostics, procedures, drugs, overnight stay)*? *(in local currency)* ☐☐☐☐☐☐

b. How many nights were you in a private hospital? ☐☐☐

In **total** how much did you pay in the past six months at private hospitals *(for consultation, diagnostics, procedures, drugs, overnight stay)*? *(in local currency)* ☐☐☐☐☐☐

c. How much did you usually pay for your own travel per visit? *(in local currency)* ☐☐☐☐☐☐

For peer review only

For peer review only - http://bmjopen.bmj.com/site/about/guidelines.xhtml

TB & Tobacco Trial Number:

4. Have you received any help to stop smoking in the past six months? *(please exclude the behavioural support session immediately before joining the trial, the session provided by the trial and any medication provided by the trial)*

☐ Yes (go to Q5) ☐ No (go to Q6)

5. How many times in the past six months have you *(this question is only about smoking cessation)*: Enter a number for each item, if none enter '0' (zero).

	Number of times	Amount spent out of pocket *(in local currency)*
Had help or advice about smoking from a public/government clinic/hospital?		
Had help or advice about smoking from a private clinic/hospital?		
Attended a group or single counselling session on smoking at a public/voluntary clinic?		
Attended a group or single counselling session on smoking at a private clinic/hospital?		
Been given a prescription for nicotine patches?		
Been given a prescription for an alternative form of NRT? (such as gum, lozenge, inhaler, etc)		
Bought a refill for an electronic cigarette?		
Been given a prescription for Zyban (Bupropion)?		
Been given a prescription for Champix (Varenicline)?		
Received any traditional medicine? (Hakeem, Homeopathic, Unani etc.)		
Other: please describe:		

For peer review only

For peer review only - http://bmjopen.bmj.com/site/about/guidelines.xhtml

TB & Tobacco

Trial Number: | | | | | | |

6. Have you received any medications for TB in the past six months?

☐ Yes (go to Q7) ☐ No (go to Q8)

7. Please detail below the medications for TB related illness in the past six months?
(Use the colour of the packets to indicate each medication)

(If patient answers not in days: one week= 7 days, one month= 30 days, three months= 90 days, six months= 180 days etc.)

**Anti-TB medication**	**Number of tablets per day**	**Duration receiving tablets (days)**
Fixed-dose combination (4 drugs) *(R-150mg/H-75mg/E-275mg/P-400mg)*		
Fixed-dose combination (2 drugs) *(R-150mg/H-75mg)*		

8. Do you have a paid job? *(include self-employed and employed) (Please tick one only)*

☐ I have a full time job (go to Q9)

☐ I have a part time job (go to Q9)

☐ I do not have a job (go to Q10)

9. Have you been off work sick in the past six months *(for any illness)*?

☐ Yes

☐ No (go to Q10)

If 'Yes' how many days were you off work sick in the last six months? | | | |

10. Usually how much did you spend **per day** on tobacco over the past six months? *(In local currency)* | | | | | | |

For peer review only - http://bmjopen.bmj.com/site/about/guidelines.xhtml

TB & Tobacco Trial Number:

EURO QOL

This section asks about your health in general.
Under each heading, please tick the ONE box that best describes your health TODAY.

MOBILITY

- I have no problems in walking about ☐
- I have slight problems in walking about ☐
- I have moderate problems in walking about ☐
- I have severe problems in walking about ☐
- I am unable to walk about ☐

SELF-CARE

- I have no problems washing or dressing myself ☐
- I have slight problems washing or dressing myself ☐
- I have moderate problems washing or dressing myself ☐
- I have severe problems washing or dressing myself ☐
- I am unable to wash or dress myself ☐

USUAL ACTIVITIES *(e.g. work, study, housework, family or leisure activities)*

- I have no problems doing my usual activities ☐
- I have slight problems doing my usual activities ☐
- I have moderate problems doing my usual activities ☐
- I have severe problems doing my usual activities ☐
- I am unable to do my usual activities ☐

PAIN/DISCOMFORT

- I have no pain or discomfort ☐
- I have slight pain or discomfort ☐
- I have moderate pain or discomfort ☐
- I have severe pain or discomfort ☐
- I have extreme pain or discomfort ☐

ANXIETY/DEPRESSION

- I am not anxious or depressed ☐
- I am slightly anxious or depressed ☐
- I am moderately anxious or depressed ☐
- I am severely anxious or depressed ☐
- I am extremely anxious or depressed ☐

UK (English) © 2009 EuroQol Group. EQ-5D™ is a trade mark of the EuroQol Group

For peer review only - http://bmjopen.bmj.com/site/about/guidelines.xhtml

TB & Tobacco

Trial Number: | | | | | | |

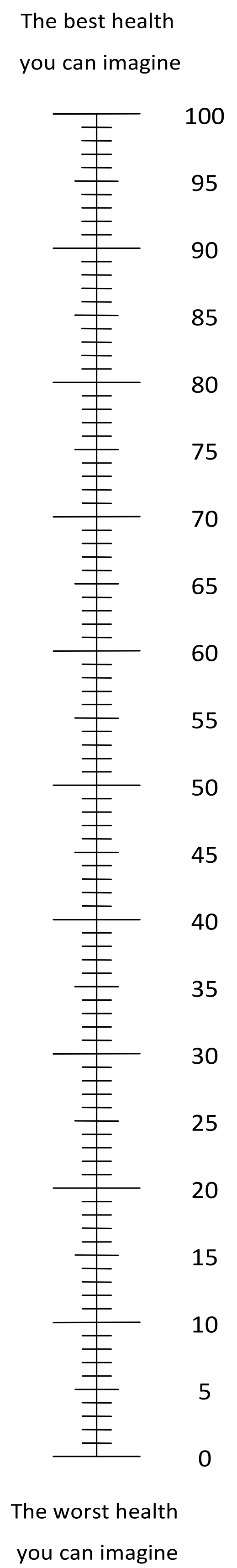

- We would like to know how good or bad your health is TODAY.
- The scale is numbered from 0 to 100.
- 100 means the best health you can imagine.

  0 means the worst health you can imagine.
- Mark an X on the scale to indicate how your health is TODAY.
- Now, please write the number you marked on the scale in the box below.

YOUR HEALTH TODAY = | | | |

For peer review only

UK (English) © 2009 EuroQol Group. EQ-5D™ is a trade mark of the EuroQol Group

Please dispense medication for 1 week. Instruct the participant to come back for follow up coinciding with their quit date and also to bring the blister packets and the 'dosing schedule card'.

Thank you for your time!

Send data

For peer review only - http://bmjopen.bmj.com/site/about/guidelines.xhtml

2624566506

Supplementary tables

Table S1 Average hourly wage by occupation in Pakistan and Bangladesh

	Average hourly wage (PPP US$) [1-3]						
	**Bangladesh**				**Pakistan**		
**Occupation**	**Male**	**Female**	**Total**		**Male**	**Female**	**Total**
Managers	5.68	5.13	5.62		9.67	0.84	9.57
Professionals	4.25	3.93	4.13		6.06	3.84	5.30
Technicians and Associate Professionals	3.35	3.21	3.32		4.69	3.27	4.50
Clerical support workers	2.56	2.33	2.53		4.69	3.16	4.66
Service and Sales workers	1.88	1.76	1.86		2.85	2.37	2.83
Skilled Agricultural, forestry and fisheries	1.50	1.24	1.46		3.03	0.98	2.96
Craft and Related Trades workers	1.69	1.55	1.65		3.00	0.89	2.67
Plant and Machine Operators, and Assembler	1.91	1.77	1.89		2.96	1.95	2.95
Elementary Occupations	1.38	1.15	1.32		2.39	1.11	2.15
Overall	2.14	1.93	2.09		3.35	2.00	3.15

For peer review only

Table S2 Number and proportion of missing values of variables by arm

	Cytisine (n=1239)		Placebo (n=1233)	
Variables	Number of missing values	Proportion of missing values	Number of missing values	Proportion of missing values
Cost of behavioural support	6	0%	7	1%
Cost of TB treatment	102	8%	103	8%
Cost of doctor visit at d0	0	0%	1	0%
Cost of doctor visit at m6	61	5%	67	5%
Cost of doctor visit at m12	89	7%	76	6%
Cost of hospital stay at d0	2	0%	2	0%
Cost of hospital stay at m6	62	5%	67	5%
Cost of hospital stay at m12	89	7%	76	6%
Cost of smoking cessation at d0	0	0%	0	0%
Cost of smoking cessation at m6	63	5%	69	6%
Cost of smoking cessation at m12	103	8%	89	7%
OOP on TB treatment at d0	1	0%	0	0%
OOP on TB treatment at m6	63	5%	69	6%
OOP on TB treatment at m12	89	7%	77	6%
OOP on smoking cessation at d0	3	0%	3	0%
OOP on smoking cessation at m6	146	12%	153	12%
OOP on smoking cessation at m12	127	10%	118	10%
OOP on doctor visit at d0	6	0%	6	0%
OOP on doctor visit at m6	64	5%	75	6%
OOP on doctor visit at m12	89	7%	76	6%
OOP on hospital stay at d0	2	0%	2	0%
OOP on hospital stay at m6	64	5%	69	6%
OOP on hospital stay at m12	89	7%	76	6%
OOP on tobacco products d0	10	1%	9	1%
OOP on tobacco products m6	60	5%	67	5%
OOP on tobacco products m12	89	7%	76	6%
Productivity loss of company for TB treatment at d0	7	1%	5	0%
Productivity loss of company for TB treatment at m6	103	8%	106	9%
Productivity loss of company for TB treatment at m12	92	7%	81	7%
Productivity loss of company for doctor at d0	36	3%	37	3%
Productivity loss of company for doctor at m6	111	9%	117	9%
Productivity loss of company for doctor at m12	94	8%	82	7%
Productivity loss of sick leave at d0	9	1%	6	0%
Productivity loss of sick leave at m6	44	4%	62	5%

For peer review only

	Cytisine (n=1239)		Placebo (n=1233)	
Variables	Number of missing values	Proportion of missing values	Number of missing values	Proportion of missing values
Productivity loss of sick leave at m12	75	6%	73	6%
EQ-5D-5L at d0				
1 Mobility	0	0%	0	0%
2 Self-care	0	0%	2	0%
3 Usual activities	2	0%	0	0%
4 Pain and discomfort	1	0%	1	0%
5 Anxiety or depression	2	0%	1	0%
EQ-5D-5L at m6				
1 Mobility	60	5%	67	5%
2 Self-care	60	5%	67	5%
3 Usual activities	60	5%	68	6%
4 Pain and discomfort	60	5%	68	6%
5 Anxiety or depression	60	5%	68	6%
EQ-5D-5L at m12				
1 Mobility	89	7%	76	6%
2 Self-care	89	7%	78	6%
3 Usual activities	89	7%	78	6%
4 Pain and discomfort	90	7%	78	6%
5 Anxiety or depression	94	8%	78	6%
VAS at d0	0	0%	0	0%
VAS at m6	60	5%	68	6%
VAS at m12	89	7%	77	6%
TB score at d0	0	0%	0	0%
TB score at m6	60	5%	66	5%

For peer review only

Table S3 Logistic regression for missingness of costs, OOPs, productivity loss and outcomes on arm and baseline covariates

Missing on:	Allocation	Age	Country
Cost of TB treatment	1.02 (0.76-1.35)	1.02 (1.01-1.03)	**0.26 (0.19-0.36)***
Cost of doctor visit at m6	1.07 (0.75-1.53)	1.00 (0.99-1.01)	**0.15 (0.10-0.24)***
Cost of doctor visit at m12	0.83 (0.61-1.14)	1.00 (0.99-1.01)	**0.17 (0.12-0.25)***
Cost of hospital stay at m6	1.05 (0.74-1.50)	1.00 (0.99-1.01)	**0.16 (0.11-0.24)***
Cost of hospital stay at m12	0.83 (0.61-1.14)	1.00 (0.99-1.01)	**0.17 (0.12-0.25)***
Cost of smoking cessation at m6	1.07 (0.76-1.52)	1.00 (0.99-1.01)	**0.17 (0.11-0.26)***
Cost of smoking cessation at m12	0.84 (0.63-1.13)	1.00 (0.99-1.01)	**0.28 (0.21-0.39)***
OOP on TB treatment at m6	1.07 (0.76-1.52)	1.00 (0.99-1.01)	**0.16 (0.10-0.24)***
OOP on TB treatment at m12	0.84 (0.62-1.15)	1.00 (0.99-1.01)	**0.17 (0.12-0.25)***
OOP on smoking cessation at m6	1.04 (0.82-1.33)	**0.99 (0.98-1.00)***	1.14 (0.89-1.47)
OOP on smoking cessation at m12	0.91 (0.70-1.18)	0.99 (0.98-1.00)	**0.52 (0.40-0.68)***
OOP on doctor visit at m6	1.15 (0.82-1.62)	1.00 (0.99-1.01)	**0.16 (0.10-0.24)***
OOP on doctor visit at m12	0.83 (0.61-1.14)	1.00 (0.99-1.01)	**0.17 (0.12-0.25)***
OOP on hospital stay at m6	1.05 (0.74-1.49)	1.00(0.99-1.01)	**0.16 (0.11-0.24)***
OOP on hospital stay at m12	0.83 (0.61-1.14)	1.00 (0.99-1.01)	**0.17 (0.12-0.25)***
OOP on tobacco products m6	1.09 (0.76-1.55)	1.00 (0.99-1.01)	**0.16 (0.10-0.24)***
OOP on tobacco products m12	0.83 (0.61-1.14)	1.00 (0.99-1.01)	**0.17 (0.12-0.25)***
Productivity loss of company for TB treatment at m6	1.01 (0.77-1.35)	0.99 (0.98-1.00)	**0.55 (0.41-0.73)***
Productivity loss of company for TB treatment at m12	0.86 (0.63-1.16)	1.00 (0.99-1.01)	**0.18 (0.13-0.26)***
Productivity loss of company for doctor at m6	1.04 (0.80-1.37)	0.99 (0.98-1.00)	**0.52 (0.40-0.69)***
Productivity loss of company for doctor at m12	0.85 (0.63-1.15)	1.00 (0.99-1.01)	**0.18 (0.12-0.25)***
Productivity loss of sick leave at m6	1.40 (0.95-2.08)	0.99 (0.97-1.00)	**0.22 (0.14-0.33)***
Productivity loss of sick leave at m12	0.95 (0.68-1.32)	0.99 (0.98-1.00)	**0.27 (0.19-0.38)***
EQ-5D-5L at m6	1.09 (0.76-1.55)	1.00 (0.99-1.01)	**0.16 (0.10-0.24)***
EQ-5D-5L at m12	0.83 (0.61-1.14)	1.00 (0.99-1.01)	**0.17 (0.12-0.25)***
TB score at m6	1.02 (0.72-1.44)	1.00 (0.99-1.01)	**0.18 (0.12-0.26)***

***P<0.05**

For peer review only

References

1. Bangladesh Bureau of Statistics. Report on Labour Force Survey (LFS) 2016-17, 2018.
2. Pakistan Bureau of Statistics. Labour Force Survey 2017-18, 2018.
3. The World Bank. DataBank World Development Indicators, 2019.

For peer review only

CHEERS Checklist

Items to include when reporting economic evaluations of health interventions

The **ISPOR CHEERS Task Force Report**, *Consolidated Health Economic Evaluation Reporting Standards (CHEERS)—Explanation and Elaboration: A Report of the ISPOR Health Economic Evaluations Publication Guidelines Good Reporting Practices Task Force*, provides examples and further discussion of the 24-item CHEERS Checklist and the CHEERS Statement. It may be accessed via the *Value in Health* or via the ISPOR Health Economic Evaluation Publication Guidelines – CHEERS: Good Reporting Practices webpage: http://www.ispor.org/TaskForces/EconomicPubGuidelines.asp

Section/item	Item No	Recommendation	Reported on page No/ line No
**Title and abstract**			
Title	1	Identify the study as an economic evaluation or use more specific terms such as "cost-effectiveness analysis", and describe the interventions compared.	P1 L1-4
Abstract	2	Provide a structured summary of objectives, perspective, setting, methods (including study design and inputs), results (including base case and uncertainty analyses), and conclusions.	P2
**Introduction**			
Background and objectives	3	Provide an explicit statement of the broader context for the study. Present the study question and its relevance for health policy or practice decisions.	P4 L2-23
**Methods**			
Target population and subgroups	4	Describe characteristics of the base case population and subgroups analysed, including why they were chosen.	P4 L35-P5 L7
Setting and location	5	State relevant aspects of the system(s) in which the decision(s) need(s) to be made.	P4 6-12, P5 L3-5
Study perspective	6	Describe the perspective of the study and relate this to the costs being evaluated.	P4 L20-23, P7 L6-11
Comparators	7	Describe the interventions or strategies being compared and state why they were chosen.	P5 L10-17
Time horizon	8	State the time horizon(s) over which costs and consequences are being evaluated and say why appropriate.	P7 L6, P4 L6-8
Discount rate	9	Report the choice of discount rate(s) used for costs and outcomes and say why appropriate.	P7 L11-12
Choice of health outcomes	10	Describe what outcomes were used as the measure(s) of benefit in the evaluation and their relevance for the type of analysis performed.	P6 L23-35, P7 L6
Measurement of effectiveness	11a	*Single study-based estimates:* Describe fully the design features of the single effectiveness study and why the single study was a sufficient source of clinical effectiveness data.	P4 L14-30

For peer review only

	11b	*Synthesis-based estimates:* Describe fully the methods used for identification of included studies and synthesis of clinical effectiveness data.	N/A
Measurement and valuation of preference based outcomes	12	If applicable, describe the population and methods used to elicit preferences for outcomes.	P6 L31-35
Estimating resources and costs	13a	*Single study-based economic evaluation:* Describe approaches used to estimate resource use associated with the alternative interventions. Describe primary or secondary research methods for valuing each resource item in terms of its unit cost. Describe any adjustments made to approximate to opportunity costs.	P5 L19-P6 L22, Supporting information 1
	13b	*Model-based economic evaluation:* Describe approaches and data sources used to estimate resource use associated with model health states. Describe primary or secondary research methods for valuing each resource item in terms of its unit cost. Describe any adjustments made to approximate to opportunity costs.	N/A
Currency, price date, and conversion	14	Report the dates of the estimated resource quantities and unit costs. Describe methods for adjusting estimated unit costs to the year of reported costs if necessary. Describe methods for converting costs into a common currency base and the exchange rate.	P5 L19-23
Choice of model	15	Describe and give reasons for the specific type of decision-analytical model used. Providing a figure to show model structure is strongly recommended.	N/A
Assumptions	16	Describe all structural or other assumptions underpinning the decision-analytical model.	N/A
Analytical methods	17	Describe all analytical methods supporting the evaluation. This could include methods for dealing with skewed, missing, or censored data; extrapolation methods; methods for pooling data; approaches to validate or make adjustments (such as half cycle corrections) to a model; and methods for handling population heterogeneity and uncertainty.	P6 L38-P7 L36
**Results**			
Study parameters	18	Report the values, ranges, references, and, if used, probability distributions for all parameters. Report reasons or sources for distributions used to represent uncertainty where appropriate. Providing a table to show the input values is strongly recommended.	Table 1, Table S1, Supporting information 1
Incremental costs and outcomes	19	For each intervention, report mean values for the main categories of estimated costs and outcomes of interest, as well as mean differences between the comparator groups. If applicable, report incremental cost-effectiveness ratios.	P8 L15-25, Table 2
Characterising uncertainty	20a	*Single study-based economic evaluation:* Describe the effects of sampling uncertainty for the estimated incremental cost and incremental effectiveness parameters, together with the impact	P8 L28-40, Figure 1-2

For peer review only

		of methodological assumptions (such as discount rate, study perspective).	
	20b	*Model-based economic evaluation:* Describe the effects on the results of uncertainty for all input parameters, and uncertainty related to the structure of the model and assumptions.	N/A
Characterising heterogeneity	21	If applicable, report differences in costs, outcomes, or cost-effectiveness that can be explained by variations between subgroups of patients with different baseline characteristics or other observed variability in effects that are not reducible by more information.	N/A
**Discussion**			
Study findings, limitations, generalisability, and current knowledge	22	Summarise key study findings and describe how they support the conclusions reached. Discuss limitations and the generalisability of the findings and how the findings fit with current knowledge.	P9 L21-P10 L9
**Other**			
Source of funding	23	Describe how the study was funded and the role of the funder in the identification, design, conduct, and reporting of the analysis. Describe other non-monetary sources of support.	P10 L21-24
Conflicts of interest	24	Describe any potential for conflict of interest of study contributors in accordance with journal policy. In the absence of a journal policy, we recommend authors comply with International Committee of Medical Journal Editors recommendations.	P10 L29-37

For consistency, the CHEERS Statement checklist format is based on the format of the CONSORT statement checklist

The **ISPOR CHEERS Task Force Report** provides examples and further discussion of the 24-item CHEERS Checklist and the CHEERS Statement. It may be accessed via the *Value in Health* link or via the ISPOR Health Economic Evaluation Publication Guidelines – CHEERS: Good Reporting Practices webpage: http://www.ispor.org/TaskForces/EconomicPubGuidelines.asp

The citation for the CHEERS Task Force Report is:
Husereau D, Drummond M, Petrou S, et al. Consolidated health economic evaluation reporting standards (CHEERS)—Explanation and elaboration: A report of the ISPOR health economic evaluations publication guidelines good reporting practices task force. Value Health 2013;16:231-50.

For peer review only

BMJ Open

Cost-utility of cytisine for smoking cessation over and above behavioural support in people with newly diagnosed pulmonary tuberculosis: An economic evaluation of a multi-centre randomised controlled trial

Journal:	*BMJ Open*
Manuscript ID	bmjopen-2021-049644.R1
Article Type:	Original research
Date Submitted by the Author:	16-Dec-2021
Complete List of Authors:	Li, Jinshuo; University of York Department of Health Sciences<br>Parrott, Steve; University of York Department of Health Sciences<br>Keding, Ada; University of York Department of Health Sciences<br>Dogar, Omara; University of York Department of Health Sciences; The University of Edinburgh Usher Institute of Population Health Sciences and Informatics<br>Gabe, Rhian; Queen Mary University of London, Centre for Cancer Prevention, Wolfson Institute of Preventive Medicine<br>Marshall, Anna-Marie; University of York Department of Health Sciences<br>Huque, Rumana; University of Dhaka Department of Economics; ARK Foundation<br>Barua, Deepa; ARK Foundation<br>Fatima, Razia; Global Fund Grant, TB/HIV and Malaria Common Management Unit<br>Khan, Amina; The Initiative<br>Zahid, Raana; The Initiative<br>Mansoor, Sonia; Rawalpindi Medical University, Institute of Psychiatry<br>Kotz, Daniel; Heinrich-Heine-Universitat Dusseldorf, Institute of General Practice, Addiction Research and Clinical Epidemiology Unit; The University of Edinburgh Usher Institute of Population Health Sciences and Informatics<br>Boeckmann, Melanie; Heinrich-Heine-Universitat Dusseldorf, Institute of General Practice, Addiction Research and Clinical Epidemiology Unit; Bielefeld University, School of Public Health, Department of Environment and Health<br>Elsey, Helen ; University of York Department of Health Sciences<br>Kralikova, Eva; Charles University, Institute of Hygiene and Epidemiology; General University Hospital in Prague, 3rd Medical Department<br>Readshaw, Anne; University of York Department of Health Sciences<br>Sheikh, Aziz; The University of Edinburgh Usher Institute of Population Health Sciences and Informatics<br>Siddiqi, Kamran; University of York Department of Health Sciences; Hull York Medical School
**Primary Subject Heading**:	Health economics

Secondary Subject Heading:	Global health, Health economics, Smoking and tobacco, Public health
Keywords:	Tuberculosis < INFECTIOUS DISEASES, HEALTH ECONOMICS, Clinical trials < THERAPEUTICS, International health services < HEALTH SERVICES ADMINISTRATION & MANAGEMENT, PUBLIC HEALTH

SCHOLARONE™ Manuscripts

I, the Submitting Author has the right to grant and does grant on behalf of all authors of the Work (as defined in the below author licence), an exclusive licence and/or a non-exclusive licence for contributions from authors who are: i) UK Crown employees; ii) where BMJ has agreed a CC-BY licence shall apply, and/or iii) in accordance with the terms applicable for US Federal Government officers or employees acting as part of their official duties; on a worldwide, perpetual, irrevocable, royalty-free basis to BMJ Publishing Group Ltd ("BMJ") its licensees and where the relevant Journal is co-owned by BMJ to the co-owners of the Journal, to publish the Work in this journal and any other BMJ products and to exploit all rights, as set out in our licence.

The Submitting Author accepts and understands that any supply made under these terms is made by BMJ to the Submitting Author unless you are acting as an employee on behalf of your employer or a postgraduate student of an affiliated institution which is paying any applicable article publishing charge ("APC") for Open Access articles. Where the Submitting Author wishes to make the Work available on an Open Access basis (and intends to pay the relevant APC), the terms of reuse of such Open Access shall be governed by a Creative Commons licence – details of these licences and which Creative Commons licence will apply to this Work are set out in our licence referred to above.

Other than as permitted in any relevant BMJ Author's Self Archiving Policies, I confirm this Work has not been accepted for publication elsewhere, is not being considered for publication elsewhere and does not duplicate material already published. I confirm all authors consent to publication of this Work and authorise the granting of this licence.

For peer review only

Cost-utility of cytisine for smoking cessation over and above behavioural support in people with newly diagnosed pulmonary tuberculosis: An economic evaluation of a multi-centre randomised controlled trial

Jinshuo Li, M.Phil[1], Steve Parrott, MSc[1], Ada Keding, MSc[1], Omara Dogar, PhD[1,2], Rhian Gabe, PhD[3], Anna-Marie Marshall, PhD[1], Rumana Huque, PhD[4, 5], Deepa Barua, MPH[4], Razia Fatima, PhD[6], Amina Khan, MPH[7], Raana Zahid, MPH[7], Sonia Mansoor, MSc[8], Daniel Kotz, PhD[2,9,10], Melanie Boeckmann, DrPH[1,9,11], Helen Elsey, PhD, FFPH[1], Eva Kralikova, PhD, MD[12, 13], Anne Readshaw, PhD[1], Aziz Sheikh, MD[2] & Kamran Siddiqi, PhD[1,14], *on behalf of the TB & Tobacco Consortium*

Authors' affiliations

[1]University of York, Department of Health Sciences, Faculty of Sciences, YO10 5DD, York, UK

[2]Usher Institute, The University of Edinburgh, EH8 9AG, Edinburgh, UK

[3] Centre for Cancer Prevention, Wolfson Institute of Preventive Medicine, Queen Mary University of London, United Kingdom

[4]ARK Foundation, Dhaka, Bangladesh

[5]Department of Economics, University of Dhaka, Bangladesh

[6]TB/HIV and Malaria Common Management Unit, Global Fund Grant, Islamabad, Pakistan

[7]The Initiative, Islamabad, Pakistan

[8] Institute of Psychiatry (IOP) Rawalpindi Medical University, Pakistan

[9]Institute of General Practice, Addiction Research and Clinical Epidemiology Unit, Medical Faculty of the Heinrich-Heine-University, Düsseldorf, Germany

[10]Department of Family Medicine, CAPHRI School for Public Health and Primary Care, Maastricht University, The Netherlands

[11]Bielefeld University, School of Public Health, Department of Environment and Health, Bielefeld, Germany

[12]3rd Medical Department, First Faculty of Medicine, Charles University, and the General University Hospital in Prague, Czech Republic

[13]Institute of Hygiene and Epidemiology, First Faculty of Medicine, Charles University, and the General University Hospital in Prague, Czech Republic

[14]Hull York Medical School, University of York, YO10 5DD, York, UK

For peer review only - http://bmjopen.bmj.com/site/about/guidelines.xhtml

Corresponding author: Jinshuo Li, e-mail: jinshuo.li@york.ac.uk, tel: +44 1904 32 1358, Area 4 ARRC building, Department of Health Sciences, University of York, Heslington, YO10 4DU, York, United Kingdom

Word count: 3880

For peer review only

For peer review only - http://bmjopen.bmj.com/site/about/guidelines.xhtml

Abstract

Objectives

To assess the cost-effectiveness of cytisine over and above brief behavioural support (BS) for smoking cessation among newly diagnosed pulmonary tuberculosis (TB) patients in low- and middle-income countries.

Design

An incremental cost-utility analysis was undertaken alongside a 12-month double-blind two-arm individually randomised controlled trial from a public/voluntary healthcare sector perspective, with the primary endpoint at six months post-randomisation.

Setting

Thirty-two sites across two countries: 17 sub-district hospitals in Bangladesh and 15 secondary care hospitals in Pakistan.

Participants

Adults (aged ≥18 years in Bangladesh and ≥15 years in Pakistan) with pulmonary TB diagnosed within the last four weeks, who smoked tobacco daily (n=2472).

Interventions

Two brief smoking cessation BS sessions with a trained TB health worker were offered to all participants. Participants in the intervention arm (n=1239) were given cytisine (25-day course) while those in the control arm (n=1233) were given placebo.

Primary and secondary outcome measures

Costs of cytisine and BS sessions were estimated based on research team records. TB treatment costs were estimated based on TB registry records. Additional smoking cessation and healthcare costs and EQ-5D-5L data were collected at baseline, six- and 12-month follow-ups. Costs were presented in Purchasing Power Parity (PPP) adjusted US dollars (US$). Quality-adjusted life years (QALYs) were derived from the EQ-5D-5L. Incremental total costs and incremental QALYs were estimated using regressions adjusting for respective baseline values and other baseline covariates. Uncertainty was assessed using bootstrapping.

Results

Mean total costs were PPP US$57.74 (95% CI 49.40 – 83.36) higher in the cytisine arm than in the placebo arm while the mean QALYs were -0.001 (95% CI -0.004 – 0.002) lower over six months, hence the cytisine arm was dominated by the placebo arm.

Conclusions

Cytisine *plus* BS for smoking cessation among TB patients was dominated by placebo *plus* BS.

Clinical trial registration: International Standard Randomized Clinical Trial Number, ISRCTN43811467

For peer review only - http://bmjopen.bmj.com/site/about/guidelines.xhtml

Strength and limitations of this study

- Large sample size and high follow-up rate ensures robustness of the conclusion
- Comprehensive patient-level data collection provides possibilities of further exploration or updating of the analyses
- Trial across two countries posed challenges to value both costs and quality-adjusted life years comparably
- Lack of up-to-date data sources of unit costs of healthcare services may affect the accuracy of the costs estimation
- Eagerness of local staff participating the trial may affect the generalisability of the intervention delivery

For peer review only

INTRODUCTION

In 2020, due to the impact of COVID-19 pandemic, the number of newly diagnosed tuberculosis (TB) case notifications saw a big drop from 2019 while the number of people died from TB increased at global, regional, and country levels.[1] Bangladesh (218 per 100,00 population) and Pakistan (259 per 100,000 population) are among the 16 countries that contributed most to the global shortfall of TB notifications yet they are still on the World Health Organization high-burden countries lists for TB and multidrug-resistant TB or rifampicin-resistant TB.[1, 2] Meanwhile, the 2020 estimates of current tobacco smoking rates were 18.5% in Bangladesh and 24.6% in Pakistan, with considerable imbalance between male and female.[3] Previous evidence suggests that continued tobacco smoking among TB patients is associated with unfavourable TB treatment outcomes.[4] However, with the combined burden of TB and tobacco, support for smoking cessation for TB patients is absent in both countries.[5]

TB treatment, lasting six months or longer, offers an opportunity for regular support for quitting smoking, if integrated properly. Newly diagnosed TB patients who smoke might be more receptive to advice to quit due to their immediate health concerns.[6] Due to limited resources, evidence-based approaches such as behavioural support (BS) and expensive pharmacotherapies for smoking cessation cannot be implemented in many low- and middle-income countries (LMICs). In the present study, we adopted a brief BS integrated with routine TB appointment for smoking cessation that was developed in collaboration with local teams in Bangladesh and Pakistan as part of standard usual care.[7] Over-and-above the BS, we examined the effectiveness and cost-effectiveness of the relatively low cost pharmacotherapy cytisine for smoking cessation in TB patients.[8]

We conducted a 12-month, two-arm, parallel, double blind, placebo-controlled, multicentre, individually randomised trial in Bangladesh and Pakistan to compare cytisine plus BS for smoking cessation (cytisine arm: n=1239) with placebo plus BS (placebo arm: n=1233) among pulmonary TB patients who smoke daily.[9] Biochemically-verified continuous abstinence at 6 months (primary endpoint) was 32.4% (401/1239) in the cytisine arm and 29.7% (366/1233) in the placebo arm (RR=1.09, 95% CI 0.97-1.23) and at 12 months, it was 24.9% (309/1239) and 22.3% (275/1233), respectively (RR=1.22, 95% CI 0.95-5.98).[10] This article reports a set of analyses to, respectively: 1) evaluate the cost-utility, from a public or voluntary healthcare sector perspective, of adding cytisine to BS for smoking cessation in TB patients who smoke; and 2) assess the financial burden in relation to tobacco use and healthcare from participants and their families' perspective, and productivity loss from a societal perspective.

METHODS

Design

An incremental cost-utility analysis was conducted alongside the randomised controlled trial (RCT) described above and elsewhere.[9, 10] The scheduled follow-ups were at 6 and 12 months post-randomisation, with 6 months as the primary endpoint. Neither participants nor TB health workers were aware of participants' arm allocation. Allocation was not revealed to health economists until database lock. Detailed information on procedures was provided in study protocol.[9]

Ethical approval was granted by the Health Sciences Research Governance Committee (HSRGC) at the University of York, UK (HSRGC/2016/144/B), the National Bioethics Committee, Pakistan Medical

For peer review only - http://bmjopen.bmj.com/site/about/guidelines.xhtml

Research Council (no. 4–87/16/NBC-200 Part-B/RDC/4197) and the National Research Ethics Committee, Bangladesh Medical Research Council (BMRC/NREC/2016–2019/1475).

Participants

Adults (aged ≥18 in Bangladesh and ≥15 in Pakistan) with pulmonary TB diagnosed within the last four weeks who smoked tobacco on a daily basis and were interested in quitting, were eligible.[9] We excluded those who were diagnosed with TB complications (retreatment or any drug resistance), extra-pulmonary TB, receiving Streptomycin and/or Para Amino Salicylic Acid, using any pharmacotherapy for tobacco dependence, pregnant or planning to become pregnant, lactating, or suffering from schizophrenia or known to be diagnosed with epilepsy. Those who had myocardial infarction, stroke, or an attack of severe angina within the previous two weeks, uncontrolled high blood pressure despite being on medication, or severe renal impairment (requiring dialysis), were also excluded.

Between June 2017 and April 2018, 1527 participants from 17 sub-district hospitals in Bangladesh and 945 participants from 15 secondary care hospitals in Pakistan were randomised to the cytisine arm (n=1239) and the placebo arm (n=1233). The mean age was 42.5 (SD 14.3) years in the cytisine arm and 42.4 (SD 14.2) years in the placebo arm. Males made up 99% of each arm (1227 in the cytisine arm, 1221 in the placebo arm). By 6 months follow-up, 70 participants died (36 in the cytisine arm and 34 in the placebo arm). A further 21 participants died after 6 months (13 in the cytisine arm and eight in the placebo arm).

Intervention and comparator

Participants in the cytisine (intervention) arm were provided with cytisine (Desmoxan, Aflofarm) according to its standard regimen: 38 capsules on day 0 and another 62 capsules on day 5 (pre-set quit date), totalling 100 capsules over a 25-day course. The trial medication was in the form of 1.5mg hard capsules for oral administration.[9, 10]. Participants in the placebo (comparator) arm were given placebo capsules with identical appearance on the same dispensing schedule. In addition, participants in both arms were offered brief BS for smoking cessation delivered by trained TB health workers, accompanied with a leaflet containing information on tobacco use and its interactions with TB for each participant. The BS was designed to be two face-to-face sessions on days 0 (10 minutes) and 5 (5 minutes). Therefore, the intervention consisted of cytisine plus BS while the comparator was placebo plus BS.

Measures

All monetary outcomes were collected or valued in local currencies and inflated to their respective 2018 values [11] where necessary, and converted to purchasing power parity adjusted US dollars (PPP US$) using the World Bank exchange rate in the same year (1 PPPUS$ = 30.9 Bangladeshi Taka = 29.3 Pakistani Rupees).[12] PPP US$ accounts for the price and income difference between the two countries so that the monetary outcomes could be pooled together. Results of costs were presented in PPP US$ 2018 price.

Costs

Intervention costs

Intervention costs included costs of training and delivery (Details see Supplementary file 1). TB health workers were trained in brief BS for smoking cessation in a two-day programme. The costs of training were estimated by the research team to be PPP US$14,183 in Bangladesh and PPP

For peer review only - http://bmjopen.bmj.com/site/about/guidelines.xhtml

US$12,837 in Pakistan. Since all participants were scheduled to receive BS, the training cost was allocated to each participant evenly.

The uptake of BS was recorded on the Case Report Form (CRF) on day 0. Staff costs for BS were estimated by multiplying the duration by the hourly wage rate. The cost of BS for the first and second session was PPP US$0.52 and PPP US$0.26 in Bangladesh, and PPP US$0.75 and PPP US$0.38 in Pakistan. For those whose CRF showed not taking up BS, the cost of BS delivery was considered null. For those who accepted BS, the cost of the first session was applied and the cost of the second session was added provided they attended the follow-up on day 5. The smoking cessation information leaflet costed PPP US$0.16 in Bangladesh and PPP US$1.71 in Pakistan, per participant.

The manufacturer provided the distributor price as 72.63 Polish Złoty for 100 capsule pack (PPP US$42.27 in Bangladesh, PPP US$65.09 in Pakistan). By dispensing schedule, the medication dispensed on day 0 costed PPP US$16.05 in Bangladesh and PPP US$24.74 in Pakistan, and on day 5 it costed PPP US$26.21 and PPP US$40.34, respectively. The placebo capsules were assumed to incur no cost. All participants had at least the first dispense and those who missed follow-up on day 5 were assumed not to receive the second dispense.

Costs of TB treatment, additional smoking cessation help, and general healthcare services

Table 1 presents the unit costs of TB treatment by phase, additional smoking cessation services, and general healthcare services, estimated based on secondary sources and some assumptions and converted to PPP US$ 2018.[12-22] Detailed methods of estimation see Supplementary file 1. TB treatment progression was estimated according to the TB registry card. The quantities of services use were collected by self-report at baseline, 6- and 12-month follow-ups (See Supplementary file 2 for CRF).

[Insert Table 1 here]

Out-of-pocket payments (OOPs) and productivity loss

Participants reported any spending in monetary form related to TB treatment, smoking cessation products, and general healthcare services use, including travel, on CRFs at baseline, 6- and 12-month follow-ups.

CRFs also collected participants' time spent in TB clinic and doctor visits, including travel and waiting time, and if and how many times they were accompanied by a friend or relative. The productivity loss of a companion was estimated by multiplying the overall time spent by the companion by societal average hourly wage in the country.[20, 21] We assumed that all companions were employed. Participants' productivity loss was estimated based on their self-reported duration of sick leave from work. Participants' hourly wage were extracted from secondary sources based on their occupation category and gender,[20, 21] with those reported in open question re-classified according to the International Standard Classification of Occupations ISCO-08 (Supplementary file 3 Table S1).[23] Those who were unemployed, retired, students or home makers, were assumed to incur no productivity loss in the case of sick leave.

Quality-adjusted life years (QALYs)

The EQ-5D-5L developed by the EuroQol Group was used to measure health-related quality of life,[24] at baseline, 6- and 12-month follow-ups, as part of the CRFs. The EQ-5D-5L consists of a descriptive system of five domains (Mobility, Self-care, Usual activities, Pain/Discomfort and Anxiety/Depression), and a Visual Analogue Scale (VAS) valuing the overall health on the day. The

For peer review only - http://bmjopen.bmj.com/site/about/guidelines.xhtml

VAS score ranges from 0 (death) to 100 (perfect health). Each domain of the descriptive system has five levels of capacity, ranging from having no problem to having severe problems. A complete descriptive system could be converted to a utility value using an appropriate tariff.

In the absence of country-specific valuation sets for Bangladesh and Pakistan, we used the valuation set of Zimbabwe based on crosswalk function to calculate utility,[25] as its Gross Domestic Product per capita in PPP US$ (2,381.22) was the closest to that of the two countries of interest (Bangladesh: 4,598.39; Pakistan: 5,714.03) at the time of the analysis.[26] QALYs were derived using the area under the curve approach.[27]

Analyses

All analyses were performed using STATA 16.0 SE.

Missing data

For the baseline covariates, missing values were imputed by the mean of the variable in the pooled sample in the same country. This was the information that was unrelated to the intervention and the randomisation functioned to balance the two arms.[28] The missing values in the follow-up variables were handled using multiple imputation method, following Rubin's rule and assuming missing at random (MAR),[29] unless it was due to death. Missing values due to death were replaced with zero or not applicable (n/a) depending on the nature of variable. An imputation model was developed to include all the variables necessary for the analysis and the number of imputations was set as approximately the highest percentage figure of missing data.[30] The imputation was performed by trial arms and on condition of being alive.

Primary analysis

The primary analysis was an incremental cost-utility analysis over six months post-randomisation from public or voluntary healthcare sector perspective. This included service providers that were classified as government, non-profit organisations, and charitable organisations. It was undertaken on an intention-to-treat basis, including all randomised participants in the arms to which they were allocated.

Total costs at 6 months consisted of intervention costs, TB treatment costs, additional public/voluntary smoking cessation costs, and public/voluntary healthcare services costs in the six months post-randomisation. Mean total costs and mean QALYs were estimated for each arm and no discounting was applied for the six months period. Incremental mean total costs and incremental mean QALYs was estimated by a mixed effect generalised linear regression model, adjusting for their respective baseline values (total costs in the six months before randomisation for total costs; baseline EQ-5D-5L utility for QALYs), age, gender, country, with study site as random-effects. An incremental cost-effectiveness ratio (ICER) was calculated by dividing the incremental mean total costs by the incremental mean QALYs.

Since there are no official willingness-to-pay (WTP) thresholds in either Bangladesh or Pakistan, the estimated WTPs for Bangladesh and Pakistan based on income elasticity of value of health, inflated to 2018 (maximum WTP: Bangladesh: PPP US$1,473 per QALY gained; Pakistan: PPP US$2,431 per QALY gained), were used to compare with the ICERs, if applicable.[31]

Because neither costs nor QALYs were normally distributed, we used a non-parametric bootstrap technique to assess the uncertainty, generating 5000 replicate samples. The results were used to construct 95% confidence intervals (CIs) of the incremental costs and QALYs. They were then plotted

For peer review only - http://bmjopen.bmj.com/site/about/guidelines.xhtml

on a cost-effectiveness plane (CEP) to demonstrate the uncertainty surrounding the ICER. Cost-effectiveness acceptability curves (CEACs) were constructed from these bootstrapped replicates by converting ICER to net monetary benefit.[32]

A separate cost-effectiveness analysis using smoking abstinence rate at six months follow-up as effect measure was planned but not undertaken because no statistically significant difference was found between arms for this outcome measure per pre-specified effect size.[10] Given that it is not clinically effective, it could not be cost-effective using this measure.

Sensitivity analyses

We undertook a complete case analysis (CCA) on the participants who had complete outcome and covariates data to provide a comparison with the primary analysis based on imputed data. We examined the MAR assumption that supports the multiple imputation by undertaking sensitivity analyses based on missing not at random (MNAR) assumptions using a practical approximation to the pattern mixture model:[28] (1) imputed total costs were increased by 10%, 20% and 30%; (2) imputed QALYs were reduced by between 10%, 20%, and 30%. To assess the impact of choice of EQ-5D-5L tariff, we took the validated population valuation sets from countries in the southeast Asia area (i.e. Indonesia, Malaysia, Thailand) and the crosswalk functions of the UK and Thailand to calculate utility for comparison.[25, 33-35]

Secondary analyses

The first secondary analysis followed the methods of the primary analysis, extending time horizon to a 12-month period. No discounting was applied as this was not longer than one year. We summarised participants' OOPs in relation to TB treatment, smoking cessation, and healthcare services by arm, at both 6 and 12 months. Productivity losses of participants' sick leave and their companion to treatment, and money spent on any forms of tobacco were also summarised. We have also repeated the analysis by countries following the same methods of the primary analysis above.

RESULTS

Missing data

The results of observed cases are presented in Supplementary file 1. The proportion of missing data at baseline was low (Supplementary file 3 Table S2). The greatest percentage of missing data level was 12% of participants' OOPs for smoking cessation at 6 months follow-up, followed by the same variable at 12 months (10%).

Although missing data did not differentiate between arms, most of the missingness of follow-up variables was significantly associated with country. The missingness of OOP for smoking cessation in months 1-6 was weakly associated with participants' age (Supplementary file 3 Table S3). Using a logistic regression for missingness of follow-up variables on their respective previously observed values (e.g. missingness of costs at six months on costs at baseline), most results were not statistically significant ($p>0.05$), with few exceptions. These results supported the MAR assumption. The imputation number was set to 15.

Primary analysis

The mean costs of smoking cessation and healthcare services in the six months before baseline were PPP US$10.36 (SE PPP US$1.74) in the cytisine arm and PPP US$8.52 (SE PPP US$1.41) in the placebo

For peer review only - http://bmjopen.bmj.com/site/about/guidelines.xhtml

arm. The mean total costs over the six months post-randomisation were PPP US$401.52 (SE PPPUS$8.91) in the cytisine arm and PPP US$334.73 (SE PPP US$5.85) in the placebo arm (Table 2). Costs of additional smoking cessation were negligible in both arms. The mean costs of hospital stay in the cytisine arm were almost twice of those in the placebo arm. The incremental total costs were PPP US$57.74 (95% CI PPP US$49.40 to PPP US$83.36). The mean QALYs were 0.395 (SE 0.002) in the cytisine arm and 0.398 (SE 0.002) in the placebo arm. The incremental QALYs were -0.001 (95% CI -0.004 to 0.002). The majority (78.1%, 3905/5000) of the bootstrapped replicates fell in the north-west quadrant of CEP, indicating a more costly, but less effective intervention (Figure 1 left). The CEAC was not presented as it was a straight line at 0% probability of cost-effectiveness at the WTP range from PPP US$0 to PPP US$1,473 per QALY gained for Bangladesh or PPP US$2,431 per QALY gained for Pakistan.

[Insert Table 2 here]

[Insert Figure 1 here]

Sensitivity analyses

The CCA was performed on 1122 participants in the cytisine arm and 1116 participants in the placebo arm. The results were similar to that of the primary analysis (Table 2 right). The overall majority (91%, 4550/5000) of the bootstrapped replicates fell in the north-west quadrant of CEP (Figure 1 right), indicating a more costly, but less effective intervention. This was consistent with the primary analysis.

Under scenario (1), when the imputed costs were increased by 10%, 20% and 30%, the incremental costs became PPP US$58.32, PPP US$58.91 and PPP US$59.51, respectively. Under scenario (2), when the imputed QALYs were reduced by 10%, 20% and 30%, the incremental QALYs were -0.001, -0.001 and -0.000, respectively. None differed far from the primary analysis results.

Using tariffs derived in different countries or with different approaches, the incremental QALYs between arms varied (Figure 2), but the level of difference was not prominent and the general pattern between arms remained the same.

[Insert Figure 2 here]

Secondary analyses

The addition of the costs in months 7-12 increased the mean total costs over 12 months to PPP US$408.31 (SE PPP US$10.03) in the cytisine arm and PPP US$341.83 (SE PPP US$6.50) in the placebo arm. The incremental costs were PPP US$56.72 (95% CI PPP US$46.58 to PPP US$86.00), similar to those over the six months post randomisation. By contrast, as the time horizon doubled, the QALYs became almost twice as high as over six-month period, which led to a larger difference in mean QALYs between arms. The mean QALYs were 0.808 (SE 0.004) in the cytisine arm and 0.814 (SE 0.004) in the placebo arm. The incremental QALYs were -0.004 (95% CI -0.013 to 0.005). The cytisine arm remained dominated by the placebo arm, with 77% (4007/5000) of the bootstrapped estimates indicating a less effective, but more costly intervention.

Over the 12 months follow-up period, the mean OOPs were PPP US$108.91 (SE PPP US$19.79) in the cytisine arm and PPP US$81.74 (SE PPP US$11.73) in the placebo arm. The main cost driver was OOP for doctor visits in both arms, while in the cytisine arm participants also spent more on hospital stays (Table 3). This pattern was consistent with costs from the public or voluntary healthcare sector's perspective. Productivity losses mostly occurred before and during TB treatment period and

For peer review only - http://bmjopen.bmj.com/site/about/guidelines.xhtml

decreased considerably in the last six months of the trial. The OOP for tobacco products dropped after randomisation in both arms but remained stable throughout the 12 months period post-randomisation, which was consistent with the quit rates observed in both arms.

[Insert Table 3 here]

By country analyses did not lead to different conclusions from the primary analysis. In Bangladesh, the adjusted incremental costs were PPP US$37.06 (95% CI PPP US$28.12 to PPP US$43.85) and the adjusted incremental QALYs were -0.003 (95% CI -0.006 to 0.000), with the cytisine arm remaining dominated by the placebo arm. In Pakistan, the adjusted incremental costs were PPP US$108.46 (95% CI PPP US$69.69 to PPP US$157.88) and the adjusted incremental QALYs were 0.001 (95% CI -0.004 to 0.008). The ICER was calculated at PPP US$108,464 per QALY, which was much higher than the adopted maximum WTP threshold PPP US$2,431 per QALY. The cost-effectiveness plane also shows that cytisine plus BS had 0% of being cost-effective within the adopted WTP threshold range in both countries (Supplementary information 1). However, the breakdown of total costs by country indicated that the higher mean costs of hospital stay in the cytisine arm were mostly contributed by the cytisine arm in Pakistan (PPP US$78.12 vs PPP US$32.70 in placebo arm). While in Bangladesh, the mean costs of hospital stay were PPP US$3.07 (SE PPP US$1.62) in the cytisine arm and PPP US$7.34 (SE PPP US$3.82) in the placebo arm. A further examination also showed possible outliers in the cytisine arm in Pakistan. The improvement in utility from baseline to six months was more manifest in Bangladesh than in Pakistan, regardless of the arms. Detailed results are presented in Supplementary information 1.

DISCUSSION

The intervention cost was PPP US$60.65 (SE PPP US$0.41) per participant in the cytisine arm and PPP US$12.37 (SE PPP US$0.08) per participant in the placebo arm. The difference was mainly attributed to cytisine medication. The incremental total costs at six months post-randomisation were estimated at PPP US$57.74 (95% CI PPP US$49.40 to PPP US$83.36) while the incremental QALYs were estimated at -0.001 (95% CI -0.004 to 0.002). These results indicated that adding cytisine to brief BS for quitting smoking was unlikely to be cost-effective. The sensitivity analyses confirmed the robustness of this conclusion. Extending the time horizon to 12 months did not change the conclusion.

While the observed quit rates were not statistically significantly different between arms, [10] participants' OOP for tobacco products on average dropped by nearly two-thirds after randomisation, indicating a reduction of tobacco consumption. The higher than expected productivity loss, OOPs for doctor visits and TB treatment before baseline might be because participants had experienced some symptoms and sought medical attention before TB was diagnosed. It was unclear, however, why participants in the cytisine arm reported more and longer hospital stays than the placebo arm in Pakistan. Our process evaluation study found some difference in intervention delivery between countries,[36, 37] but we did not find evidence of differential TB treatment outcomes between trial arms in Pakistan, [10] and the same situation was not observed in Bangladesh. This might indicate a potential country-related contextual reason rather than the effect of the intervention, or occurrence by chance. Lack of any clear underlying hypotheses or evidence, subgroup analyses by patient characteristics or deterministic sensitivity analysis of key parameters were not planned and the sample size concerning these factors was likely to be insufficient.

The strength of the study stems from the large sample size and high follow-up rates. Despite limitations of published data availability, patient level measures were collected using a

For peer review only - http://bmjopen.bmj.com/site/about/guidelines.xhtml

comprehensive questionnaire to enable a full cost-utility analysis to be undertaken. However, several limitations could potentially affect the results. Firstly, our estimated costs could be an underestimation. We observed that some health workers discussed smoking cessation during several routine TB consultations and some research assistants delivered the study drug to participants if they had missed day 5 follow-up. TB treatment costs were estimated based on simplified scenarios. Intensive treatments in the case of deterioration, death or retreatment were not considered. Costs of general medication were not included because our unit costs data source for healthcare services did not include them. However, this should not bias the results towards either arm. Secondly, the data source of unit costs of healthcare services was last updated in 2010. Certain changes may not be accounted for by simple inflation. While up-to-date data source was not available at the time of analysis, the results could be updated when it becomes available as the service use was collected in quantities. Thirdly, productivity loss in the case of death was considered zero but if a life-time observation or modelling were undertaken, productivity loss due to premature death should be included. Given the large sample size and few deaths occurred, this was unlikely to affect the conclusions. Last but not least, our sample consisted mostly of males. This reflected the low daily tobacco smoking rate among women in both countries at the time of the trial (0.8% in Bangladesh, 2.0% in Pakistan). [5] There may therefore be challenges in making inferences to female populations.

To our knowledge, this is the first cost-utility study of cytisine as a smoking cessation aid alongside an RCT and one of few for smoking cessation intervention in LMICs. A systematic review published in 2019 identified eight placebo-controlled trials and one non-inferiority trial (using nicotine replacement therapies) that used cytisine for smoking cessation, all of which were among smokers in general population and only one was conducted in LMICs.[8] Although cytisine has been identified as affordable globally [38] its cost-effectiveness in smoking cessation was based on modelled economic evaluation not empirical evidence.[39] Our study illustrated that though less costly than other cessation aids, cytisine did not show sufficient effects to be considered cost-effective.

Our findings do not support the cost-effectiveness of adding cytisine to BS for smokers who are newly diagnosed with pulmonary TB. In the absence of more effective smoking cessation aid, future studies should explore the cost-effectiveness of non-pharmacological cessation interventions in LMICs, given the relatively lower costs of labour, and possible impact of smoking-related comorbidities on quality of life in the TB population.

FUNDING

This work was supported by the European Union's Horizon 2020 research and innovation programme, under Grant Agreement No. 680995. The European Commission is not responsible for any of the content of this article. The manufacturer, Aflofarm, supplied trial medication at no cost.

PATIENT AND PUBLIC INVOLVEMENT

Patient groups were consulted on the intervention materials for their lucidness during the intervention development stage. No other patient and public involvement occurred in the study process.

For peer review only - http://bmjopen.bmj.com/site/about/guidelines.xhtml

CONFLICT OF INTERESTS

Kamran Siddiqi received a research grant from Pfizer (2015-2017) to study the effects of varenicline (a smoking cessation medication) on waterpipe smoking cessation. Daniel Kotz received an unrestricted grant from Pfizer in 2009 for an investigator-initiated trial on the effectiveness of practice nurse counselling and varenicline for smoking cessation in primary care (Dutch Trial Register NTR3067; DOI: 10.1111/add.13927). The medication for the trial were provided by Aflofarm free of charge. Aziz Sheikh is supported by Health Data Research UK's BREATHE Hub.

DATA SHARING STATEMENT

Access to partial anonymised datasets from the study can be provided upon request to KS (Chief Investigator) through corresponding author of this manuscript. Statistical code is available on GitHub (Clearice84/TBTobacco: Health economic analysis STATA code for TB&Tobacco trial (github.com)).

AUTHORS' CONTRIBUTION

JL conducted the cost-effectiveness analysis and drafted the manuscript under the supervision of SP. SP also contributed to the analysis design. AKe contributed to data management and statistical analysis, including some clinical measures used in this manuscript. OD and RG contributed to study design, conduct and interpretation of findings. AR and AMM managed the study and contributed to interpretation of findings. RH, DB, RF, AK, RZ and SM conducted the study in Bangladesh/Pakistan, collected and managed the data in countries and provided critical inputs to data analysis and interpretation. DK, EK, MB and HE provided insights to study design on aspects of behavioural support implementation, evaluation of its delivery and interpretation of findings. AS provided critical oversight to study design, trial conduct, interpretation of findings and discussion. KS conceptualised the study, contributed to the study design, conduct, and interpretation of findings.

All authors provided critical revisions and approved the final manuscript.

Figure 1: Cost-effectiveness plane of primary and complete case analyses at six months post-randomisation (dashed purple line as WTP for Pakistan, dotted green line as WTP for Bangladesh)

Figure 2: Comparison of adjusted incremental QALYs over six months post-randomisation derived from different methods

For peer review only - http://bmjopen.bmj.com/site/about/guidelines.xhtml

REFERENCES

1. World Health Organization. Global tuberculosis report 2021. Geneva; 2021 14 October. https://www.who.int/publications/i/item/9789240037021.
2. TB country, regional and global profiles [Internet]. World Health Organization. 2021 [cited 01/12/2021]. Available from: https://worldhealthorg.shinyapps.io/tb_profiles/.
3. World Health Organization. WHO global report on trends in prevalence of tobacco use 2000-2025. Geneva; 2021 16 November. https://www.who.int/publications/i/item/9789240039322.
4. Chaves Torres NM, Quijano Rodriguez JJ, Porras Andrade PS, et al. Factors predictive of the success of tuberculosis treatment: A systematic review with meta-analysis. *PloS one*. 2019;14(12):e0226507. https://www.ncbi.nlm.nih.gov/pubmed/31881023.
5. World Health Organization (WHO). Tobacco control profiles - countries, territories, and areas: Tobacco Free Initiative; 2019 [cited 2020 6th July]. https://www.who.int/tobacco/surveillance/policy/country_profile/en/#P.
6. Lawson PJ, Flocke SA. Teachable moments for health behavior change: a concept analysis. *Patient Educ Couns*. 2009;76(1):25-30. https://www.ncbi.nlm.nih.gov/pubmed/19110395.
7. Elsey H, Warsi S. Report of TB and Tobacco Project WP1: tobacco cessation intervention development [Internet]2017 01/12/2021 [cited 2021 1 December]. https://tbandtobacco.org/wp-content/uploads/sites/26/2019/05/WP1-Report-of-intervention-development.pdf.
8. Tutka P, Vinnikov D, Courtney RJ, et al. Cytisine for nicotine addiction treatment: a review of pharmacology, therapeutics and an update of clinical trial evidence for smoking cessation. *Addiction*. 2019;114(11):1951-69. https://www.ncbi.nlm.nih.gov/pubmed/31240783.
9. Dogar O, Barua D, Boeckmann M, et al. The safety, effectiveness and cost-effectiveness of cytisine in achieving six-month continuous smoking abstinence in tuberculosis patients-protocol for a double-blind, placebo-controlled randomized trial. *Addiction*. 2018. https://www.ncbi.nlm.nih.gov/pubmed/29676824.
10. Dogar O, Keding A, Gabe R, et al. Cytisine for smoking cessation in patients with tuberculosis: a multicentre, randomised, double-blind, placebo-controlled phase 3 trial. *Lancet Glob Health*. 2020;8(11):e1408-e17. https://www.ncbi.nlm.nih.gov/pubmed/33069301.
11. Prices, Consumer Price Index, All items, Index [Internet]. International Monetary Fund (IMF). 2019 [cited 08/05/2019]. Available from: http://data.imf.org/regular.aspx?key=61545861 Archived at http://www.webcitation.org/78DlAn9Cu.
12. DataBank World Development Indicators [Internet]. 2019 [cited 26/06/2019]. Available from: https://databank.worldbank.org/reports.aspx?source=2&series=PA.NUS.PPP&country=PAK#.
13. Global Tuberculosis report 2018 - TB data [Internet]. WHO. 2018 [cited 20/05/2019]. Available from: https://www.who.int/tb/country/data/download/en/ Archived at: http://www.webcitation.org/78VxUbGR1.
14. Gomez GB, Dowdy DW, Bastos ML, et al. Cost and cost-effectiveness of tuberculosis treatment shortening: a model-based analysis. *BMC Infect Dis*. 2016;16(1):726. https://www.ncbi.nlm.nih.gov/pubmed/27905897.
15. National Currency per U.S. Dollar, period average [Internet]. International Monetary Fund (IMF). 2019 [cited 08/05/2019]. Available from: http://data.imf.org/regular.aspx?key=61545862 Archived at http://www.webcitation.org/78DkMANRB.
16. World Health Organization. Tobacco control country profile Pakistan. Initiative TF; 2017. https://www.who.int/tobacco/surveillance/policy/country_profile/pak.pdf?ua=1.
17. World Health Organization. Tobacco control country profile Bangladesh. Initiative TF; 2017. https://www.who.int/tobacco/surveillance/policy/country_profile/bgd.pdf?ua=1.
18. National Institute for Health and Care Excellence. Putting NICE guidance into practice - Resource impact report: Stop smoking interventions and services (NG92). 2018 March. Available at: https://www.nice.org.uk/guidance/ng92/resources/resource-impact-report-pdf-4788823645 Archived at: http://www.webcitation.org/766hqbhTb.

For peer review only - http://bmjopen.bmj.com/site/about/guidelines.xhtml

19. Curtis LA, Burns A. Unit Costs of Health and Social Care 2018. Project report. Kent: University of Kent; 2018 14th January. Available at: https://kar.kent.ac.uk/70995/1/Unit%20Costs%202018%20-%20FINAL%20with%20bookmarks%20and%20covers.pdf Archived at: http://www.webcitation.org/75n7YG9xI.
20. Bangladesh Bureau of Statistics. Report on Labour Force Survey (LFS) 2016-17. Statistics, Informatics Division MoP; 2018 January. https://www.ilo.org/surveydata/index.php/catalog/2047/download/16818.
21. Pakistan Bureau of Statistics. Labour Force Survey 2017-18. Ministry of Statistics GoP; 2018 December. http://www.pbs.gov.pk/sites/default/files//Labour%20Force/publications/lfs2017_18/Annual%20Report%20of%20LFS%202017-18.pdf.
22. Economic Analysis Evaluation Team. Country-specific inpatient and outpatient estimates in 2010 currency. World Health Organisation, Department of Health Systems G, Financing; 2010. https://www.who.int/choice/cost-effectiveness/inputs/country_inpatient_outpatient_2010.pdf?ua=1.
23. International Labour Office. International Standard Classification of Occupations: ISCO-08. Geneva: ILO; 2012.
24. EuroQol Research Foundation. EQ-5D-5L User Guide: Basic information on how to use the EQ-5D-5L instrument (version 3.0)2019 17/06/2020. Available at: https://euroqol.org/publications/user-guides/.
25. van Hout B, Janssen MF, Feng YS, et al. Interim scoring for the EQ-5D-5L: mapping the EQ-5D-5L to EQ-5D-3L value sets. *Value Health*. 2012;15(5):708-15. https://www.ncbi.nlm.nih.gov/pubmed/22867780.
26. IMF DataMapper - GDP per capita, current prices (Purchasing power parity; international dollars per capita) [Internet]. International Monetary Fund (IMF). 2018 [cited 4th April 2019]. Available from: https://www.imf.org/external/datamapper/PPPPC@WEO/BGD/PAK/ZWE/GBR/THA/MYS/IDN/SEQ?year=2018.
27. Richardson G, Manca A. Calculation of quality adjusted life years in the published literature: a review of methodology and transparency. *Health Econ*. 2004;13(12):1203-10. https://www.ncbi.nlm.nih.gov/pubmed/15386669.
28. Faria R, Gomes M, Epstein D, et al. A guide to handling missing data in cost-effectiveness analysis conducted within randomised controlled trials. *PharmacoEconomics*. 2014;32(12):1157-70. https://www.ncbi.nlm.nih.gov/pubmed/25069632.
29. Rubin DB. Statistical Matching Using File Concatenation with Adjusted Weights and Multiple Imputations. *J Bus Econ Stat*. 1986;4(1):87-94. <Go to ISI>://WOS:A1986AXF5400013.
30. White IR, Royston P, Wood AM. Multiple imputation using chained equations: Issues and guidance for practice. *Stat Med*. 2011;30(4):377-99. https://www.ncbi.nlm.nih.gov/pubmed/21225900.
31. Woods B, Revill P, Sculpher M, et al. Country-Level Cost-Effectiveness Thresholds: Initial Estimates and the Need for Further Research. *Value Health*. 2016;19(8):929-35. https://www.ncbi.nlm.nih.gov/pubmed/27987642.
32. Fenwick E, Claxton K, Sculpher M. Representing uncertainty: the role of cost-effectiveness acceptability curves. *Health Econ*. 2001;10(8):779-87. https://www.ncbi.nlm.nih.gov/pubmed/11747057.
33. Pattanaphesaj J, Thavorncharoensap M, Ramos-Goni JM, et al. The EQ-5D-5L Valuation study in Thailand. *Expert Rev Pharmacoecon Outcomes Res*. 2018;18(5):551-8. https://www.ncbi.nlm.nih.gov/pubmed/29958008.
34. Purba FD, Hunfeld JAM, Iskandarsyah A, et al. The Indonesian EQ-5D-5L Value Set. *PharmacoEconomics*. 2017;35(11):1153-65. https://www.ncbi.nlm.nih.gov/pubmed/28695543.

For peer review only - http://bmjopen.bmj.com/site/about/guidelines.xhtml

35. Shafie AA, Vasan Thakumar A, Lim CJ, et al. EQ-5D-5L Valuation for the Malaysian Population. *PharmacoEconomics*. 2019;37(5):715-25. https://www.ncbi.nlm.nih.gov/pubmed/30535779.
36. Boeckmann M, Warsi S, Noor M, et al. Health worker and patient views on implementation of smoking cessation in routine tuberculosis care. *NPJ Prim Care Respir Med*. 2019;29(1):34. https://www.ncbi.nlm.nih.gov/pubmed/31481678.
37. Boeckmann M, Dogar O, Saeed S, et al. Measuring fidelity to delivery of a new smoking cessation intervention integrated into routine tuberculosis care in Pakistan and Bangladesh: Contextual differences and opportunities. *Tob Induc Dis*. 2021;19:24. https://www.ncbi.nlm.nih.gov/pubmed/33841063.
38. West R, Raw M, McNeill A, et al. Health-care interventions to promote and assist tobacco cessation: a review of efficacy, effectiveness and affordability for use in national guideline development. *Addiction*. 2015;110(9):1388-403. https://www.ncbi.nlm.nih.gov/pubmed/26031929.
39. Leaviss J, Sullivan W, Ren S, et al. What is the clinical effectiveness and cost-effectiveness of cytisine compared with varenicline for smoking cessation? A systematic review and economic evaluation. *Health Technol Assess*. 2014;18(33):1-120. https://www.ncbi.nlm.nih.gov/pubmed/24831822.

For peer review only

Table 1 Unit costs of TB treatment, smoking cessation services and healthcare services

**Cost items**	**Unit Cost (PPP US$, 2017/18)**		**Sources**
	**Bangladesh**	**Pakistan**	
**TB treatment**			
First-line treatment, intensive phase, including drugs	54.21 per month	108.40 per month	[12-15]
First-line treatment, continuation phase, including drugs	31.62 per month	63.24 per month	
**Smoking cessation services**			
Help or advice from public/government clinic/hospital	0.68/use	0.89/use	[12, 19-21]
Group or single counselling session at public/voluntary clinic	0.94/session	1.26/session	[12, 18, 20, 21]
**General healthcare services**			
Doctor visit	4.60/visit	6.83/visit	[11, 12, 22]
Hospital inpatient	19.06/bed-day	33.14/bed-day	[11, 12, 22]

Table 2 Results of primary and complete cases analyses at six months post-randomisation

	**Primary analysis**		**Complete case analysis**	
	**Cytisine (n=1239)**	**Placebo (n=1233)**	**Cytisine (n=1122)**	**Placebo (n=1116)**
Costs (PPP US$)	Mean (SE)		Mean (SD)	
Intervention	60.65 (0.41)	12.37 (0.08)	61.25 (13.83)	12.15 (2.69)
TB treatment	305.15 (3.36)	301.83 (3.36)	306.53 (109.96)	301.36 (108.09)
Doctor visit	3.36 (0.37)	3.10 (0.31)	3.47 (13.17)	3.14 (10.58)
Hospital stay	31.91 (7.73)	16.98 (4.41)	33.08 (275.18)	17.26 (151.58)
Smoking cessation	0.46 (0.03)	0.45 (0.03)	0.49 (1.19)	0.49 (1.13)
Overall total for six months	*401.52 (8.91)*	*334.73 (5.85)*	*404.82 (311.99)*	*334.39 (196.52)*
**PPP US$, Mean (95% CI)**				
**Adjusted incremental costs**	**57.74 (49.40 to 83.36)**		**59.49 (51.95 to 89.30)**	
	Mean (SE)		Mean (SD)	
QALYs over six months	*0.395 (0.002)*	*0.398 (0.002)*	*0.401 (0.041)*	*0.403 (0.039)*
**Mean (95% CI)**				
**Adjusted incremental QALYs**	**-0.001 (-0.004 to 0.002)**		**-0.001 (-0.003 to 0.000)**	
**ICER**	**Cytisine dominated by placebo (uncertainty see Figure 1 left)**		**Cytisine dominated by placebo (uncertainty see Figure 1 right)**	

For peer review only - http://bmjopen.bmj.com/site/about/guidelines.xhtml

Table 3 Mean Out-of-pocket payments for health-related services, productivity loss and payments for tobacco products at three time points, by arm

**PPP US$ Mean (SE)**	**Cytisine (n=1239)**	**Placebo (n=1233)**
**Six months before baseline**		
OOPs for health-related services	84.90 (7.91)	86.70 (6.80)
TB treatment	15.60 (1.69)	19.71 (3.42)
Doctor visit	62.29 (6.90)	63.96 (5.67)
Hospital stay	6.97 (2.87)	3.02 (0.80)
Smoking cessation	0.04 (0.02)	0.01 (0.01)
Productivity loss	34.01 (2.14)	30.41 (1.81)
OOPs for tobacco products	1.79 (0.14)	1.64 (0.07)
**Months 1 – 6**		
OOPs for health-related services	69.70 (10.62)	51.08 (9.32)
TB treatment	22.16 (2.51)	16.24 (1.30)
Doctor visit	29.49 (7.52)	22.65 (6.08)
Hospital stay	17.65 (5.90)	11.89 (6.53)
Smoking cessation	0.40 (0.09)	0.30 (0.06)
Productivity loss	48.83 (3.00)	43.52 (3.14)
OOPs for tobacco products	0.51 (0.03)	0.50 (0.03)
**Months 7 – 12**		
OOPs for health-related services	39.21 (16.11)	30.66 (6.72)
TB treatment	5.03 (1.43)	4.55 (0.92)
Doctor visit	13.05 (2.41)	20.42 (5.22)
Hospital stay	21.08 (15.80)	5.64 (2.89)
Smoking cessation	0.04 (0.02)	0.05 (0.02)
Productivity loss	6.06 (0.58)	8.32 (0.97)
OOPs for tobacco products	0.61 (0.03)	0.58 (0.02)

For peer review only - http://bmjopen.bmj.com/site/about/guidelines.xhtml

For peer review only

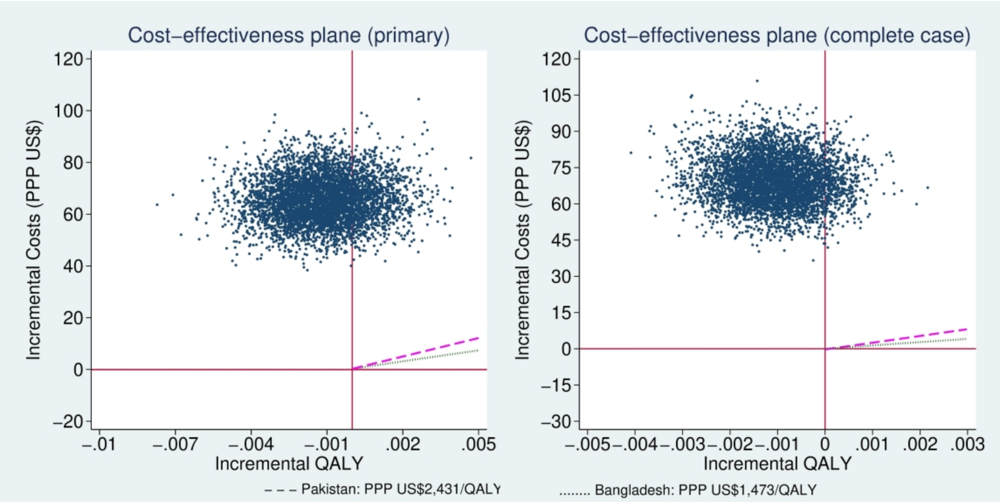

Cost-effectiveness plane of primary and complete case analyses at six months post-randomisation

254x127mm (300 x 300 DPI)

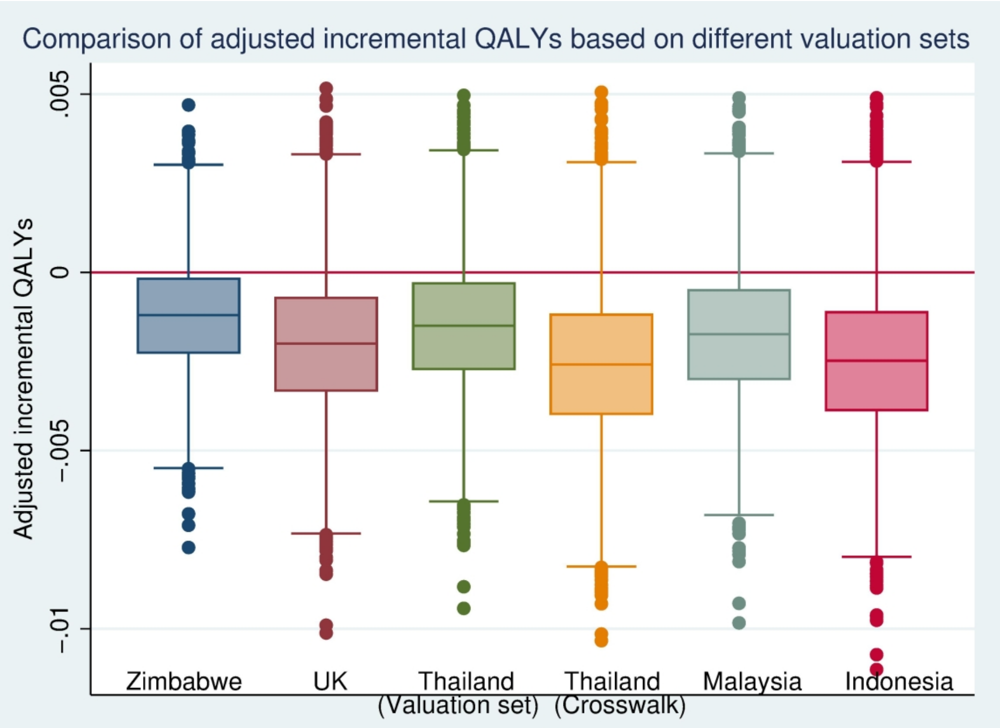

Comparison of adjusted incremental QALYs over six months post-randomisation derived from different methods

139x101mm (600 x 600 DPI)

Detailed methods and results of secondary analyses

Methods

Cytisine dosage schedule

The standard regimen for cytisine (Desmoxan, Aflofarm) was a 25-day course with 1.5mg hard capsules for oral administration, with six per day on days 1-3, five per day on days 4-12, four per day on days 13-16, three per day on days 17-20, two per day on days 21-24 and one on the last day.

Intervention costs

Training for the delivery of brief behavioural support was given to TB health workers before the trial began. In Bangladesh, it was a one-day training programme with a one-day refresher training and the total cost was estimated to be €4499 in 2017. In Pakistan, this consisted of a two-day training programme for DOTS facilitators and the total cost was estimated at €2324 in 2016.

In Bangladesh, the average monthly salary of a TB health worker (local salary grades G-11 to G-13) was, and average working hours per week was 48 hours.[1] In Pakistan, the average monthly salary of a TB health worker was PPP US$921.50 and average working hours per week was 47.4 hours.[2] We assumed a 30-day month as 4.3 weeks. The estimated hourly wage was therefore PPP US$3.17 in Bangladesh and PPP US$4.54 in Pakistan. The cost of BS was PPP US$0.52 for the first session and PPP US$0.26 for the second session in Bangladesh and PPP US$0.75 and PPP US$0.38 in Pakistan.

TB treatment costs

The standard treatment for pulmonary TB consisted of a two-month intensive phase and a four-month continuation phase. We extracted the overall costs of a six-month TB treatment for the two countries from the World Health Organization (WHO) TB database[3] and applied a ratio of costs of the two phases, based on a TB treatment modelling study,[4] to produce an estimate of monthly cost of intensive phase and continuation phase respectively. They were then converted to PPP US$.[5 6] The TB treatment costs were then estimated based on the participants' treatment progression on their TB registry cards.

Smoking cessation costs outside of the trial

Due to the limited smoking cessation services in the two countries,[7 8] we made assumptions on duration, based on usual practice in the UK:[9 10] 10-minute brief intervention with professionals (physician or professional nurse) for help/advice from a public/government clinic/hospital; one hour group session of 15 people or 30-minute individual session led by medical technicians/auxiliary nurses for counselling sessions in public/voluntary hospital. The ratio of group and individual sessions was assumed to be 1:1. The average hourly wage was PPP US$4.14 for "professionals" and PPP US$3.33 for "technicians and associate professionals" in Bangladesh, and PPP US$5.29 for "professionals" and PPP US$4.51 for "technicians and associate professionals" in Pakistan.[1 2 5]

General healthcare services costs

Participants' visits to a public/voluntary doctor and length of stay in a public hospital in the previous six months were collected by self-report at baseline, 6- and 12-month follow-ups. The unit costs of these services were extracted from the WHO country specific in- and out-patient costs, inflated to 2018 and converted to PPP US$.[5 11 12] The unit cost of hospital inpatient stay was the average of all hospital levels and the unit cost of a visit to doctor was the average of all settings for outpatient. These costs did not include drugs.

For peer review only

Out-of-pocket payments (OOPs)

Participants' spending related to following items were collected: TB treatment, public/voluntary doctor and hospital visits, and private doctor and hospital visits, including travel, smoking cessation services in public/voluntary facilities and private settings, purchasing Nicotine Replacement Therapy (NRT) or e-cigarette refills, purchasing other traditional medicine for quitting, and purchasing tobacco products.

Results

Costs

Mean training costs were PPP US$10.94 (SD PPP US$2.09) per participant in the cytisine arm and PPP US$10.92 (SD PPP US$2.09) per participant in the placebo arm. Mean cost of the information leaflet was PPP US$0.76 (SD PPP US$0.75) in the cytisine arm and PPP US$0.75 (SD PPP US$0.75) in the placebo arm. Mean cost of BS was PPP US$0.68 (SD PPP US$0.36) among 1233 participants in the cytisine arm and PPP US$0.70 (SD US$0.36) among 1226 participants in the placebo arm. Mean cost of cytisine was PPP US$48.27 (SD PPP US$12.54) while the cost of placebo was assumed at zero.

Mean costs of TB treatment were estimated to be PPP US$307.39 (SD PPP US$110.25) in the cytisine arm and PPP US$302.45 (SD PPP US$108.53) in the placebo arm, excluding 102 (8.2%) participants in the cytisine arm and 103 (8.4%) in the placebo arm who did not have information from TB cards at six-month follow-up (Table 1). The use of smoking cessation support was reported by a small group of participants in both arms. Mean costs of public/voluntary smoking cessation services were low in both arms throughout the 12 months period. Most participants reported neither visiting a doctor other than for their TB treatment nor being admitted to hospital for any reason. While mean costs of doctor visits were similar between respondents in both arms throughout the trial period, mean costs of hospital stay in the cytisine arm were nearly twice as high as in the placebo arm in months 1-6.

Out-of-pocket payments

The respondents reported an increase of spending on smoking cessation in months 1-6 compared to close to none before and after, corresponding with the intervention delivery and TB treatment period. Mean spending on tobacco was lower during the trial period than before among respondents. However, in comparison with the spending on smoking cessation, the spending on tobacco was consistently higher. The OOPs for healthcare services, including travel, loosely followed the same pattern of the costs of the services (Table 1).

Table 1 Mean (SD) costs and OOPs of TB treatment, additional smoking cessation services and general healthcare services, and OOPs on tobacco products, by arm

	**Cytisine (n=1239)**		**Placebo (n=1233)**	
	**n**	**Mean (SD) PPP US$**	**n**	**Mean (SD) PPP US$**
**TB treatment costs**				
TB registry	1137	307.39 (110.25)	1130	302.45 (108.53)
**Additional smoking cessation costs**				
Six months before baseline	1239	0.00 (0.10)	1233	0.00 (0.09)
Months 1-6	1174	0.47 (1.17)	1164	0.47 (1.11)
Months 7-12	1134	0.22 (0.75)	1144	0.21 (0.77)
**Doctor visit costs**				
Six months before baseline	1239	3.26 (14.27)	1232	3.48 (23.44)
Months 1-6	1176	3.39 (12.96)	1166	3.04 (10.39)

Months 7-12	1148	1.27 (4.73)	1157	1.12 (4.58)
**Hospital stay costs**				
Six months before baseline	1237	6.77 (57.79)	1231	4.84 (43.25)
Months 1-6	1175	31.58 (268.99)	1166	16.52 (148.33)
Months 7-12	1148	5.01 (80.30)	1157	5.87 (94.86)
**Additional smoking cessation OOPs**				
Six months before baseline	1236	0.04 (0.75)	1230	0.00 (0.09)
Months 1-6	1091	0.34 (2.72)	1080	0.28 (1.95)
Months 7-12	1110	0.05 (0.61)	1115	0.05 (0.56)
**Tobacco OOPs**				
Six months before baseline	1229	1.79 (5.05)	1224	1.64 (2.35)
Months 1-6	1177	0.50 (1.03)	1166	0.48 (0.91)
Months 7-12	1148	0.58 (0.92)	1157	0.57 (0.75)
**TB treatment OOPs**				
Six months before baseline	1238	15.45 (59.42)	1233	19.71 (119.96)
Months 1-6	1174	22.00 (85.28)	1164	15.77 (42.34)
Months 7-12	1148	5.03 (48.72)	1156	4.36 (30.50)
**Doctor visit OOPs**				
Six months before baseline	1233	61.53 (243.17)	1227	63.21 (199.10)
Months 1-6	1173	27.49 (238.38)	1158	22.07 (216.35)
Months 7-12	1148	13.28 (84.58)	1157	19.07 (162.71)
**Hospital stay OOPs**				
Six months before baseline	1237	6.91 (101.21)	1231	3.01 (28.19)
Months 1-6	1173	16.65 (200.58)	1164	11.72 (220.92)
Months 7-12	1148	17.20 (460.84)	1157	5.65 (99.21)

Productivity loss

Among the respondents, while the mean productivity loss peaked in months 1-6 as expected, it was higher than expected in the six months before baseline, most prominently reflected by productivity loss due to participants' sick leave (Table 2). This might correspond with productivity loss due to companion to TB clinic in the six months before baseline, which was consistent with participants' OOPs for TB clinic during the same period.

Table 2 Mean (SD) productivity loss of companion to TB clinic, doctor, and participants' sick leave, by arm

	**Cytisine (n=1239)**		**Placebo (n=1233)**	
	**n**	**Mean (SD) PPP US$**	**n**	**Mean (SD) PPP US$**
**Companion to TB clinic**				
Six months before baseline	*1232*	*4.62 (9.01)*	*1228*	*4.48 (7.73)*
Month 1 – 6	*1134*	*13.45 (21.55)*	*1127*	*12.43 (19.86)*
Month 7 – 12	*1145*	*2.01 (7.40)*	*1152*	*2.33 (7.19)*
**Companion to doctor**				
Six months before baseline	*1203*	*2.10 (9.15)*	*1196*	*1.87 (6.05)*
Month 1 – 6	*1126*	*3.35 (13.22)*	*1116*	*2.65 (8.44)*
Month 7 – 12	*1143*	*0.37 (2.82)*	*1151*	*0.56 (4.82)*
**Sick leave**				
Six months before baseline	*1230*	*27.14 (73.17)*	*1227*	*23.82 (61.12)*
Month 1 – 6	*1194*	*31.98 (100.27)*	*1171*	*28.52 (107.49)*

For peer review only

	Cytisine (n=1239)		Placebo (n=1233)	
	**n**	**Mean (SD) PPP US$**	**n**	**Mean (SD) PPP US$**
Month 7 – 12	*1163*	*3.62 (18.24)*	*1160*	*5.14 (28.21)*

Quality-adjusted life years

In the EQ-5D-5L descriptive system, the domains with least proportion of respondents scoring no problem were Pain/Discomfort and Anxiety/Depression at all three time points although the proportion increased after baseline (Table 3).

Table 3 Number and percentage of respondents scoring five levels of each domain of EQ-5D-5L, by arm and time point

Domains	Mobility		Self-care		Usual activities		Pain/ Discomfort		Anxiety/ Depression	
	Cytisine	Placebo	Cytisine	Placebo	Cytisine	Placebo	Cytisine	Placebo	Cytisine	Placebo
Baseline										
1	731	746	985	993	655	654	413	426	407	411
	59%	61%	79%	81%	53%	53%	33%	35%	33%	33%
2	315	291	190	163	380	373	447	462	453	463
	25%	24%	15%	13%	31%	30%	36%	38%	37%	38%
3	140	143	49	58	133	146	250	227	232	231
	11%	12%	4%	5%	11%	12%	20%	18%	19%	19%
4	50	51	12	16	55	52	114	104	112	98
	4%	4%	1%	1%	4%	4%	9%	8%	9%	8%
5	3	2	3	1	14	8	14	13	33	29
	0%	0%	0%	0%	1%	1%	1%	1%	3%	2%
Total	1239	1233	1239	1231	1237	1233	1238	1232	1237	1232
Six months										
1	985	992	1077	1078	945	960	753	778	818	829
	86%	88%	94%	95%	83%	85%	66%	69%	72%	73%
2	119	116	56	44	171	147	364	325	287	260
	10%	10%	5%	4%	15%	13%	32%	29%	25%	23%
3	25	13	7	8	19	18	19	20	28	29
	2%	1%	1%	1%	2%	2%	2%	2%	2%	3%
4	12	10	2	2	5	5	6	7	8	12
	1%	1%	0%	0%	0%	0%	1%	1%	1%	1%
5	2	1	1	0	3	1	1	1	2	1
	0%	0%	0%	0%	0%	0%	0%	0%	0%	0%
Total	1143	1132	1143	1132	1143	1131	1143	1131	1143	1131
12 months										
1	994	1020	1059	1082	968	996	755	780	826	833
	90%	91%	96%	97%	88%	89%	69%	70%	75%	75%
2	86	75	33	24	115	101	299	284	226	238
	8%	7%	3%	2%	10%	9%	27%	26%	21%	21%
3	11	12	6	2	12	10	35	34	33	28

For peer review only

	1%	1%	1%	0%	1%	1%	3%	3%	3%	3%
4	8	6	3	4	4	5	9	13	8	13
	1%	1%	0%	0%	0%	0%	1%	1%	1%	1%
5	3	2	1	1	3	1	3	2	4	1
	0%	0%	0%	0%	0%	0%	0%	0%	0%	0%
Total	1102	1115	1102	1113	1102	1113	1101	1113	1097	1113

Levels for each domain: 1=no problem, 2=slight problem, 3=moderate problem, 4=severe problem, 5=extreme problem/inability

Table 4 shows mean EQ-5D-5L utility and VAS among observed cases at baseline, 6 and 12 months follow-ups and QALYs over 6 and 12 months period. Mean utility in the cytisine arm appeared to be consistently lower than in the placebo arm at all timepoints though the difference was small. The mean QALYs were therefore lower in the cytisine arm than in the placebo arm. However, it should be noted, only those who had data on all relevant timepoints were included in calculating QALYs. The EQ-5D VAS showed a similar pattern where both arms began at similar level but in the cytisine arm, the observed cases scored slightly lower than those in the placebo arm in the follow-ups.

Table 4 Mean (SD) EQ-5D-5L utility, EQ-5D VAS and QALYs, by arm

	**Cytisine (n=1239)**		**Placebo (n=1233)**	
	**n**	**Mean (SD)**	**n**	**Mean (SD)**
**Utility**				
Baseline	1234	0.754 (0.133)	1229	0.759 (0.130)
6 months	1179	0.825 (0.165)	1164	0.831 (0.161)
12 months	1144	0.822 (0.189)	1149	0.829 (0.176)
**QALYs**				
Over 6 months	1174	0.394 (0.056)	1160	0.397 (0.054)
Over 12 months	1129	0.805 (0.134)	1122	0.810 (0.128)
**VAS**				
Baseline	1239	53.5 (15.4)	1233	53.5 (16.0)
6 months	1179	80.5 (20.3)	1165	81.3 (19.8)
12 months	1150	84.0 (21.8)	1156	84.7 (20.7)

Cost-utility analysis by country

The mean costs of smoking cessation and healthcare services in the six months before baseline were PPP US$18.33 (SE PPP US$3.65) in Pakistan and PPP US$5.40 (SE PPP US$1.65) in Bangladesh in the cytisine arm. In the placebo arm, the mean costs of these two types of services were PPP US$16.35 (SE PPP US$3.55) in Pakistan and PPP US$3.72 (SE PPP US$0.55) in Bangladesh.

The mean costs of intervention were PPP US$74.37 (SE PPP US$0.68) in the cytisine arm and PPP US$15.84 (SE PPP US$0.03) in the placebo arm in Pakistan. The mean costs of intervention were PPP US$52.10 (SE PPP US$0.13) in the cytisine arm and PPP US$10.23 (SE PPP US$0.00) in the placebo arm in Bangladesh.

The mean costs of TB treatment in the two arms were on a similar level within each country, over PPP US$400 in Pakistan and over PPP US$200 in Bangladesh. The mean costs of doctor visits were very similar between arms in Bangladesh, but they were slightly higher in the cytisine arm in Pakistan (PPP US$3.17 vs PPP US$2.66). The most prominent difference was in the mean costs of

hospital stay. In Pakistan, the mean costs of hospital stay were considerably higher in the cytisine arm (PPP US$78.12 [SE PPP US$19.80]) than in the placebo arm (PPP US$32.70 [SE PPP US$9.76]). On the contrary, in Bangladesh, the mean costs of hospital stay in the placebo arm (PPP US$7.35 [SE PPP US$3.82]) were over twice as high as in the cytisine arm (PPP US$3.07 [SE PPP US$1.62]). The mean costs of smoking cessation services were not different between arms within each country. However, there were nearly null costs incurred in Pakistan.

Upon further investigation, there were more participants had hospital stays in Pakistan than in Bangladesh in both arms. Among participants who incurred hospital stay costs over the six months post-randomisation, not only did the cytisine arm in Pakistan have more participants admitted to hospital but also showed a few potential outliers (Figure 1). This was in contrast with the placebo arm in Pakistan and both arms in Bangladesh.

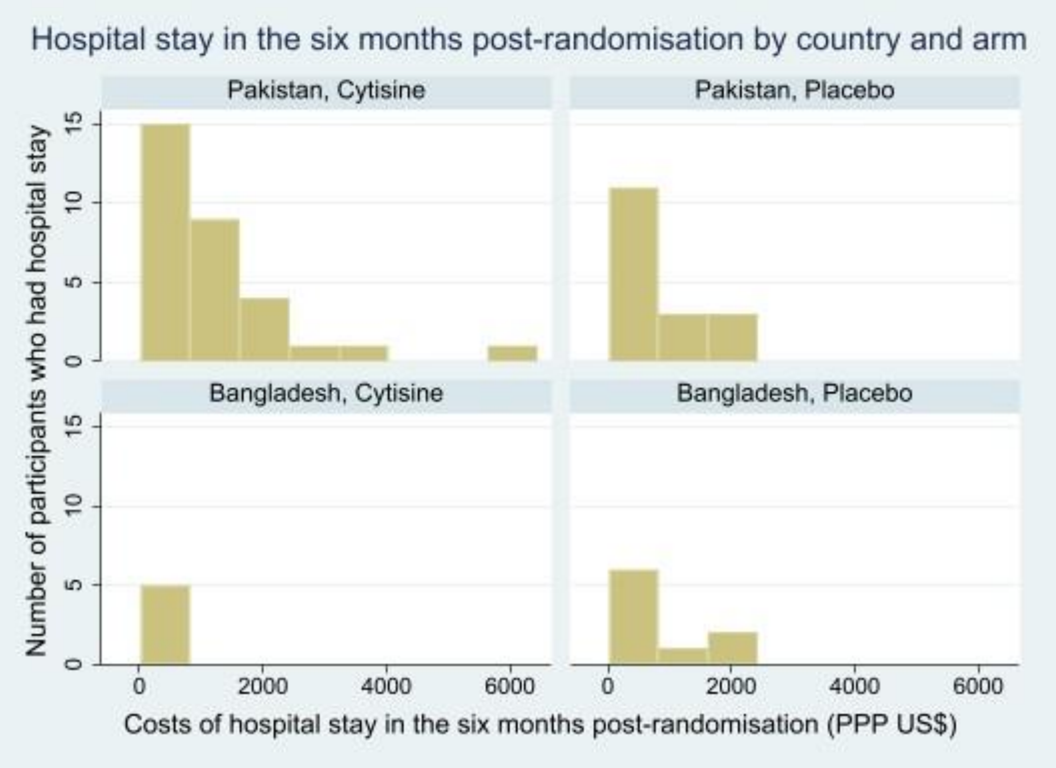

Figure 1 Distribution of costs of hospital stay among those who incurred this cost, by country and arm

Whilst the mean utility was higher in Pakistan than in Bangladesh, the mean utility in both arms showed a relatively gradual and small increase from baseline to six months (Figure 2). In contrast, the mean utility at baseline was much lower in Bangladesh than in Pakistan but it increased more sharply to a similar level in the cytisine arm and a higher level in the placebo arm.

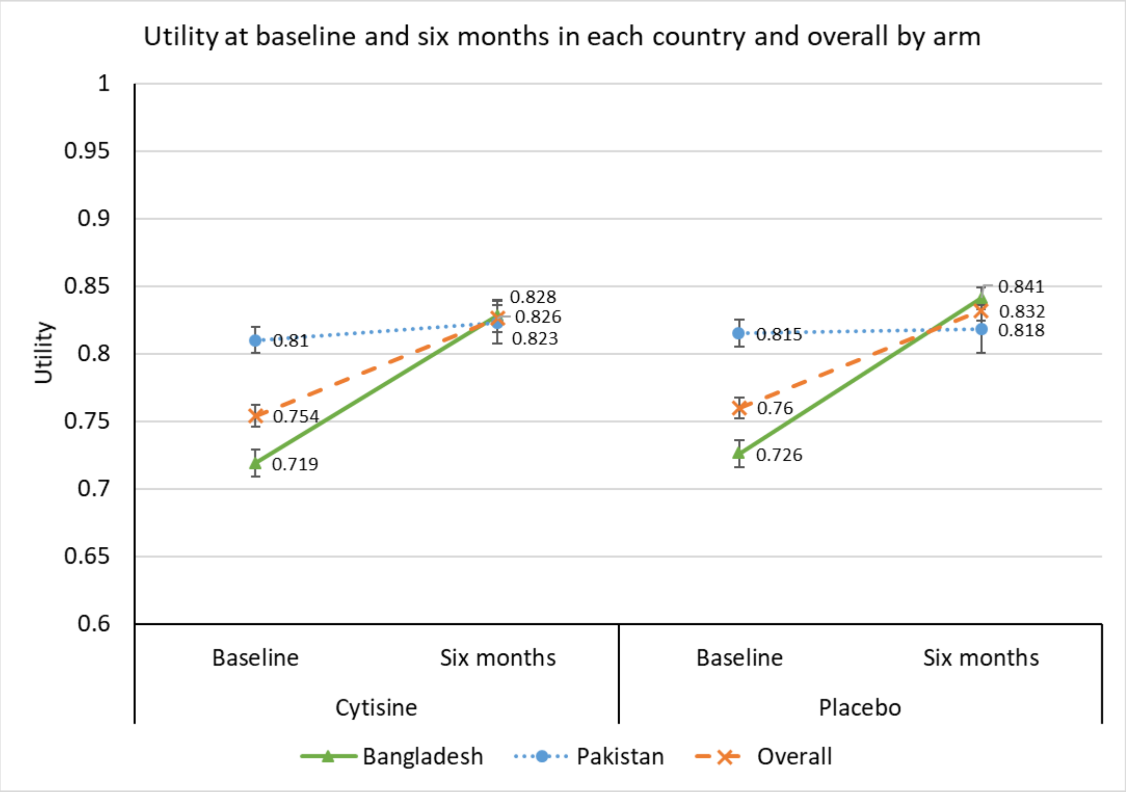

Figure 2 Mean utility at baseline and six months by country and by arm

Adjusting for costs of healthcare and smoking cessation services in the six months before baseline, age, gender, with sites as random effect, the incremental costs over the six months post randomisation were PPP US$108.46 (95%CI PPP US$69.69 to PPP US$157.88) in Pakistan and PPP US$37.06 (95% CI PPP US$28.12 to PPP US$43.85) in Bangladesh (Table 5). Adjusting for utility at baseline, age, gender, with sites as random effect, the incremental QALYs were 0.001 (95% CI -0.004 to 0.008) in Pakistan and -0.003 (95% CI -0.006 to 0.000) in Bangladesh. Therefore, in Pakistan, the ICER was calculated in at PPP US$108,464 per QALY and in Bangladesh, the cytisine arm was dominated by the placebo arm (the cytisine arm being more costly but less effective). Figure 3 shows the uncertainty surrounding the ICERs estimated using bootstrap technique. For Bangladesh, 96% (4794/5000) of the bootstrapped replicates fell in the north-west quadrant of the CEP, where the intervention was more costly but less effective in terms of QALYs. This supports the point estimate that the cytisine arm was dominated by the placebo arm. For Pakistan, 71% (3568/5000) of the bootstrapped replicates fell in the north-east quadrant of the CEP, where the intervention was more costly and more effective in terms of QALYs. The rest fell in the north-west quadrant, indicating a more costly but less effective intervention. According to the estimate made by Woods et al., the willingness-to-pay (WTP) threshold for Pakistan was PPP US$314 to PPP US$2146 per QALY in 2013 [13]. Converting to Pakistan Rupees in 2013 then inflating using consumer price index to 2018 [5 14], the estimated WTP in Pakistan was PPP US$356 to PPP US$2431 per QALY. Represented by the red line in Figure 3, it was apparent that none of the estimates fell under the upper boundary of the WTP (i.e. not cost-effective), same as the point estimate of PPP US$108,464 per QALY. The probability of the cytisine intervention being cost-effective was 0% throughout a wide range of WTP values in both countries, the CEACs were therefore not presented. By these results, the cytisine intervention was unlikely to be cost-effective, comparing with placebo, in either Pakistan or Bangladesh.

Table 5 Cost-utility analysis results by country (1 PPPUS$ = 30.9 Bangladeshi Taka = 29.3 Pakistani Rupees)

**Costs (PPP US$) Mean (SE)**	**Pakistan**		**Bangladesh**	
	**Cytisine (n=476)**	**Placebo (n=469)**	**Cytisine (n=763)**	**Placebo (n=764)**
Intervention	74.37 (0.68)	15.84 (0.03)	52.10 (0.13)	10.23 (0.00)
TB treatment	421.30 (5.43)	412.97 (5.84)	232.69 (0.78)	233.59 (0.61)
Doctor visit	3.17 (0.82)	2.66 (0.65)	3.50 (0.29)	3.37 (0.29)
Hospital stay	78.12 (19.80)	32.70 (9.76)	3.07 (1.62)	7.35 (3.82)
Smoking cessation	0.00 (0.00)	0.00 (0.00)	0.74 (0.06)	0.71 (0.03)
**Overall total for six months**	**576.96 (20.65)**	**464.16 (11.81)**	**292.07 (1.84)**	**255.28 (3.88)**
Adjusted incremental costs	108.46 (95%CI 69.69 to 157.88)		37.06 (95% CI 28.12 to 43.85)	
**QALYs**	**0.408 (0.002)**	**0.408 (0.003)**	**0.387 (0.002)**	**0.392 (0.002)**
Adjusted incremental QALYs	0.001 (95% CI -0.004 to 0.008)		-0.003 (95% CI -0.006 to 0.000)	
ICER	108,464 per QALY (uncertainty see Figure 3)		Cytisine dominated by placebo (uncertainty see Figure 3)	

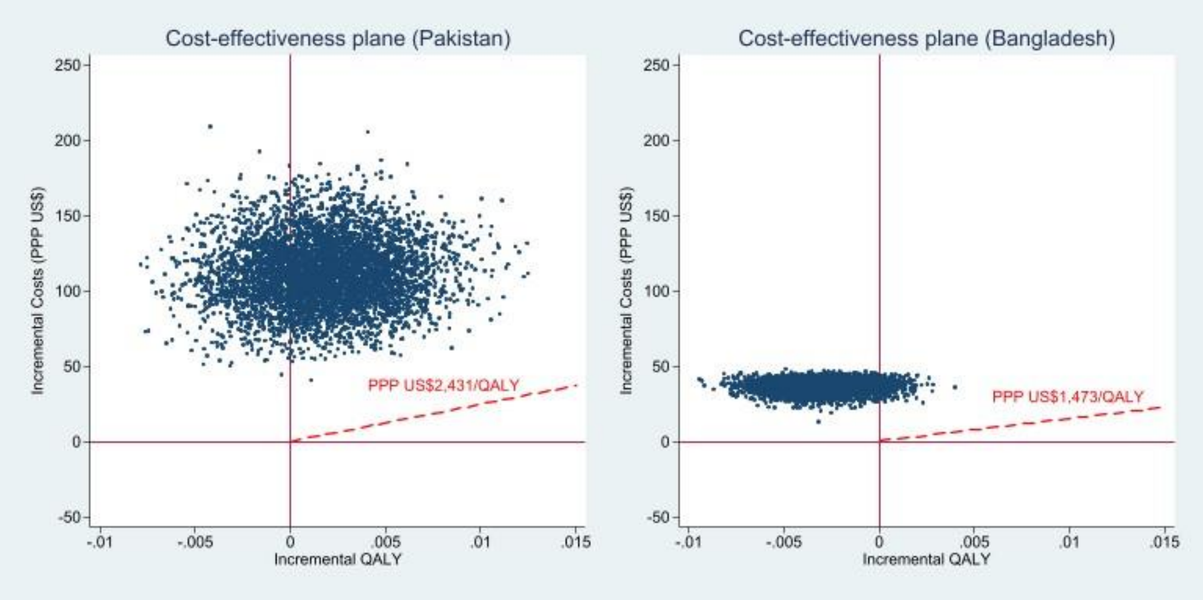

Figure 3 Cost-effectiveness plane of cost-utility analysis results by country

References

1. Bangladesh Bureau of Statistics. Report on Labour Force Survey (LFS) 2016-17, 2018.
2. Pakistan Bureau of Statistics. Labour Force Survey 2017-18, 2018.
3. World Health Organization. Global Tuberculosis report 2018 - TB data: WHO, 2018.
4. Gomez GB, Dowdy DW, Bastos ML, et al. Cost and cost-effectiveness of tuberculosis treatment shortening: a model-based analysis. *BMC Infect Dis* 2016;16(1):726. doi: 10.1186/s12879-016-2064-3 [published Online First: 2016/12/03]
5. The World Bank. DataBank World Development Indicators, 2019.
6. International Monetary Fund. National Currency per U.S. Dollar, period average. Inernational Financial Statistics: International Monetary Fund (IMF), 2019.
7. World Health Organization. Tobacco control country profile Pakistan. Tobacco control country profiles, 2017.
8. World Health Organization. Tobacco control country profile Bangladesh. Tobacco control country profiles, 2017.
9. National Institute for Health and Care Excellence. Putting NICE guidance into practice - Resource impact report: Stop smoking interventions and services (NG92), 2018.
10. Curtis LA, Burns A. Unit Costs of Health and Social Care 2018. Kent: University of Kent, 2018.
11. Economic Analysis and Evaluation Team. Country-specific inpatient and outpatient estimates in 2010 currency: World Health Organisation, 2010.
12. International Monetary Fund. Prices, Consumer Price Index, All items, Index. International Financial Statistics (IFS): International Monetary Fund (IMF), 2019.
13. Woods B, Revill P, Sculpher M, et al. Country-Level Cost-Effectiveness Thresholds: Initial Estimates and the Need for Further Research. *Value Health* 2016;19(8):929-35. doi: 10.1016/j.jval.2016.02.017 [published Online First: 2016/12/19]
14. International Monetary Fund (IMF). Prices, Consumer Price Index, All items, Index. International Financial Statistics (IFS): International Monetary Fund (IMF), 2019.

For peer review only

TB & Tobacco

CASE REPORT FORM - Visit (DAY 0)

Site ID: ☐☐ **Trial Number:** ☐☐☐☐☐☐ **Date of Completion:** ☐☐ / ☐☐ / ☐☐☐☐
Day Month Year

Section V

ECONOMIC EVALUATION *(This section is about the patient's wider health care use in the past six months, unless stated as TB-specific, this is for any illness.)*

Please exclude care provided by the trial intervention in your answers to these questions.

All costs should be specified in local currency, please round all costs up to the nearest whole number.

Enter a number for each item, if none, enter "0" (zero).

Please use this information to guide you if the patient gives estimates-

For daily visit: One week= 7 days, one month= 30 days, three months= 90 days, six months= 180 days.
For weekly visit per month= 4 times.

(i.e "I visited a centre daily for 6 months" would be 180 times)

1. Have you visited a TB clinic in the past six months?
(please exclude current visit and include visits to diagnostic centres if separate from clinics)

☐ Yes ☐ No (go to Q2)

If 'Yes'

a. How many times have you visited a public/voluntary TB clinic? ☐☐☐

b. How many times have you visited a private TB clinic? ☐☐☐

In **total** how much did you pay in the past six months (for consultation, diagnostics, procedures, drugs)? *(in local currency)* ☐☐☐☐☐☐

c. How much did you usually pay for your own travel per visit? *(in local currency)* ☐☐☐☐☐☐

d. On how many of these visits were you accompanied by a friend/relative? ☐☐☐

e. How much time in **total** did it usually take per visit *(travel, waiting, procedure)*? ☐☐ hours ☐☐ minutes

For peer review only - http://bmjopen.bmj.com/site/about/guidelines.xhtml

TB & Tobacco Trial Number: ☐☐☐☐☐☐

2. Have you visited a doctor in the past six months (*for any illness and exclude TB clinic visits recorded in Q1)*?

☐ Yes ☐ No (go to Q3)

If 'Yes'

a. How many times have you visited a public/voluntary doctor? ☐☐☐

In the past six months, in **total** how much did you pay for public/ voluntary visits *(for consultation, diagnostics, procedure, drugs)*? *(in local currency)* ☐☐☐☐☐☐

b. How many times have you visited a private doctor? ☐☐☐

In the past six months, in **total** how much did you pay for private visits *(for consultation, diagnostics, procedure, drugs)*? *(in local currency)* ☐☐☐☐☐☐

c. How much time did you usually spend with the doctor per visit? ☐☐ hours ☐☐ minutes

d. How much did you usually pay for your own travel per visit? *(in local currency)* ☐☐☐☐☐☐

e. On how many of these visits were you accompanied by a friend/relative etc.? ☐☐☐

f. How much time in **total** did it usually take per visit *(travel, waiting, procedure)*? ☐☐ hours ☐☐ minutes

3. Have you been admitted to hospital in the past six months (*for any illness*)?

☐ Yes ☐ No (go to Q4)

If 'Yes'

a. How many nights were you in a public/voluntary hospital? ☐☐☐

In **total** how much did you pay in the past six months at public/voluntary hospitals *(for consultation, diagnostics, procedures, drugs, overnight stay)*? *(in local currency)* ☐☐☐☐☐☐

b. How many nights were you in a private hospital? ☐☐☐

In **total** how much did you pay in the past six months at private hospitals *(for consultation, diagnostics, procedures, drugs, overnight stay)*? *(in local currency)* ☐☐☐☐☐☐

c. How much did you usually pay for your own travel per visit? *(in local currency)* ☐☐☐☐☐☐

For peer review only - http://bmjopen.bmj.com/site/about/guidelines.xhtml

TB & Tobacco   Trial Number: ☐☐☐☐☐☐

4. Have you received any help to stop smoking in the past six months? *(please exclude the behavioural support session immediately before joining the trial, the session provided by the trial and any medication provided by the trial)*

☐ Yes (go to Q5)   ☐ No (go to Q6)

5. How many times in the past six months have you *(this question is only about smoking cessation)*: Enter a number for each item, if none enter '0' (zero).

	Number of times	Amount spent out of pocket *(in local currency)*
Had help or advice about smoking from a public/government clinic/hospital?	☐☐☐	☐☐☐☐☐
Had help or advice about smoking from a private clinic/hospital?	☐☐☐	☐☐☐☐☐
Attended a group or single counselling session on smoking at a public/voluntary clinic?	☐☐☐	☐☐☐☐☐
Attended a group or single counselling session on smoking at a private clinic/hospital?	☐☐☐	☐☐☐☐☐
Been given a prescription for nicotine patches?	☐☐☐	☐☐☐☐☐
Been given a prescription for an alternative form of NRT? (such as gum, lozenge, inhaler, etc)	☐☐☐	☐☐☐☐☐
Bought a refill for an electronic cigarette?	☐☐☐	☐☐☐☐☐
Been given a prescription for Zyban (Bupropion)?	☐☐☐	☐☐☐☐☐
Been given a prescription for Champix (Varenicline)?	☐☐☐	☐☐☐☐☐
Received any traditional medicine? (Hakeem, Homeopathic, Unani etc.)	☐☐☐	☐☐☐☐☐
Other: please describe:	☐☐☐	☐☐☐☐☐

For peer review only

TB & Tobacco

Trial Number: ☐☐☐☐☐☐

6. Have you received any medications for TB in the past six months?

☐ Yes (go to Q7) ☐ No (go to Q8)

7. Please detail below the medications for TB related illness in the past six months? *(Use the colour of the packets to indicate each medication)*

(If patient answers not in days: one week= 7 days, one month= 30 days, three months= 90 days, six months= 180 days etc.)

**Anti-TB medication**	**Number of tablets per day**	**Duration receiving tablets (days)**
Fixed-dose combination (4 drugs)<br>*(R-150mg/H-75mg/E-275mg/P-400mg)*		
Fixed-dose combination (2 drugs)<br>*(R-150mg/H-75mg)*		

8. Do you have a paid job? *(include self-employed and employed) (Please tick one only)*

☐ I have a full time job (go to Q9)

☐ I have a part time job (go to Q9)

☐ I do not have a job (go to Q10)

9. Have you been off work sick in the past six months *(for any illness)*?

☐ Yes

☐ No (go to Q10)

If 'Yes' how many days were you off work sick in the last six months? ☐☐☐

10. Usually how much did you spend **per day** on tobacco over the past six months? *(In local currency)* ☐☐☐☐☐☐

For peer review only - http://bmjopen.bmj.com/site/about/guidelines.xhtml

TB & Tobacco

Trial Number:

EURO QOL

This section asks about your health in general.
Under each heading, please tick the ONE box that best describes your health TODAY.

MOBILITY
- I have no problems in walking about ☐
- I have slight problems in walking about ☐
- I have moderate problems in walking about ☐
- I have severe problems in walking about ☐
- I am unable to walk about ☐

SELF-CARE
- I have no problems washing or dressing myself ☐
- I have slight problems washing or dressing myself ☐
- I have moderate problems washing or dressing myself ☐
- I have severe problems washing or dressing myself ☐
- I am unable to wash or dress myself ☐

USUAL ACTIVITIES *(e.g. work, study, housework, family or leisure activities)*
- I have no problems doing my usual activities ☐
- I have slight problems doing my usual activities ☐
- I have moderate problems doing my usual activities ☐
- I have severe problems doing my usual activities ☐
- I am unable to do my usual activities ☐

PAIN/DISCOMFORT
- I have no pain or discomfort ☐
- I have slight pain or discomfort ☐
- I have moderate pain or discomfort ☐
- I have severe pain or discomfort ☐
- I have extreme pain or discomfort ☐

ANXIETY/DEPRESSION
- I am not anxious or depressed ☐
- I am slightly anxious or depressed ☐
- I am moderately anxious or depressed ☐
- I am severely anxious or depressed ☐
- I am extremely anxious or depressed ☐

UK (English) © 2009 EuroQol Group. EQ-5D™ is a trade mark of the EuroQol Group

For peer review only - http://bmjopen.bmj.com/site/about/guidelines.xhtml
For peer review only

TB & Tobacco

Trial Number:

The best health
you can imagine

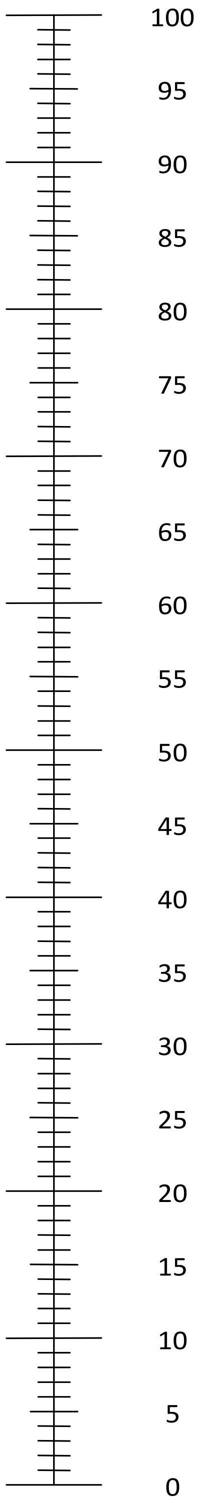

- We would like to know how good or bad your health is TODAY.
- The scale is numbered from 0 to 100.
- 100 means the best health you can imagine.

  0 means the worst health you can imagine.
- Mark an X on the scale to indicate how your health is TODAY.
- Now, please write the number you marked on the scale in the box below.

YOUR HEALTH TODAY =

For peer review only

The worst health
you can imagine

UK (English) © 2009 EuroQol Group. EQ-5D™ is a trade mark of the EuroQol Group

Please dispense medication for 1 week. Instruct the participant to come back for follow up coinciding with their quit date and also to bring the blister packets and the 'dosing schedule card'.

Thank you for your time!

Send data

For peer review only - http://bmjopen.bmj.com/site/about/guidelines.xhtml

2624566506

Supplementary tables

Table S1 Average hourly wage by occupation in Pakistan and Bangladesh

	Average hourly wage (PPP US$) [1-3]						
	**Bangladesh**				**Pakistan**		
**Occupation**	**Male**	**Female**	**Total**		**Male**	**Female**	**Total**
Managers	5.68	5.13	5.62		9.67	0.84	9.57
Professionals	4.25	3.93	4.13		6.06	3.84	5.30
Technicians and Associate Professionals	3.35	3.21	3.32		4.69	3.27	4.50
Clerical support workers	2.56	2.33	2.53		4.69	3.16	4.66
Service and Sales workers	1.88	1.76	1.86		2.85	2.37	2.83
Skilled Agricultural, forestry and fisheries	1.50	1.24	1.46		3.03	0.98	2.96
Craft and Related Trades workers	1.69	1.55	1.65		3.00	0.89	2.67
Plant and Machine Operators, and Assembler	1.91	1.77	1.89		2.96	1.95	2.95
Elementary Occupations	1.38	1.15	1.32		2.39	1.11	2.15
Overall	2.14	1.93	2.09		3.35	2.00	3.15

For peer review only

Table S2 Number and proportion of missing values of variables by arm

	Cytisine (n=1239)		Placebo (n=1233)	
Variables	Number of missing values	Proportion of missing values	Number of missing values	Proportion of missing values
Cost of behavioural support	6	0%	7	1%
Cost of TB treatment	102	8%	103	8%
Cost of doctor visit at d0	0	0%	1	0%
Cost of doctor visit at m6	61	5%	67	5%
Cost of doctor visit at m12	89	7%	76	6%
Cost of hospital stay at d0	2	0%	2	0%
Cost of hospital stay at m6	62	5%	67	5%
Cost of hospital stay at m12	89	7%	76	6%
Cost of smoking cessation at d0	0	0%	0	0%
Cost of smoking cessation at m6	63	5%	69	6%
Cost of smoking cessation at m12	103	8%	89	7%
OOP on TB treatment at d0	1	0%	0	0%
OOP on TB treatment at m6	63	5%	69	6%
OOP on TB treatment at m12	89	7%	77	6%
OOP on smoking cessation at d0	3	0%	3	0%
OOP on smoking cessation at m6	146	12%	153	12%
OOP on smoking cessation at m12	127	10%	118	10%
OOP on doctor visit at d0	6	0%	6	0%
OOP on doctor visit at m6	64	5%	75	6%
OOP on doctor visit at m12	89	7%	76	6%
OOP on hospital stay at d0	2	0%	2	0%
OOP on hospital stay at m6	64	5%	69	6%
OOP on hospital stay at m12	89	7%	76	6%
OOP on tobacco products d0	10	1%	9	1%
OOP on tobacco products m6	60	5%	67	5%
OOP on tobacco products m12	89	7%	76	6%
Productivity loss of company for TB treatment at d0	7	1%	5	0%
Productivity loss of company for TB treatment at m6	103	8%	106	9%
Productivity loss of company for TB treatment at m12	92	7%	81	7%
Productivity loss of company for doctor at d0	36	3%	37	3%
Productivity loss of company for doctor at m6	111	9%	117	9%
Productivity loss of company for doctor at m12	94	8%	82	7%
Productivity loss of sick leave at d0	9	1%	6	0%
Productivity loss of sick leave at m6	44	4%	62	5%

For peer review only

	Cytisine (n=1239)		Placebo (n=1233)	
Variables	Number of missing values	Proportion of missing values	Number of missing values	Proportion of missing values
Productivity loss of sick leave at m12	75	6%	73	6%
EQ-5D-5L at d0				
1 Mobility	0	0%	0	0%
2 Self-care	0	0%	2	0%
3 Usual activities	2	0%	0	0%
4 Pain and discomfort	1	0%	1	0%
5 Anxiety or depression	2	0%	1	0%
EQ-5D-5L at m6				
1 Mobility	60	5%	67	5%
2 Self-care	60	5%	67	5%
3 Usual activities	60	5%	68	6%
4 Pain and discomfort	60	5%	68	6%
5 Anxiety or depression	60	5%	68	6%
EQ-5D-5L at m12				
1 Mobility	89	7%	76	6%
2 Self-care	89	7%	78	6%
3 Usual activities	89	7%	78	6%
4 Pain and discomfort	90	7%	78	6%
5 Anxiety or depression	94	8%	78	6%
VAS at d0	0	0%	0	0%
VAS at m6	60	5%	68	6%
VAS at m12	89	7%	77	6%
TB score at d0	0	0%	0	0%
TB score at m6	60	5%	66	5%

For peer review only

Table S3 Logistic regression for missingness of costs, OOPs, productivity loss and outcomes on arm and baseline covariates

Missing on:	Allocation	Age	Country
Cost of TB treatment	1.02 (0.76-1.35)	1.02 (1.01-1.03)	**0.26 (0.19-0.36)***
Cost of doctor visit at m6	1.07 (0.75-1.53)	1.00 (0.99-1.01)	**0.15 (0.10-0.24)***
Cost of doctor visit at m12	0.83 (0.61-1.14)	1.00 (0.99-1.01)	**0.17 (0.12-0.25)***
Cost of hospital stay at m6	1.05 (0.74-1.50)	1.00 (0.99-1.01)	**0.16 (0.11-0.24)***
Cost of hospital stay at m12	0.83 (0.61-1.14)	1.00 (0.99-1.01)	**0.17 (0.12-0.25)***
Cost of smoking cessation at m6	1.07 (0.76-1.52)	1.00 (0.99-1.01)	**0.17 (0.11-0.26)***
Cost of smoking cessation at m12	0.84 (0.63-1.13)	1.00 (0.99-1.01)	**0.28 (0.21-0.39)***
OOP on TB treatment at m6	1.07 (0.76-1.52)	1.00 (0.99-1.01)	**0.16 (0.10-0.24)***
OOP on TB treatment at m12	0.84 (0.62-1.15)	1.00 (0.99-1.01)	**0.17 (0.12-0.25)***
OOP on smoking cessation at m6	1.04 (0.82-1.33)	**0.99 (0.98-1.00)***	1.14 (0.89-1.47)
OOP on smoking cessation at m12	0.91 (0.70-1.18)	0.99 (0.98-1.00)	**0.52 (0.40-0.68)***
OOP on doctor visit at m6	1.15 (0.82-1.62)	1.00 (0.99-1.01)	**0.16 (0.10-0.24)***
OOP on doctor visit at m12	0.83 (0.61-1.14)	1.00 (0.99-1.01)	**0.17 (0.12-0.25)***
OOP on hospital stay at m6	1.05 (0.74-1.49)	1.00(0.99-1.01)	**0.16 (0.11-0.24)***
OOP on hospital stay at m12	0.83 (0.61-1.14)	1.00 (0.99-1.01)	**0.17 (0.12-0.25)***
OOP on tobacco products m6	1.09 (0.76-1.55)	1.00 (0.99-1.01)	**0.16 (0.10-0.24)***
OOP on tobacco products m12	0.83 (0.61-1.14)	1.00 (0.99-1.01)	**0.17 (0.12-0.25)***
Productivity loss of company for TB treatment at m6	1.01 (0.77-1.35)	0.99 (0.98-1.00)	**0.55 (0.41-0.73)***
Productivity loss of company for TB treatment at m12	0.86 (0.63-1.16)	1.00 (0.99-1.01)	**0.18 (0.13-0.26)***
Productivity loss of company for doctor at m6	1.04 (0.80-1.37)	0.99 (0.98-1.00)	**0.52 (0.40-0.69)***
Productivity loss of company for doctor at m12	0.85 (0.63-1.15)	1.00 (0.99-1.01)	**0.18 (0.12-0.25)***
Productivity loss of sick leave at m6	1.40 (0.95-2.08)	0.99 (0.97-1.00)	**0.22 (0.14-0.33)***
Productivity loss of sick leave at m12	0.95 (0.68-1.32)	0.99 (0.98-1.00)	**0.27 (0.19-0.38)***
EQ-5D-5L at m6	1.09 (0.76-1.55)	1.00 (0.99-1.01)	**0.16 (0.10-0.24)***
EQ-5D-5L at m12	0.83 (0.61-1.14)	1.00 (0.99-1.01)	**0.17 (0.12-0.25)***
TB score at m6	1.02 (0.72-1.44)	1.00 (0.99-1.01)	**0.18 (0.12-0.26)***

***P<0.05**

References

1. Bangladesh Bureau of Statistics. Report on Labour Force Survey (LFS) 2016-17, 2018.
2. Pakistan Bureau of Statistics. Labour Force Survey 2017-18, 2018.
3. The World Bank. DataBank World Development Indicators, 2019.

For peer review only

CHEERS Checklist
Items to include when reporting economic evaluations of health interventions

The **ISPOR CHEERS Task Force Report**, *Consolidated Health Economic Evaluation Reporting Standards (CHEERS)—Explanation and Elaboration: A Report of the ISPOR Health Economic Evaluations Publication Guidelines Good Reporting Practices Task Force*, provides examples and further discussion of the 24-item CHEERS Checklist and the CHEERS Statement. It may be accessed via the *Value in Health* or via the ISPOR Health Economic Evaluation Publication Guidelines – CHEERS: Good Reporting Practices webpage: http://www.ispor.org/TaskForces/EconomicPubGuidelines.asp

Section/item	Item No	Recommendation	Reported on page No/ line No
**Title and abstract**			
Title	1	Identify the study as an economic evaluation or use more specific terms such as "cost-effectiveness analysis", and describe the interventions compared.	P1 L1-4
Abstract	2	Provide a structured summary of objectives, perspective, setting, methods (including study design and inputs), results (including base case and uncertainty analyses), and conclusions.	P2
**Introduction**			
Background and objectives	3	Provide an explicit statement of the broader context for the study. Present the study question and its relevance for health policy or practice decisions.	P4 L2-23
**Methods**			
Target population and subgroups	4	Describe characteristics of the base case population and subgroups analysed, including why they were chosen.	P4 L35-P5 L7
Setting and location	5	State relevant aspects of the system(s) in which the decision(s) need(s) to be made.	P4 6-12, P5 L3-5
Study perspective	6	Describe the perspective of the study and relate this to the costs being evaluated.	P4 L20-23, P7 L6-11
Comparators	7	Describe the interventions or strategies being compared and state why they were chosen.	P5 L10-17
Time horizon	8	State the time horizon(s) over which costs and consequences are being evaluated and say why appropriate.	P7 L6, P4 L6-8
Discount rate	9	Report the choice of discount rate(s) used for costs and outcomes and say why appropriate.	P7 L11-12
Choice of health outcomes	10	Describe what outcomes were used as the measure(s) of benefit in the evaluation and their relevance for the type of analysis performed.	P6 L23-35, P7 L6
Measurement of effectiveness	11a	*Single study-based estimates:* Describe fully the design features of the single effectiveness study and why the single study was a sufficient source of clinical effectiveness data.	P4 L14-30

For peer review only

	11b	*Synthesis-based estimates:* Describe fully the methods used for identification of included studies and synthesis of clinical effectiveness data.	N/A
Measurement and valuation of preference based outcomes	12	If applicable, describe the population and methods used to elicit preferences for outcomes.	P6 L31-35
Estimating resources and costs	13a	*Single study-based economic evaluation:* Describe approaches used to estimate resource use associated with the alternative interventions. Describe primary or secondary research methods for valuing each resource item in terms of its unit cost. Describe any adjustments made to approximate to opportunity costs.	P5 L19-P6 L22, Supporting information 1
	13b	*Model-based economic evaluation:* Describe approaches and data sources used to estimate resource use associated with model health states. Describe primary or secondary research methods for valuing each resource item in terms of its unit cost. Describe any adjustments made to approximate to opportunity costs.	N/A
Currency, price date, and conversion	14	Report the dates of the estimated resource quantities and unit costs. Describe methods for adjusting estimated unit costs to the year of reported costs if necessary. Describe methods for converting costs into a common currency base and the exchange rate.	P5 L19-23
Choice of model	15	Describe and give reasons for the specific type of decision-analytical model used. Providing a figure to show model structure is strongly recommended.	N/A
Assumptions	16	Describe all structural or other assumptions underpinning the decision-analytical model.	N/A
Analytical methods	17	Describe all analytical methods supporting the evaluation. This could include methods for dealing with skewed, missing, or censored data; extrapolation methods; methods for pooling data; approaches to validate or make adjustments (such as half cycle corrections) to a model; and methods for handling population heterogeneity and uncertainty.	P6 L38-P7 L36
**Results**			
Study parameters	18	Report the values, ranges, references, and, if used, probability distributions for all parameters. Report reasons or sources for distributions used to represent uncertainty where appropriate. Providing a table to show the input values is strongly recommended.	Table 1, Table S1, Supporting information 1
Incremental costs and outcomes	19	For each intervention, report mean values for the main categories of estimated costs and outcomes of interest, as well as mean differences between the comparator groups. If applicable, report incremental cost-effectiveness ratios.	P8 L15-25, Table 2
Characterising uncertainty	20a	*Single study-based economic evaluation:* Describe the effects of sampling uncertainty for the estimated incremental cost and incremental effectiveness parameters, together with the impact	P8 L28-40, Figure 1-2

For peer review only

		of methodological assumptions (such as discount rate, study perspective).	
	20b	*Model-based economic evaluation:* Describe the effects on the results of uncertainty for all input parameters, and uncertainty related to the structure of the model and assumptions.	N/A
Characterising heterogeneity	21	If applicable, report differences in costs, outcomes, or cost-effectiveness that can be explained by variations between subgroups of patients with different baseline characteristics or other observed variability in effects that are not reducible by more information.	N/A
**Discussion**			
Study findings, limitations, generalisability, and current knowledge	22	Summarise key study findings and describe how they support the conclusions reached. Discuss limitations and the generalisability of the findings and how the findings fit with current knowledge.	P9 L21-P10 L9
**Other**			
Source of funding	23	Describe how the study was funded and the role of the funder in the identification, design, conduct, and reporting of the analysis. Describe other non-monetary sources of support.	P10 L21-24
Conflicts of interest	24	Describe any potential for conflict of interest of study contributors in accordance with journal policy. In the absence of a journal policy, we recommend authors comply with International Committee of Medical Journal Editors recommendations.	P10 L29-37

For consistency, the CHEERS Statement checklist format is based on the format of the CONSORT statement checklist

The **ISPOR CHEERS Task Force Report** provides examples and further discussion of the 24-item CHEERS Checklist and the CHEERS Statement. It may be accessed via the *Value in Health* link or via the ISPOR Health Economic Evaluation Publication Guidelines – CHEERS: Good Reporting Practices webpage: http://www.ispor.org/TaskForces/EconomicPubGuidelines.asp

The citation for the CHEERS Task Force Report is:
Husereau D, Drummond M, Petrou S, et al. Consolidated health economic evaluation reporting standards (CHEERS)—Explanation and elaboration: A report of the ISPOR health economic evaluations publication guidelines good reporting practices task force. Value Health 2013;16:231-50.

For peer review only

Cost-utility of cytisine for smoking cessation over and above behavioural support in people with newly diagnosed pulmonary tuberculosis: An economic evaluation of a multi-centre randomised controlled trial

Journal:	*BMJ Open*
Manuscript ID	bmjopen-2021-049644.R2
Article Type:	Original research
Date Submitted by the Author:	25-Jul-2022
Complete List of Authors:	Li, Jinshuo; University of York Department of Health Sciences<br>Parrott, Steve; University of York Department of Health Sciences<br>Keding, Ada; University of York Department of Health Sciences<br>Dogar, Omara; University of York Department of Health Sciences; The University of Edinburgh Usher Institute of Population Health Sciences and Informatics<br>Gabe, Rhian; Queen Mary University of London, Centre for Cancer Prevention, Wolfson Institute of Preventive Medicine<br>Marshall, Anna-Marie; University of York Department of Health Sciences<br>Huque, Rumana; University of Dhaka Department of Economics; ARK Foundation<br>Barua, Deepa; ARK Foundation<br>Fatima, Razia; Global Fund Grant, TB/HIV and Malaria Common Management Unit<br>Khan, Amina; The Initiative<br>Zahid, Raana; The Initiative<br>Mansoor, Sonia; Rawalpindi Medical University, Institute of Psychiatry<br>Kotz, Daniel; Heinrich-Heine-Universitat Dusseldorf, Institute of General Practice, Addiction Research and Clinical Epidemiology Unit; The University of Edinburgh Usher Institute of Population Health Sciences and Informatics<br>Boeckmann, Melanie; Heinrich-Heine-Universität Düsseldorf, Institute of General Practice, Addiction Research and Clinical Epidemiology Unit, Medical faculty; University of York, Department of Health Sciences<br>Elsey, Helen ; University of York Department of Health Sciences<br>Kralikova, Eva; Charles University, Institute of Hygiene and Epidemiology; General University Hospital in Prague, 3rd Medical Department<br>Readshaw, Anne; University of York, Department of Health Sciences<br>Sheikh, Aziz; The University of Edinburgh Usher Institute of Population Health Sciences and Informatics<br>Siddiqi, Kamran; University of York Department of Health Sciences; Hull York Medical School
**Primary Subject Heading**:	Health economics
Secondary Subject Heading:	Global health, Health economics, Smoking and tobacco, Public health

For peer review only - http://bmjopen.bmj.com/site/about/guidelines.xhtml

Keywords:	Tuberculosis < INFECTIOUS DISEASES, HEALTH ECONOMICS, Clinical trials < THERAPEUTICS, International health services < HEALTH SERVICES ADMINISTRATION & MANAGEMENT, PUBLIC HEALTH

SCHOLARONE™
Manuscripts

I, the Submitting Author has the right to grant and does grant on behalf of all authors of the Work (as defined in the below author licence), an exclusive licence and/or a non-exclusive licence for contributions from authors who are: i) UK Crown employees; ii) where BMJ has agreed a CC-BY licence shall apply, and/or iii) in accordance with the terms applicable for US Federal Government officers or employees acting as part of their official duties; on a worldwide, perpetual, irrevocable, royalty-free basis to BMJ Publishing Group Ltd ("BMJ") its licensees and where the relevant Journal is co-owned by BMJ to the co-owners of the Journal, to publish the Work in this journal and any other BMJ products and to exploit all rights, as set out in our licence.

The Submitting Author accepts and understands that any supply made under these terms is made by BMJ to the Submitting Author unless you are acting as an employee on behalf of your employer or a postgraduate student of an affiliated institution which is paying any applicable article publishing charge ("APC") for Open Access articles. Where the Submitting Author wishes to make the Work available on an Open Access basis (and intends to pay the relevant APC), the terms of reuse of such Open Access shall be governed by a Creative Commons licence – details of these licences and which Creative Commons licence will apply to this Work are set out in our licence referred to above.

Other than as permitted in any relevant BMJ Author's Self Archiving Policies, I confirm this Work has not been accepted for publication elsewhere, is not being considered for publication elsewhere and does not duplicate material already published. I confirm all authors consent to publication of this Work and authorise the granting of this licence.

For peer review only

Cost-utility of cytisine for smoking cessation over and above behavioural support in people with newly diagnosed pulmonary tuberculosis: An economic evaluation of a multi-centre randomised controlled trial

Jinshuo Li, M.Phil[1], Steve Parrott, MSc[1], Ada Keding, MSc[1], Omara Dogar, PhD[1,2], Rhian Gabe, PhD[3], Anna-Marie Marshall, PhD[1], Rumana Huque, PhD[4, 5], Deepa Barua, MPH[4], Razia Fatima, PhD[6], Amina Khan, MPH[7], Raana Zahid, MPH[7], Sonia Mansoor, MSc[8], Daniel Kotz, PhD[2,9,10], Melanie Boeckmann, DrPH[1,9] , Helen Elsey, PhD, FFPH[1], Eva Kralikova, PhD, MD[11, 12], Anne Readshaw, PhD[1], Aziz Sheikh, MD[2] & Kamran Siddiqi, PhD[1,13], *on behalf of the TB & Tobacco Consortium*

Authors' affiliations

[1]University of York, Department of Health Sciences, Faculty of Sciences, YO10 5DD, York, UK

[2]Usher Institute, The University of Edinburgh, EH8 9AG, Edinburgh, UK

[3] Centre for Cancer Prevention, Wolfson Institute of Preventive Medicine, Queen Mary University of London, United Kingdom

[4]ARK Foundation, Dhaka, Bangladesh

[5]Department of Economics, University of Dhaka, Bangladesh

[6]TB/HIV and Malaria Common Management Unit, Global Fund Grant, Islamabad, Pakistan

[7]The Initiative, Islamabad, Pakistan

[8] Institute of Psychiatry (IOP) Rawalpindi Medical University, Pakistan

[9]Institute of General Practice, Addiction Research and Clinical Epidemiology Unit, Medical Faculty of the Heinrich-Heine-University, Düsseldorf, Germany

[10]Department of Family Medicine, CAPHRI School for Public Health and Primary Care, Maastricht University, The Netherlands

[11]3rd Medical Department, First Faculty of Medicine, Charles University, and the General University Hospital in Prague, Czech Republic

[12]Institute of Hygiene and Epidemiology, First Faculty of Medicine, Charles University, and the General University Hospital in Prague, Czech Republic

[13]Hull York Medical School, University of York, YO10 5DD, York, UK

Corresponding author: Jinshuo Li, e-mail: jinshuo.li@york.ac.uk, tel: +44 1904 32 1358, Area 4 ARRC building, Department of Health Sciences, University of York, Heslington, YO10 4DU, York, United Kingdom

Word count: 4217

For peer review only - http://bmjopen.bmj.com/site/about/guidelines.xhtml

Abstract

Objectives

To assess the cost-effectiveness of cytisine over and above brief behavioural support (BS) for smoking cessation among newly diagnosed pulmonary tuberculosis (TB) patients in low- and middle-income countries.

Design

An incremental cost-utility analysis was undertaken alongside a 12-month double-blind two-arm individually randomised controlled trial from a public/voluntary healthcare sector perspective, with the primary endpoint at six months post-randomisation.

Setting

Seventeen sub-district hospitals in Bangladesh and 15 secondary care hospitals in Pakistan.

Participants

Adults (aged ≥18 years in Bangladesh and ≥15 years in Pakistan) with pulmonary TB diagnosed within the last four weeks, who smoked tobacco daily (n=2472).

Interventions

Two brief BS sessions with a trained TB health worker were offered to all participants. Participants in the intervention arm (n=1239) were given cytisine (25-day course) while those in the control arm (n=1233) were given placebo. No significant difference was found between arms in six-month abstinence.

Primary and secondary outcome measures

Costs of cytisine and BS sessions were estimated based on research team records. TB treatment costs were estimated based on TB registry records. Additional smoking cessation and healthcare costs and EQ-5D-5L data were collected at baseline, six- and 12-month follow-ups. Costs were presented in Purchasing Power Parity (PPP) adjusted US dollars (US$). Quality-adjusted life years (QALYs) were derived from the EQ-5D-5L. Incremental total costs and incremental QALYs were estimated using regressions adjusting for respective baseline values and other baseline covariates. Uncertainty was assessed using bootstrapping.

Results

Mean total costs were PPP US$57.74 (95% CI 49.40 – 83.36) higher in the cytisine arm than in the placebo arm while the mean QALYs were -0.001 (95% CI -0.004 – 0.002) lower over six months. The cytisine arm was dominated by the placebo arm.

Conclusions

Cytisine *plus* BS for smoking cessation among TB patients was not cost-effective compared to placebo *plus* BS.

Trial registration number: International Standard Randomized Clinical Trial Number, ISRCTN43811467.

For peer review only

For peer review only - http://bmjopen.bmj.com/site/about/guidelines.xhtml

Strength and limitations of this study

- Large sample size and high follow-up rate ensures robustness of the conclusion.
- Comprehensive patient-level data collection provides possibilities of further exploration or updating of the analyses.
- Trial across two countries posed challenges to value both costs and quality-adjusted life years comparably.
- Lack of up-to-date data sources of unit costs of healthcare services may affect the accuracy of the costs estimation.
- Eagerness of local staff participating in the trial may affect the generalisability of the intervention delivery.

For peer review only

For peer review only - http://bmjopen.bmj.com/site/about/guidelines.xhtml

INTRODUCTION

In 2020, due to the impact of COVID-19 pandemic, the number of newly diagnosed tuberculosis (TB) case notifications saw a big drop from 2019 while the number of people who died from TB increased due to reduced access to services at global, regional, and country levels.[1] Bangladesh (218 per 100,00 population) and Pakistan (259 per 100,000 population) are among the 16 countries that contributed most to the global shortfall of TB notifications yet they are still on the World Health Organization high-burden countries lists for TB and multidrug-resistant TB or rifampicin-resistant TB.[1, 2] Meanwhile, the 2020 estimates of current tobacco smoking rates were 18.5% in Bangladesh and 24.6% in Pakistan, with considerable imbalance between men and women.[3] Previous evidence suggests that continued tobacco smoking among TB patients is associated with unfavourable TB treatment outcomes.[4] However, with the combined burden of TB and tobacco, support for smoking cessation for TB patients is absent in both countries.[5]

TB treatment, lasting six months or longer, offers an opportunity for regular support for quitting smoking, if integrated properly. Newly diagnosed TB patients who smoke might be more receptive to advice to quit due to their immediate health concerns.[6] Due to limited resources, evidence-based approaches such as behavioural support (BS) and expensive pharmacotherapies for smoking cessation cannot be implemented in many low- and middle-income countries (LMICs). We have previously developed, in collaboration with local teams in Bangladesh and Pakistan, a brief BS integrated with routine TB appointment for smoking cessation.[7] In the present study, over-and-above the BS, we examined the effectiveness and cost-effectiveness of the relatively low cost pharmacotherapy cytisine for smoking cessation in TB patients.[8]

We conducted a 12-month, two-arm, parallel, double blind, placebo-controlled, multicentre, individually randomised trial in Bangladesh and Pakistan to compare cytisine plus BS for smoking cessation (cytisine arm: n=1239) with placebo plus BS (placebo arm: n=1233) among pulmonary TB patients who smoke daily.[9] Biochemically-verified continuous abstinence at 6 months (primary endpoint) was 32.4% (401/1239) in the cytisine arm and 29.7% (366/1233) in the placebo arm (RR=1.09, 95% CI 0.97-1.23) and at 12 months, it was 24.9% (309/1239) and 22.3% (275/1233), respectively (RR=1.22, 95% CI 0.95-5.98), indicating no significant difference between arms in the primary outcome.[10] This article reports a set of analyses to, respectively: 1) evaluate the cost-utility, from a public or voluntary healthcare sector perspective, of adding cytisine to BS for smoking cessation in TB patients who smoke; and 2) assess the financial burden in relation to tobacco use and healthcare from participants and their families' perspective, and estimate productivity loss using lost income.

METHODS

Design

An incremental cost-utility analysis was conducted alongside the randomised controlled trial (RCT) described above and elsewhere.[9, 10] The scheduled follow-ups were at 6 and 12 months post-randomisation, with 6 months as the primary endpoint. Neither participants nor TB health workers were aware of participants' arm allocation. Allocation was not revealed to health economists until database lock. Detailed information on procedures was provided in the study protocol.[9]

For peer review only - http://bmjopen.bmj.com/site/about/guidelines.xhtml

Participants

Adults (aged ≥18 in Bangladesh and ≥15 in Pakistan) with pulmonary TB diagnosed within the last four weeks who smoked tobacco on a daily basis and were interested in quitting, were eligible.[9] We excluded those who were diagnosed with TB complications (retreatment or any drug resistance), extra-pulmonary TB, receiving Streptomycin and/or Para Amino Salicylic Acid, using any pharmacotherapy for tobacco dependence, pregnant or planning to become pregnant, lactating, or suffering from schizophrenia or known to be diagnosed with epilepsy. Those who had myocardial infarction, stroke, or an attack of severe angina within the previous two weeks, uncontrolled high blood pressure despite being on medication, or severe renal impairment (requiring dialysis), were also excluded.

Between June 2017 and April 2018, 1527 participants from 17 sub-district hospitals in Bangladesh and 945 participants from 15 secondary care hospitals in Pakistan were randomised to the cytisine arm (n=1239) and the placebo arm (n=1233). The mean age was 42.5 (SD 14.3) years in the cytisine arm and 42.4 (SD 14.2) years in the placebo arm. Men made up 99% of each arm (1227 in the cytisine arm, 1221 in the placebo arm). By 6 months follow-up, 70 participants died (36 in the cytisine arm and 34 in the placebo arm). A further 21 participants died after 6 months (13 in the cytisine arm and eight in the placebo arm).

Intervention and comparator

Participants in the cytisine (intervention) arm were provided with cytisine (Desmoxan, Aflofarm) according to its standard regimen: 38 capsules on day 0 and another 62 capsules on day 5 (pre-set quit date), totalling 100 capsules over a 25-day course. The trial medication was in the form of 1.5mg hard capsules for oral administration.[9, 10]. Participants in the placebo (comparator) arm were given placebo capsules with identical appearance on the same dispensing schedule. In addition, participants in both arms were offered brief BS for smoking cessation delivered by trained TB health workers, accompanied with a leaflet containing information on tobacco use and its interactions with TB for each participant. The BS was designed to be two face-to-face sessions on days 0 (10 minutes) and 5 (5 minutes). Therefore, the intervention consisted of cytisine plus BS while the comparator was placebo plus BS.

Measures

All monetary outcomes were collected or valued in local currencies and inflated to their respective 2018 values [11] where necessary, and converted to purchasing power parity adjusted US dollars (PPP US$) using the World Bank exchange rate in the same year (1 PPPUS$ = 30.9 Bangladeshi Taka = 29.3 Pakistani Rupees).[12] PPP US$ accounts for the price and income difference between the two countries so that the monetary outcomes could be pooled together. Results of costs were presented in PPP US$ 2018 price.

Costs

Intervention costs

Intervention costs included costs of training and delivery (Details see Supplementary file 1). TB health workers were trained in brief BS for smoking cessation in a two-day programme. The costs of training were estimated by the research team to be PPP US$14,183 in Bangladesh and PPP US$12,837 in Pakistan. Since all participants were scheduled to receive BS, the training cost was allocated to each participant evenly.

For peer review only - http://bmjopen.bmj.com/site/about/guidelines.xhtml

The uptake of BS was recorded on the Case Report Form (CRF) on day 0. Staff costs for BS were estimated by multiplying the duration by the hourly wage rate. The cost of BS for the first and second session was PPP US$0.52 and PPP US$0.26 in Bangladesh, and PPP US$0.75 and PPP US$0.38 in Pakistan. For those whose CRF showed not taking up BS, the cost of BS delivery was considered null. For those who accepted BS, the cost of the first session was applied and the cost of the second session was added provided they attended the follow-up on day 5. The smoking cessation information leaflet costed PPP US$0.16 in Bangladesh and PPP US$1.71 in Pakistan, per participant.

The manufacturer provided the distributor price as 72.63 Polish Złoty for 100 capsule pack (PPP US$42.27 in Bangladesh, PPP US$65.09 in Pakistan). By dispensing schedule, the medication dispensed on day 0 costed PPP US$16.05 in Bangladesh and PPP US$24.74 in Pakistan, and on day 5 it costed PPP US$26.21 and PPP US$40.34, respectively. The placebo capsules were assumed to incur no cost. All participants had at least the first dispense and those who missed follow-up on day 5 were assumed not to receive the second dispense.

Costs of TB treatment, additional smoking cessation help, and general healthcare services

Table 1 presents the unit costs of TB treatment by phase, additional smoking cessation services, and general healthcare services, estimated based on secondary sources and some assumptions and converted to PPP US$ 2018.[12-22] Detailed methods of estimation see Supplementary file 1. TB treatment progression was estimated according to the TB registry card. The quantities of services use were collected by self-report at baseline, 6- and 12-month follow-ups (See Supplementary file 2 for CRF).

[Insert Table 1 here]

Out-of-pocket payments (OOPs) and productivity loss

Participants reported any spending in monetary form related to TB treatment, smoking cessation products, and general healthcare services use, including travel, on CRFs at baseline, 6- and 12-month follow-ups.

CRFs also collected participants' time spent in TB clinics and doctor visits, including travel and waiting time, and if and how many times they were accompanied by a friend or relative. The productivity loss of a companion was estimated by multiplying the overall time spent by the companion by the societal average hourly wage in the country.[20, 21] We assumed that all companions were employed. Participants' productivity loss was estimated based on their self-reported duration of sick leave from work. Participants' hourly wages were extracted from secondary sources based on their occupation category and gender,[20, 21] with those reported in open question re-classified according to the International Standard Classification of Occupations ISCO-08 (Supplementary file 3 Table S1).[23] Those who were unemployed, retired, students or home makers, were assumed to incur no productivity loss in the case of sick leave.

Quality-adjusted life years (QALYs)

The EQ-5D-5L developed by the EuroQol Group was used to measure health-related quality of life,[24] at baseline, 6- and 12-month follow-ups, as part of the CRFs. The EQ-5D-5L consists of a descriptive system of five domains (Mobility, Self-care, Usual activities, Pain/Discomfort and Anxiety/Depression), and a Visual Analogue Scale (VAS) valuing the overall health on the day. The VAS score ranges from 0 (death) to 100 (perfect health). Each domain of the descriptive system has five levels of capacity, ranging from having no problem to having severe problems. A complete descriptive system could be converted to a utility value using an appropriate tariff.

For peer review only - http://bmjopen.bmj.com/site/about/guidelines.xhtml

In the absence of country-specific valuation sets for Bangladesh and Pakistan, we used the valuation set of Zimbabwe based on crosswalk function to calculate utility,[25] as its Gross Domestic Product per capita in PPP US$ (2,381.22) was the closest to that of the two countries of interest (Bangladesh: 4,598.39; Pakistan: 5,714.03) at the time of the analysis.[26] QALYs were derived using the area under the curve approach.[27]

Analyses

All analyses were performed using STATA 16.0 SE.

Missing data

For the baseline covariates, missing values were imputed by the mean of the variable in the pooled sample in the same country. This was the information that was unrelated to the intervention and the randomisation functioned to balance the two arms.[28] The missing values in the follow-up variables were handled using multiple imputation method, following Rubin's rule and assuming missing at random (MAR),[29] unless it was due to death. Missing values due to death were replaced with zero or not applicable (n/a) depending on the nature of variable. An imputation model was developed to include all the variables necessary for the analysis and the number of imputations was set as approximately the highest percentage figure of missing data.[30] The imputation was performed by trial arms and on condition of being alive.

Primary analysis

The primary analysis was an incremental cost-utility analysis over six months post-randomisation from a public or voluntary healthcare sector perspective. This included service providers that were classified as government, non-profit organisations, and charitable organisations. It was undertaken on an intention-to-treat basis, including all randomised participants in the arms to which they were allocated.

Total costs at 6 months consisted of intervention costs, TB treatment costs, additional public/voluntary smoking cessation costs, and public/voluntary healthcare services costs in the six months post-randomisation. Mean total costs and mean QALYs were estimated for each arm and no discounting was applied for the six months period. Incremental mean total costs and incremental mean QALYs were estimated by a mixed effect generalised linear regression model, adjusting for their respective baseline values (total costs in the six months before randomisation for total costs; baseline EQ-5D-5L utility for QALYs), age, gender, country, with study site as random-effects. An incremental cost-effectiveness ratio (ICER) was calculated by dividing the incremental mean total costs by the incremental mean QALYs.

Since there are no official willingness-to-pay (WTP) thresholds in either Bangladesh or Pakistan, the estimated WTPs for Bangladesh and Pakistan based on income elasticity of value of health, inflated to 2018 (maximum WTP: Bangladesh: PPP US$1,473 per QALY gained; Pakistan: PPP US$2,431 per QALY gained), were used to compare with the ICERs, if applicable.[31]

Because neither costs nor QALYs were normally distributed, we used a non-parametric bootstrap technique to assess the uncertainty, generating 5000 replicate samples. The results were used to construct 95% confidence intervals (CIs) of the incremental costs and QALYs. They were then plotted on a cost-effectiveness plane (CEP) to demonstrate the uncertainty surrounding the ICER. Cost-effectiveness acceptability curves (CEACs) were constructed from these bootstrapped replicates by converting ICER to net monetary benefit.[32]

For peer review only - http://bmjopen.bmj.com/site/about/guidelines.xhtml

A separate cost-effectiveness analysis using smoking abstinence rate at six months follow-up as effect measure was planned but not undertaken because no statistically significant difference was found between arms for this outcome measure per pre-specified effect size.[10] Given that it is not clinically effective, it could not be cost-effective using this measure.

Sensitivity analyses

We undertook a complete case analysis (CCA) on the participants who had complete outcome and covariates data to provide a comparison with the primary analysis based on imputed data. We examined the MAR assumption that supports the multiple imputation by undertaking sensitivity analyses based on missing not at random (MNAR) assumptions using a practical approximation to the pattern mixture model:[28] (1) imputed total costs were increased by 10%, 20% and 30%; (2) imputed QALYs were reduced by between 10%, 20%, and 30%. To assess the impact of choice of EQ-5D-5L tariff, we took the validated population valuation sets from countries in the southeast Asia area (i.e. Indonesia, Malaysia, Thailand) and the crosswalk functions of the UK and Thailand to calculate utility for comparison.[25, 33-35]

Secondary analyses

The first secondary analysis followed the methods of the primary analysis, extending time horizon to a 12-month period. No discounting was applied as this was not longer than one year. We summarised participants' OOPs in relation to TB treatment, smoking cessation, and healthcare services by arm, at both 6 and 12 months. Productivity losses of participants' sick leave and their companion to treatment, and money spent on any forms of tobacco were also summarised. We have also repeated the analysis by countries following the same methods of the primary analysis above.

PATIENT AND PUBLIC INVOLVEMENT

Patient groups were consulted on the intervention materials for their lucidness during the intervention development stage. No other patient and public involvement occurred in the study process.

RESULTS

Missing data

The results of observed cases are presented in Supplementary file 1. The proportion of missing data at baseline was low (Supplementary file 3 Table S2). The greatest percentage of missing data level was 12% of participants' OOPs for smoking cessation at 6 months follow-up, followed by the same variable at 12 months (10%).

Although missing data did not differentiate between arms, most of the missingness of follow-up variables was significantly associated with country. The missingness of OOP for smoking cessation in months 1-6 was weakly associated with participants' age (Supplementary file 3 Table S3). Using a logistic regression for missingness of follow-up variables on their respective previously observed values (e.g. missingness of costs at six months on costs at baseline), most results were not statistically significant (p>0.05), with few exceptions. These results supported the MAR assumption. The imputation number was set to 15.

For peer review only - http://bmjopen.bmj.com/site/about/guidelines.xhtml

Primary analysis

The mean costs of smoking cessation and healthcare services in the six months before baseline were PPP US$10.36 (SE PPP US$1.74) in the cytisine arm and PPP US$8.52 (SE PPP US$1.41) in the placebo arm. The mean total costs over the six months post-randomisation were PPP US$401.52 (SE PPPUS$8.91) in the cytisine arm and PPP US$334.73 (SE PPP US$5.85) in the placebo arm (Table 2). Costs of additional smoking cessation were negligible in both arms. The mean costs of hospital stay in the cytisine arm were almost twice those in the placebo arm. The incremental total costs were PPP US$57.74 (95% CI PPP US$49.40 to PPP US$83.36). The mean QALYs were 0.395 (SE 0.002) in the cytisine arm and 0.398 (SE 0.002) in the placebo arm. The incremental QALYs were -0.001 (95% CI -0.004 to 0.002). The majority (78.1%, 3905/5000) of the bootstrapped replicates fell in the north-west quadrant of CEP, indicating a more costly, but less effective intervention (Figure 1 left). The CEAC was not presented as it was a straight line at 0% probability of cost-effectiveness at the WTP range from PPP US$0 to PPP US$1,473 per QALY gained for Bangladesh or PPP US$2,431 per QALY gained for Pakistan.

[Insert Table 2 here]

[Insert Figure 1 here]

Sensitivity analyses

The CCA was performed on 1122 participants in the cytisine arm and 1116 participants in the placebo arm. The results were similar to that of the primary analysis (Table 2 right). The overall majority (91%, 4550/5000) of the bootstrapped replicates fell in the north-west quadrant of CEP (Figure 1 right), indicating a more costly, but less effective intervention. This was consistent with the primary analysis.

Under scenario (1), when the imputed costs were increased by 10%, 20% and 30%, the incremental costs became PPP US$58.32, PPP US$58.91 and PPP US$59.51, respectively. Under scenario (2), when the imputed QALYs were reduced by 10%, 20% and 30%, the incremental QALYs were -0.001, -0.001 and -0.000, respectively. None differed far from the primary analysis results.

Using tariffs derived in different countries or with different approaches, the incremental QALYs between arms varied (Figure 2), but the level of difference was not prominent and the general pattern between arms remained the same.

[Insert Figure 2 here]

Secondary analyses

The addition of the costs in months 7-12 increased the mean total costs over 12 months to PPP US$408.31 (SE PPP US$10.03) in the cytisine arm and PPP US$341.83 (SE PPP US$6.50) in the placebo arm. The incremental costs were PPP US$56.72 (95% CI PPP US$46.58 to PPP US$86.00), similar to those over the six months post randomisation. By contrast, as the time horizon doubled, the QALYs became almost twice as high as over the six-month period, which led to a larger difference in mean QALYs between arms. The mean QALYs were 0.808 (SE 0.004) in the cytisine arm and 0.814 (SE 0.004) in the placebo arm. The incremental QALYs were -0.004 (95% CI -0.013 to 0.005). The cytisine arm remained dominated by the placebo arm, with 77% (4007/5000) of the bootstrapped estimates indicating a less effective, but more costly intervention.

For peer review only - http://bmjopen.bmj.com/site/about/guidelines.xhtml

Over the 12 months follow-up period, the mean OOPs were PPP US$108.91 (SE PPP US$19.79) in the cytisine arm and PPP US$81.74 (SE PPP US$11.73) in the placebo arm. The main cost driver was OOP for doctor visits in both arms, while in the cytisine arm participants also spent more on hospital stays (Table 3). This pattern was consistent with costs from the public or voluntary healthcare sector's perspective. Productivity losses mostly occurred before and during TB treatment period and decreased considerably in the last six months of the trial. The OOP for tobacco products dropped after randomisation in both arms but remained stable throughout the 12 months period post-randomisation, which was consistent with the quit rates observed in both arms.

[Insert Table 3 here]

By country analyses did not lead to different conclusions from the primary analysis. In Bangladesh, the adjusted incremental costs were PPP US$37.06 (95% CI PPP US$28.12 to PPP US$43.85) and the adjusted incremental QALYs were -0.003 (95% CI -0.006 to 0.000), with the cytisine arm remaining dominated by the placebo arm. In Pakistan, the adjusted incremental costs were PPP US$108.46 (95% CI PPP US$69.69 to PPP US$157.88) and the adjusted incremental QALYs were 0.001 (95% CI -0.004 to 0.008). The ICER was calculated at PPP US$108,464 per QALY, which was much higher than the adopted maximum WTP threshold PPP US$2,431 per QALY. The cost-effectiveness plane also shows that cytisine plus BS had 0% of being cost-effective within the adopted WTP threshold range in both countries (Supplementary file 1). However, the breakdown of total costs by country indicated that the higher mean costs of hospital stay in the cytisine arm were mostly contributed by the cytisine arm in Pakistan (PPP US$78.12 vs PPP US$32.70 in placebo arm). While in Bangladesh, the mean costs of hospital stay were PPP US$3.07 (SE PPP US$1.62) in the cytisine arm and PPP US$7.34 (SE PPP US$3.82) in the placebo arm. A further examination also showed possible outliers in the cytisine arm in Pakistan. The improvement in utility from baseline to six months was more manifest in Bangladesh than in Pakistan, regardless of the arms. Detailed results are presented in Supplementary file 1.

DISCUSSION

The intervention cost was PPP US$60.65 (SE PPP US$0.41) per participant in the cytisine arm and PPP US$12.37 (SE PPP US$0.08) per participant in the placebo arm. The difference was mainly attributed to cytisine medication. The incremental total costs at six months post-randomisation were estimated at PPP US$57.74 (95% CI PPP US$49.40 to PPP US$83.36) while the incremental QALYs were estimated at -0.001 (95% CI -0.004 to 0.002). These results indicated that adding cytisine to brief BS for quitting smoking was unlikely to be cost-effective. The sensitivity analyses confirmed the robustness of this conclusion. Extending the time horizon to 12 months did not change the conclusion.

While the observed quit rates were not statistically significantly different between arms, [10] participants' OOP for tobacco products on average dropped by nearly two-thirds after randomisation, indicating a reduction of tobacco consumption. The higher than expected productivity loss, OOPs for doctor visits and TB treatment before baseline might be because participants had experienced some symptoms and sought medical attention before TB was diagnosed. It was unclear, however, why participants in the cytisine arm reported more and longer hospital stays than the placebo arm in Pakistan. Our process evaluation study found some difference in intervention delivery between countries,[36, 37] but we did not find evidence of differential TB treatment outcomes between trial arms in Pakistan, [10] and the same situation was not observed in Bangladesh. This might indicate a potential country-related contextual reason rather than the effect

For peer review only - http://bmjopen.bmj.com/site/about/guidelines.xhtml

of the intervention, or occurrence by chance. Subgroup analyses by patient characteristics and deterministic sensitivity analysis of key parameters were not planned because of the lack of clear underlying hypotheses. Moreover, limited by the research capacity, the sample size of the subgroups was likely to be insufficient to produce valid results.

The strength of the study stems from the large sample size and high follow-up rates. Despite limitations of published data availability, patient level measures were collected using a comprehensive questionnaire to enable a full cost-utility analysis to be undertaken. However, several limitations could potentially affect the results. Firstly, our estimated costs could be an underestimation. We observed that some health workers discussed smoking cessation during several routine TB consultations and some research assistants delivered the study drug to participants if they had missed day 5 follow-up. TB treatment costs were estimated based on simplified scenarios. Intensive treatments in the case of deterioration, death or retreatment were not considered. Costs of general medication were not included because our unit costs data source for healthcare services did not include them. However, this should not bias the results towards either arm. Secondly, the data source of unit costs of healthcare services was last updated in 2010. Certain changes may not be accounted for by simple inflation. While an up-to-date data source was not available at the time of analysis, the results could be updated when it becomes available as the service use was collected in quantities. Thirdly, productivity loss in the case of death was considered zero but if a life-time observation or modelling were undertaken, productivity loss due to premature death should be included. Given the large sample size and few deaths that occurred, this was unlikely to affect the conclusions. Last but not least, our sample consisted mostly of men. This reflected the low daily tobacco smoking rate among women in both countries at the time of the trial (0.8% in Bangladesh, 2.0% in Pakistan). [5] There may therefore be challenges in making inferences to women in these countries.

To our knowledge, this is the first cost-utility study of cytisine as a smoking cessation aid alongside an RCT and one of few for smoking cessation intervention in LMICs. A systematic review published in 2019 identified eight placebo-controlled trials and one non-inferiority trial (using nicotine replacement therapies) that used cytisine for smoking cessation, all of which were among smokers in general population and only one was conducted in LMICs.[8] Although cytisine has been identified as affordable globally [38] its cost-effectiveness in smoking cessation was based on modelled economic evaluation not empirical evidence.[39] Our study illustrated that though less costly than other cessation aids, cytisine did not show sufficient effects to be considered cost-effective.

Our findings do not support the cost-effectiveness of adding cytisine to BS for smokers who are newly diagnosed with pulmonary TB. In the absence of more effective smoking cessation aid, future studies should explore the cost-effectiveness of non-pharmacological cessation interventions in LMICs, given the relatively lower costs of labour, and possible impact of smoking-related comorbidities on quality of life in the TB population.

CONTRIBUTORS

JL conducted the cost-effectiveness analysis and drafted the manuscript under the supervision of SP. SP also contributed to the analysis design. AKe contributed to data management and statistical analysis, including some clinical measures used in this manuscript. OD and RG contributed to study design, conduct and interpretation of findings. AR and AMM managed the study and contributed to interpretation of findings. RH, DB, RF, AK, RZ and SM conducted the study in Bangladesh/Pakistan, collected and managed the data in countries and provided critical inputs to data analysis and

For peer review only - http://bmjopen.bmj.com/site/about/guidelines.xhtml

interpretation. DK, EK, MB and HE provided insights to study design on aspects of behavioural support implementation, evaluation of its delivery and interpretation of findings. AS provided critical oversight to study design, trial conduct, interpretation of findings and discussion. KS conceptualised the study, contributed to the study design, conduct, and interpretation of findings.

All authors provided critical revisions and approved the final manuscript.

COMPETING INTERESTS

Kamran Siddiqi received a research grant from Pfizer (2015-2017) to study the effects of varenicline (a smoking cessation medication) on waterpipe smoking cessation. Daniel Kotz received an unrestricted grant from Pfizer in 2009 for an investigator-initiated trial on the effectiveness of practice nurse counselling and varenicline for smoking cessation in primary care (Dutch Trial Register NTR3067; DOI: 10.1111/add.13927). The medication for the trial were provided by Aflofarm free of charge. Aziz Sheikh is supported by Health Data Research UK's BREATHE Hub.

FUNDING

This work was supported by the European Union's Horizon 2020 research and innovation programme, under Grant Agreement No. 680995. The European Commission is not responsible for any of the content of this article. The manufacturer, Aflofarm, supplied trial medication at no cost.

DATA AVAILABILITY STATEMENT

Access to partial anonymised datasets from the study can be provided upon request to KS (Chief Investigator) through corresponding author of this manuscript. Statistical code is available on GitHub (Clearice84/TBTobacco: Health economic analysis STATA code for TB&Tobacco trial (github.com)).

ETHICS STATEMENT

Ethical approval was granted by the Health Sciences Research Governance Committee (HSRGC) at the University of York, UK (HSRGC/2016/144/B), the National Bioethics Committee, Pakistan Medical Research Council (no. 4–87/16/NBC-200 Part-B/RDC/4197) and the National Research Ethics Committee, Bangladesh Medical Research Council (BMRC/NREC/2016–2019/1475).

For peer review only - http://bmjopen.bmj.com/site/about/guidelines.xhtml

REFERENCES

1. World Health Organization. Global tuberculosis report 2021. Geneva; 2021 14 October. https://www.who.int/publications/i/item/9789240037021.
2. TB country, regional and global profiles [Internet]. World Health Organization. 2021 [cited 01/12/2021]. Available from: https://worldhealthorg.shinyapps.io/tb_profiles/.
3. World Health Organization. WHO global report on trends in prevalence of tobacco use 2000-2025. Geneva; 2021 16 November. https://www.who.int/publications/i/item/9789240039322.
4. Chaves Torres NM, Quijano Rodriguez JJ, Porras Andrade PS, et al. Factors predictive of the success of tuberculosis treatment: A systematic review with meta-analysis. *PloS one*. 2019;14(12):e0226507. https://www.ncbi.nlm.nih.gov/pubmed/31881023.
5. World Health Organization (WHO). Tobacco control profiles - countries, territories, and areas: Tobacco Free Initiative; 2019 [cited 2020 6th July]. https://www.who.int/tobacco/surveillance/policy/country_profile/en/#P.
6. Lawson PJ, Flocke SA. Teachable moments for health behavior change: a concept analysis. *Patient Educ Couns*. 2009;76(1):25-30. https://www.ncbi.nlm.nih.gov/pubmed/19110395.
7. Elsey H, Warsi S. Report of TB and Tobacco Project WP1: tobacco cessation intervention development [Internet]2017 01/12/2021 [cited 2021 1 December]. https://tbandtobacco.org/wp-content/uploads/sites/26/2019/05/WP1-Report-of-intervention-development.pdf.
8. Tutka P, Vinnikov D, Courtney RJ, et al. Cytisine for nicotine addiction treatment: a review of pharmacology, therapeutics and an update of clinical trial evidence for smoking cessation. *Addiction*. 2019;114(11):1951-69. https://www.ncbi.nlm.nih.gov/pubmed/31240783.
9. Dogar O, Barua D, Boeckmann M, et al. The safety, effectiveness and cost-effectiveness of cytisine in achieving six-month continuous smoking abstinence in tuberculosis patients-protocol for a double-blind, placebo-controlled randomized trial. *Addiction*. 2018. https://www.ncbi.nlm.nih.gov/pubmed/29676824.
10. Dogar O, Keding A, Gabe R, et al. Cytisine for smoking cessation in patients with tuberculosis: a multicentre, randomised, double-blind, placebo-controlled phase 3 trial. *Lancet Glob Health*. 2020;8(11):e1408-e17. https://www.ncbi.nlm.nih.gov/pubmed/33069301.
11. Prices, Consumer Price Index, All items, Index [Internet]. International Monetary Fund (IMF). 2019 [cited 08/05/2019]. Available from: http://data.imf.org/regular.aspx?key=61545861 Archived at http://www.webcitation.org/78DlAn9Cu.
12. DataBank World Development Indicators [Internet]. 2019 [cited 26/06/2019]. Available from: https://databank.worldbank.org/reports.aspx?source=2&series=PA.NUS.PPP&country=PAK#.
13. Global Tuberculosis report 2018 - TB data [Internet]. WHO. 2018 [cited 20/05/2019]. Available from: https://www.who.int/tb/country/data/download/en/ Archived at: http://www.webcitation.org/78VxUbGR1.
14. Gomez GB, Dowdy DW, Bastos ML, et al. Cost and cost-effectiveness of tuberculosis treatment shortening: a model-based analysis. *BMC Infect Dis*. 2016;16(1):726. https://www.ncbi.nlm.nih.gov/pubmed/27905897.
15. National Currency per U.S. Dollar, period average [Internet]. International Monetary Fund (IMF). 2019 [cited 08/05/2019]. Available from: http://data.imf.org/regular.aspx?key=61545862 Archived at http://www.webcitation.org/78DkMANRB.
16. World Health Organization. Tobacco control country profile Pakistan. Initiative TF; 2017. https://www.who.int/tobacco/surveillance/policy/country_profile/pak.pdf?ua=1.
17. World Health Organization. Tobacco control country profile Bangladesh. Initiative TF; 2017. https://www.who.int/tobacco/surveillance/policy/country_profile/bgd.pdf?ua=1.
18. National Institute for Health and Care Excellence. Putting NICE guidance into practice - Resource impact report: Stop smoking interventions and services (NG92). 2018 March. Available at: https://www.nice.org.uk/guidance/ng92/resources/resource-impact-report-pdf-4788823645 Archived at: http://www.webcitation.org/766hqbhTb.

For peer review only - http://bmjopen.bmj.com/site/about/guidelines.xhtml

19. Curtis LA, Burns A. Unit Costs of Health and Social Care 2018. Project report. Kent: University of Kent; 2018 14th January. Available at: https://kar.kent.ac.uk/70995/1/Unit%20Costs%202018%20-%20FINAL%20with%20bookmarks%20and%20covers.pdf Archived at: http://www.webcitation.org/75n7YG9xI.
20. Bangladesh Bureau of Statistics. Report on Labour Force Survey (LFS) 2016-17. Statistics, Informatics Division MoP; 2018 January. https://www.ilo.org/surveydata/index.php/catalog/2047/download/16818.
21. Pakistan Bureau of Statistics. Labour Force Survey 2017-18. Ministry of Statistics GoP; 2018 December. http://www.pbs.gov.pk/sites/default/files//Labour%20Force/publications/lfs2017_18/Annual%20Report%20of%20LFS%202017-18.pdf.
22. Economic Analysis Evaluation Team. Country-specific inpatient and outpatient estimates in 2010 currency. World Health Organisation, Department of Health Systems G, Financing; 2010. https://www.who.int/choice/cost-effectiveness/inputs/country_inpatient_outpatient_2010.pdf?ua=1.
23. International Labour Office. International Standard Classification of Occupations: ISCO-08. Geneva: ILO; 2012.
24. EuroQol Research Foundation. EQ-5D-5L User Guide: Basic information on how to use the EQ-5D-5L instrument (version 3.0)2019 17/06/2020. Available at: https://euroqol.org/publications/user-guides/.
25. van Hout B, Janssen MF, Feng YS, et al. Interim scoring for the EQ-5D-5L: mapping the EQ-5D-5L to EQ-5D-3L value sets. *Value Health*. 2012;15(5):708-15. https://www.ncbi.nlm.nih.gov/pubmed/22867780.
26. IMF DataMapper - GDP per capita, current prices (Purchasing power parity; international dollars per capita) [Internet]. International Monetary Fund (IMF). 2018 [cited 4th April 2019]. Available from: https://www.imf.org/external/datamapper/PPPPC@WEO/BGD/PAK/ZWE/GBR/THA/MYS/IDN/SEQ?year=2018.
27. Richardson G, Manca A. Calculation of quality adjusted life years in the published literature: a review of methodology and transparency. *Health Econ*. 2004;13(12):1203-10. https://www.ncbi.nlm.nih.gov/pubmed/15386669.
28. Faria R, Gomes M, Epstein D, et al. A guide to handling missing data in cost-effectiveness analysis conducted within randomised controlled trials. *PharmacoEconomics*. 2014;32(12):1157-70. https://www.ncbi.nlm.nih.gov/pubmed/25069632.
29. Rubin DB. Statistical Matching Using File Concatenation with Adjusted Weights and Multiple Imputations. *J Bus Econ Stat*. 1986;4(1):87-94. <Go to ISI>://WOS:A1986AXF5400013.
30. White IR, Royston P, Wood AM. Multiple imputation using chained equations: Issues and guidance for practice. *Stat Med*. 2011;30(4):377-99. https://www.ncbi.nlm.nih.gov/pubmed/21225900.
31. Woods B, Revill P, Sculpher M, et al. Country-Level Cost-Effectiveness Thresholds: Initial Estimates and the Need for Further Research. *Value Health*. 2016;19(8):929-35. https://www.ncbi.nlm.nih.gov/pubmed/27987642.
32. Fenwick E, Claxton K, Sculpher M. Representing uncertainty: the role of cost-effectiveness acceptability curves. *Health Econ*. 2001;10(8):779-87. https://www.ncbi.nlm.nih.gov/pubmed/11747057.
33. Pattanaphesaj J, Thavorncharoensap M, Ramos-Goni JM, et al. The EQ-5D-5L Valuation study in Thailand. *Expert Rev Pharmacoecon Outcomes Res*. 2018;18(5):551-8. https://www.ncbi.nlm.nih.gov/pubmed/29958008.
34. Purba FD, Hunfeld JAM, Iskandarsyah A, et al. The Indonesian EQ-5D-5L Value Set. *PharmacoEconomics*. 2017;35(11):1153-65. https://www.ncbi.nlm.nih.gov/pubmed/28695543.

For peer review only - http://bmjopen.bmj.com/site/about/guidelines.xhtml

35. Shafie AA, Vasan Thakumar A, Lim CJ, et al. EQ-5D-5L Valuation for the Malaysian Population. *PharmacoEconomics*. 2019;37(5):715-25. https://www.ncbi.nlm.nih.gov/pubmed/30535779.
36. Boeckmann M, Warsi S, Noor M, et al. Health worker and patient views on implementation of smoking cessation in routine tuberculosis care. *NPJ Prim Care Respir Med*. 2019;29(1):34. https://www.ncbi.nlm.nih.gov/pubmed/31481678.
37. Boeckmann M, Dogar O, Saeed S, et al. Measuring fidelity to delivery of a new smoking cessation intervention integrated into routine tuberculosis care in Pakistan and Bangladesh: Contextual differences and opportunities. *Tob Induc Dis*. 2021;19:24. https://www.ncbi.nlm.nih.gov/pubmed/33841063.
38. West R, Raw M, McNeill A, et al. Health-care interventions to promote and assist tobacco cessation: a review of efficacy, effectiveness and affordability for use in national guideline development. *Addiction*. 2015;110(9):1388-403. https://www.ncbi.nlm.nih.gov/pubmed/26031929.
39. Leaviss J, Sullivan W, Ren S, et al. What is the clinical effectiveness and cost-effectiveness of cytisine compared with varenicline for smoking cessation? A systematic review and economic evaluation. *Health Technol Assess*. 2014;18(33):1-120. https://www.ncbi.nlm.nih.gov/pubmed/24831822.

For peer review only

For peer review only - http://bmjopen.bmj.com/site/about/guidelines.xhtml

Figure 1: Cost-effectiveness plane of primary and complete case analyses at six months post-randomisation (dashed purple line as WTP for Pakistan, dotted green line as WTP for Bangladesh)

Figure 2: Comparison of adjusted incremental QALYs over six months post-randomisation derived from different methods

Table 1 Unit costs of TB treatment, smoking cessation services and healthcare services

**Cost items**	**Unit Cost (PPP US$, 2017/18)**		**Sources**
	**Bangladesh**	**Pakistan**	
**TB treatment**			
First-line treatment, intensive phase, including drugs	54.21 per month	108.40 per month	[12-15]
First-line treatment, continuation phase, including drugs	31.62 per month	63.24 per month	
**Smoking cessation services**			
Help or advice from public/government clinic/hospital	0.68/use	0.89/use	[12, 19-21]
Group or single counselling session at public/voluntary clinic	0.94/session	1.26/session	[12, 18, 20, 21]
**General healthcare services**			
Doctor visit	4.60/visit	6.83/visit	[11, 12, 22]
Hospital inpatient	19.06/bed-day	33.14/bed-day	[11, 12, 22]

Table 2 Results of primary and complete cases analyses at six months post-randomisation

	**Primary analysis**		**Complete case analysis**	
	**Cytisine (n=1239)**	**Placebo (n=1233)**	**Cytisine (n=1122)**	**Placebo (n=1116)**
**Costs (PPP US$)**	**Mean (SE)**		**Mean (SD)**	
Intervention	60.65 (0.41)	12.37 (0.08)	61.25 (13.83)	12.15 (2.69)
TB treatment	305.15 (3.36)	301.83 (3.36)	306.53 (109.96)	301.36 (108.09)
Doctor visit	3.36 (0.37)	3.10 (0.31)	3.47 (13.17)	3.14 (10.58)
Hospital stay	31.91 (7.73)	16.98 (4.41)	33.08 (275.18)	17.26 (151.58)
Smoking cessation	0.46 (0.03)	0.45 (0.03)	0.49 (1.19)	0.49 (1.13)
Overall total for six months	401.52 (8.91)	334.73 (5.85)	404.82 (311.99)	334.39 (196.52)
**PPP US$, Mean (95% CI)**				
Adjusted incremental costs	57.74 (49.40 to 83.36)		59.49 (51.95 to 89.30)	
	Mean (SE)		Mean (SD)	
QALYs over six months	0.395 (0.002)	0.398 (0.002)	0.401 (0.041)	0.403 (0.039)
**QALYs, Mean (95% CI)**				
Adjusted incremental QALYs	-0.001 (-0.004 to 0.002)		-0.001 (-0.003 to 0.000)	

For peer review only - http://bmjopen.bmj.com/site/about/guidelines.xhtml

CEA of cytisine for smoking cessation in TB

ICER	Cytisine dominated by placebo (uncertainty see Figure 1 left)	Cytisine dominated by placebo (uncertainty see Figure 1 right)

Table 3 Mean Out-of-pocket payments for health-related services, productivity loss and payments for tobacco products at three time points, by arm

**PPP US$ Mean (SE)**	**Cytisine (n=1239)**	**Placebo (n=1233)**
**Six months before baseline**		
OOPs for health-related services	84.90 (7.91)	86.70 (6.80)
TB treatment	15.60 (1.69)	19.71 (3.42)
Doctor visit	62.29 (6.90)	63.96 (5.67)
Hospital stay	6.97 (2.87)	3.02 (0.80)
Smoking cessation	0.04 (0.02)	0.01 (0.01)
Productivity loss	34.01 (2.14)	30.41 (1.81)
OOPs for tobacco products	1.79 (0.14)	1.64 (0.07)
**Months 1 – 6**		
OOPs for health-related services	69.70 (10.62)	51.08 (9.32)
TB treatment	22.16 (2.51)	16.24 (1.30)
Doctor visit	29.49 (7.52)	22.65 (6.08)
Hospital stay	17.65 (5.90)	11.89 (6.53)
Smoking cessation	0.40 (0.09)	0.30 (0.06)
Productivity loss	48.83 (3.00)	43.52 (3.14)
OOPs for tobacco products	0.51 (0.03)	0.50 (0.03)
**Months 7 – 12**		
OOPs for health-related services	39.21 (16.11)	30.66 (6.72)
TB treatment	5.03 (1.43)	4.55 (0.92)
Doctor visit	13.05 (2.41)	20.42 (5.22)
Hospital stay	21.08 (15.80)	5.64 (2.89)
Smoking cessation	0.04 (0.02)	0.05 (0.02)
Productivity loss	6.06 (0.58)	8.32 (0.97)
OOPs for tobacco products	0.61 (0.03)	0.58 (0.02)

For peer review only - http://bmjopen.bmj.com/site/about/guidelines.xhtml

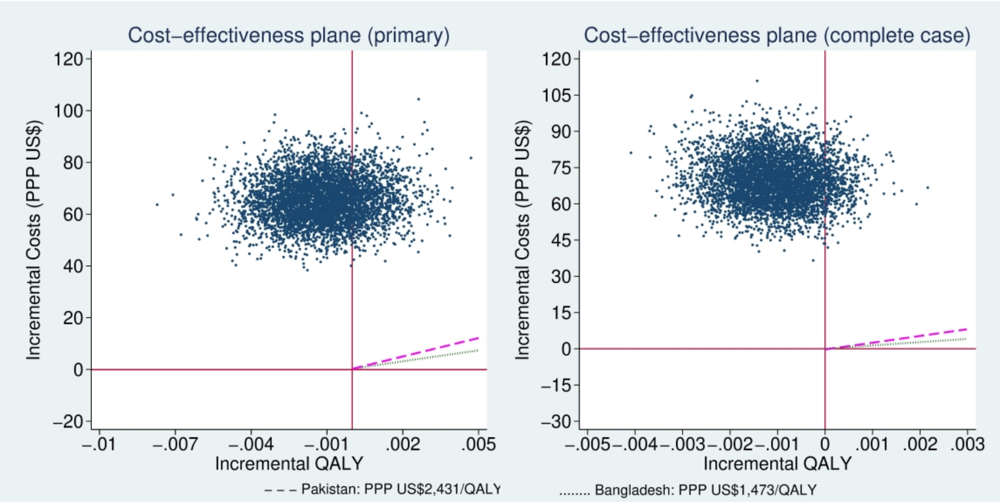

Cost-effectiveness plane of primary and complete case analyses at six months post-randomisation

254x127mm (600 x 600 DPI)

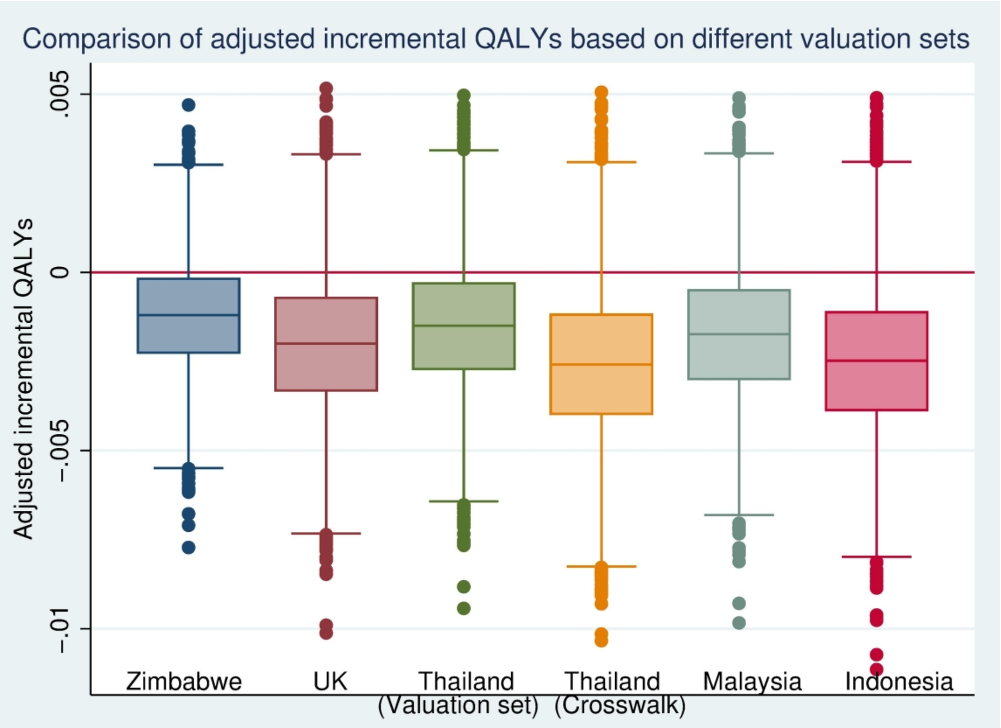

Comparison of adjusted incremental QALYs over six months post-randomisation derived from different methods

139x101mm (600 x 600 DPI)

Detailed methods and results of secondary analyses

Methods

Cytisine dosage schedule

The standard regimen for cytisine (Desmoxan, Aflofarm) was a 25-day course with 1.5mg hard capsules for oral administration, with six per day on days 1-3, five per day on days 4-12, four per day on days 13-16, three per day on days 17-20, two per day on days 21-24 and one on the last day.

Intervention costs

Training for the delivery of brief behavioural support was given to TB health workers before the trial began. In Bangladesh, it was a one-day training programme with a one-day refresher training and the total cost was estimated to be €4499 in 2017. In Pakistan, this consisted of a two-day training programme for DOTS facilitators and the total cost was estimated at €2324 in 2016.

In Bangladesh, the average monthly salary of a TB health worker (local salary grades G-11 to G-13) was PPP US$649.84, and average working hours per week was 48 hours.[1] In Pakistan, the average monthly salary of a TB health worker was PPP US$921.50 and average working hours per week was 47.4 hours.[2] We assumed a 30-day month as 4.3 weeks. The estimated hourly wage was therefore PPP US$3.17 in Bangladesh and PPP US$4.54 in Pakistan. The cost of BS was PPP US$0.52 for the first session and PPP US$0.26 for the second session in Bangladesh and PPP US$0.75 and PPP US$0.38 in Pakistan.

TB treatment costs

The standard treatment for pulmonary TB consisted of a two-month intensive phase and a four-month continuation phase. We extracted the overall costs of a six-month TB treatment for the two countries from the World Health Organization (WHO) TB database[3] and applied a ratio of costs of the two phases, based on a TB treatment modelling study,[4] to produce an estimate of monthly cost of intensive phase and continuation phase respectively. They were then converted to PPP US$.[5,6] The TB treatment costs were then estimated based on the participants' treatment progression on their TB registry cards.

Smoking cessation costs outside of the trial

Due to the limited smoking cessation services in the two countries,[7,8] we made assumptions on duration, based on usual practice in the UK:[9,10] 10-minute brief intervention with professionals (physician or professional nurse) for help/advice from a public/government clinic/hospital; one hour group session of 15 people or 30-minute individual session led by medical technicians/auxiliary nurses for counselling sessions in public/voluntary hospital. The ratio of group and individual sessions was assumed to be 1:1. The average hourly wage was PPP US$4.14 for "professionals" and PPP US$3.33 for "technicians and associate professionals" in Bangladesh, and PPP US$5.29 for "professionals" and PPP US$4.51 for "technicians and associate professionals" in Pakistan.[1,2,5]

General healthcare services costs

Participants' visits to a public/voluntary doctor and length of stay in a public hospital in the previous six months were collected by self-report at baseline, 6- and 12-month follow-ups. The unit costs of these services were extracted from the WHO country specific in- and out-patient costs, inflated to 2018 and converted to PPP US$.[5,11,12] The unit cost of hospital inpatient stay was the average of all hospital levels and the unit cost of a visit to doctor was the average of all settings for outpatient. These costs did not include drugs.

For peer review only

Out-of-pocket payments (OOPs)

Participants' spending related to following items were collected: TB treatment, public/voluntary doctor and hospital visits, and private doctor and hospital visits, including travel, smoking cessation services in public/voluntary facilities and private settings, purchasing Nicotine Replacement Therapy (NRT) or e-cigarette refills, purchasing other traditional medicine for quitting, and purchasing tobacco products.

Results

Costs

Mean training costs were PPP US$10.94 (SD PPP US$2.09) per participant in the cytisine arm and PPP US$10.92 (SD PPP US$2.09) per participant in the placebo arm. Mean cost of the information leaflet was PPP US$0.76 (SD PPP US$0.75) in the cytisine arm and PPP US$0.75 (SD PPP US$0.75) in the placebo arm. Mean cost of BS was PPP US$0.68 (SD PPP US$0.36) among 1233 participants in the cytisine arm and PPP US$0.70 (SD US$0.36) among 1226 participants in the placebo arm. Mean cost of cytisine was PPP US$48.27 (SD PPP US$12.54) while the cost of placebo was assumed at zero.

Mean costs of TB treatment were estimated to be PPP US$307.39 (SD PPP US$110.25) in the cytisine arm and PPP US$302.45 (SD PPP US$108.53) in the placebo arm, excluding 102 (8.2%) participants in the cytisine arm and 103 (8.4%) in the placebo arm who did not have information from TB cards at six-month follow-up (Table 1). The use of smoking cessation support was reported by a small group of participants in both arms. Mean costs of public/voluntary smoking cessation services were low in both arms throughout the 12 months period. Most participants reported neither visiting a doctor other than for their TB treatment nor being admitted to hospital for any reason. While mean costs of doctor visits were similar between respondents in both arms throughout the trial period, mean costs of hospital stay in the cytisine arm were nearly twice as high as in the placebo arm in months 1-6.

Out-of-pocket payments

The respondents reported an increase of spending on smoking cessation in months 1-6 compared to close to none before and after, corresponding with the intervention delivery and TB treatment period. Mean spending on tobacco was lower during the trial period than before among respondents. However, in comparison with the spending on smoking cessation, the spending on tobacco was consistently higher. The OOPs for healthcare services, including travel, loosely followed the same pattern of the costs of the services (Table 1).

Table 1 Mean (SD) costs and OOPs of TB treatment, additional smoking cessation services and general healthcare services, and OOPs on tobacco products, by arm

	Cytisine (n=1239)		Placebo (n=1233)	
	**n**	**Mean (SD) PPP US$**	**n**	**Mean (SD) PPP US$**
**TB treatment costs**				
TB registry	1137	307.39 (110.25)	1130	302.45 (108.53)
**Additional smoking cessation costs**				
Six months before baseline	1239	0.00 (0.10)	1233	0.00 (0.09)
Months 1-6	1174	0.47 (1.17)	1164	0.47 (1.11)
Months 7-12	1134	0.22 (0.75)	1144	0.21 (0.77)
**Doctor visit costs**				
Six months before baseline	1239	3.26 (14.27)	1232	3.48 (23.44)
Months 1-6	1176	3.39 (12.96)	1166	3.04 (10.39)

For peer review only

Months 7-12	1148	1.27 (4.73)	1157	1.12 (4.58)
**Hospital stay costs**				
Six months before baseline	1237	6.77 (57.79)	1231	4.84 (43.25)
Months 1-6	1175	31.58 (268.99)	1166	16.52 (148.33)
Months 7-12	1148	5.01 (80.30)	1157	5.87 (94.86)
**Additional smoking cessation OOPs**				
Six months before baseline	1236	0.04 (0.75)	1230	0.00 (0.09)
Months 1-6	1091	0.34 (2.72)	1080	0.28 (1.95)
Months 7-12	1110	0.05 (0.61)	1115	0.05 (0.56)
**Tobacco OOPs**				
Six months before baseline	1229	1.79 (5.05)	1224	1.64 (2.35)
Months 1-6	1177	0.50 (1.03)	1166	0.48 (0.91)
Months 7-12	1148	0.58 (0.92)	1157	0.57 (0.75)
**TB treatment OOPs**				
Six months before baseline	1238	15.45 (59.42)	1233	19.71 (119.96)
Months 1-6	1174	22.00 (85.28)	1164	15.77 (42.34)
Months 7-12	1148	5.03 (48.72)	1156	4.36 (30.50)
**Doctor visit OOPs**				
Six months before baseline	1233	61.53 (243.17)	1227	63.21 (199.10)
Months 1-6	1173	27.49 (238.38)	1158	22.07 (216.35)
Months 7-12	1148	13.28 (84.58)	1157	19.07 (162.71)
**Hospital stay OOPs**				
Six months before baseline	1237	6.91 (101.21)	1231	3.01 (28.19)
Months 1-6	1173	16.65 (200.58)	1164	11.72 (220.92)
Months 7-12	1148	17.20 (460.84)	1157	5.65 (99.21)

Productivity loss

Among the respondents, while the mean productivity loss peaked in months 1-6 as expected, it was higher than expected in the six months before baseline, most prominently reflected by productivity loss due to participants' sick leave (Table 2). This might correspond with productivity loss due to companion to TB clinic in the six months before baseline, which was consistent with participants' OOPs for TB clinic during the same period.

Table 2 Mean (SD) productivity loss of companion to TB clinic, doctor, and participants' sick leave, by arm

	**Cytisine (n=1239)**		**Placebo (n=1233)**	
	**n**	**Mean (SD) PPP US$**	**n**	**Mean (SD) PPP US$**
**Companion to TB clinic**				
Six months before baseline	*1232*	*4.62 (9.01)*	*1228*	*4.48 (7.73)*
Month 1 – 6	*1134*	*13.45 (21.55)*	*1127*	*12.43 (19.86)*
Month 7 – 12	*1145*	*2.01 (7.40)*	*1152*	*2.33 (7.19)*
**Companion to doctor**				
Six months before baseline	*1203*	*2.10 (9.15)*	*1196*	*1.87 (6.05)*
Month 1 – 6	*1126*	*3.35 (13.22)*	*1116*	*2.65 (8.44)*
Month 7 – 12	*1143*	*0.37 (2.82)*	*1151*	*0.56 (4.82)*
**Sick leave**				
Six months before baseline	*1230*	*27.14 (73.17)*	*1227*	*23.82 (61.12)*
Month 1 – 6	*1194*	*31.98 (100.27)*	*1171*	*28.52 (107.49)*

For peer review only

	Cytisine (n=1239)		Placebo (n=1233)	
	**n**	**Mean (SD) PPP US$**	**n**	**Mean (SD) PPP US$**
Month 7 – 12	*1163*	*3.62 (18.24)*	*1160*	*5.14 (28.21)*

Quality-adjusted life years

In the EQ-5D-5L descriptive system, the domains with least proportion of respondents scoring no problem were Pain/Discomfort and Anxiety/Depression at all three time points although the proportion increased after baseline (Table 3).

Table 3 Number and percentage of respondents scoring five levels of each domain of EQ-5D-5L, by arm and time point

Domains	Mobility		Self-care		Usual activities		Pain/ Discomfort		Anxiety/ Depression	
	Cytisine	Placebo	Cytisine	Placebo	Cytisine	Placebo	Cytisine	Placebo	Cytisine	Placebo
Baseline										
1	731	746	985	993	655	654	413	426	407	411
	59%	61%	79%	81%	53%	53%	33%	35%	33%	33%
2	315	291	190	163	380	373	447	462	453	463
	25%	24%	15%	13%	31%	30%	36%	38%	37%	38%
3	140	143	49	58	133	146	250	227	232	231
	11%	12%	4%	5%	11%	12%	20%	18%	19%	19%
4	50	51	12	16	55	52	114	104	112	98
	4%	4%	1%	1%	4%	4%	9%	8%	9%	8%
5	3	2	3	1	14	8	14	13	33	29
	0%	0%	0%	0%	1%	1%	1%	1%	3%	2%
Total	1239	1233	1239	1231	1237	1233	1238	1232	1237	1232
Six months										
1	985	992	1077	1078	945	960	753	778	818	829
	86%	88%	94%	95%	83%	85%	66%	69%	72%	73%
2	119	116	56	44	171	147	364	325	287	260
	10%	10%	5%	4%	15%	13%	32%	29%	25%	23%
3	25	13	7	8	19	18	19	20	28	29
	2%	1%	1%	1%	2%	2%	2%	2%	2%	3%
4	12	10	2	2	5	5	6	7	8	12
	1%	1%	0%	0%	0%	0%	1%	1%	1%	1%
5	2	1	1	0	3	1	1	1	2	1
	0%	0%	0%	0%	0%	0%	0%	0%	0%	0%
Total	1143	1132	1143	1132	1143	1131	1143	1131	1143	1131
12 months										
1	994	1020	1059	1082	968	996	755	780	826	833
	90%	91%	96%	97%	88%	89%	69%	70%	75%	75%
2	86	75	33	24	115	101	299	284	226	238
	8%	7%	3%	2%	10%	9%	27%	26%	21%	21%
3	11	12	6	2	12	10	35	34	33	28

For peer review only

	1%	1%	1%	0%	1%	1%	3%	3%	3%	3%
4	8	6	3	4	4	5	9	13	8	13
	1%	1%	0%	0%	0%	0%	1%	1%	1%	1%
5	3	2	1	1	3	1	3	2	4	1
	0%	0%	0%	0%	0%	0%	0%	0%	0%	0%
Total	1102	1115	1102	1113	1102	1113	1101	1113	1097	1113

Levels for each domain: 1=no problem, 2=slight problem, 3=moderate problem, 4=severe problem, 5=extreme problem/inability

Table 4 shows mean EQ-5D-5L utility and VAS among observed cases at baseline, 6 and 12 months follow-ups and QALYs over 6 and 12 months period. Mean utility in the cytisine arm appeared to be consistently lower than in the placebo arm at all timepoints though the difference was small. The mean QALYs were therefore lower in the cytisine arm than in the placebo arm. However, it should be noted, only those who had data on all relevant timepoints were included in calculating QALYs. The EQ-5D VAS showed a similar pattern where both arms began at similar level but in the cytisine arm, the observed cases scored slightly lower than those in the placebo arm in the follow-ups.

Table 4 Mean (SD) EQ-5D-5L utility, EQ-5D VAS and QALYs, by arm

	**Cytisine (n=1239)**		**Placebo (n=1233)**	
	**n**	**Mean (SD)**	**n**	**Mean (SD)**
**Utility**				
Baseline	1234	0.754 (0.133)	1229	0.759 (0.130)
6 months	1179	0.825 (0.165)	1164	0.831 (0.161)
12 months	1144	0.822 (0.189)	1149	0.829 (0.176)
**QALYs**				
Over 6 months	1174	0.394 (0.056)	1160	0.397 (0.054)
Over 12 months	1129	0.805 (0.134)	1122	0.810 (0.128)
**VAS**				
Baseline	1239	53.5 (15.4)	1233	53.5 (16.0)
6 months	1179	80.5 (20.3)	1165	81.3 (19.8)
12 months	1150	84.0 (21.8)	1156	84.7 (20.7)

Cost-utility analysis by country

The mean costs of smoking cessation and healthcare services in the six months before baseline were PPP US$18.33 (SE PPP US$3.65) in Pakistan and PPP US$5.40 (SE PPP US$1.65) in Bangladesh in the cytisine arm. In the placebo arm, the mean costs of these two types of services were PPP US$16.35 (SE PPP US$3.55) in Pakistan and PPP US$3.72 (SE PPP US$0.55) in Bangladesh.

The mean costs of intervention were PPP US$74.37 (SE PPP US$0.68) in the cytisine arm and PPP US$15.84 (SE PPP US$0.03) in the placebo arm in Pakistan. The mean costs of intervention were PPP US$52.10 (SE PPP US$0.13) in the cytisine arm and PPP US$10.23 (SE PPP US$0.00) in the placebo arm in Bangladesh.

The mean costs of TB treatment in the two arms were on a similar level within each country, over PPP US$400 in Pakistan and over PPP US$200 in Bangladesh. The mean costs of doctor visits were very similar between arms in Bangladesh, but they were slightly higher in the cytisine arm in Pakistan (PPP US$3.17 vs PPP US$2.66). The most prominent difference was in the mean costs of

For peer review only - http://bmjopen.bmj.com/site/about/guidelines.xhtml

hospital stay. In Pakistan, the mean costs of hospital stay were considerably higher in the cytisine arm (PPP US$78.12 [SE PPP US$19.80]) than in the placebo arm (PPP US$32.70 [SE PPP US$9.76]). On the contrary, in Bangladesh, the mean costs of hospital stay in the placebo arm (PPP US$7.35 [SE PPP US$3.82]) were over twice as high as in the cytisine arm (PPP US$3.07 [SE PPP US$1.62]). The mean costs of smoking cessation services were not different between arms within each country. However, there were nearly null costs incurred in Pakistan.

Upon further investigation, more participants had hospital stays in Pakistan than in Bangladesh, regardless of which arm they were in. Among participants who incurred hospital stay costs over the six months post-randomisation, not only did the cytisine arm in Pakistan have more participants admitted to hospital but also showed a few potential outliers (Figure 1). This was in contrast with the placebo arm in Pakistan and both arms in Bangladesh.

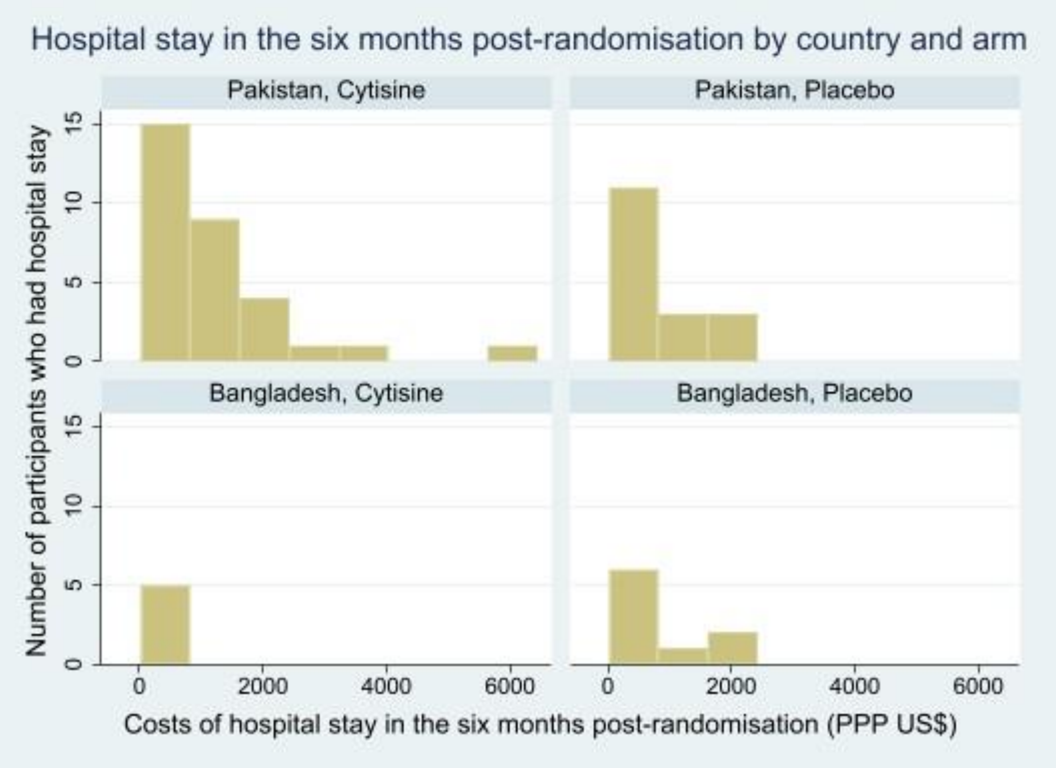

Figure 1 Distribution of costs of hospital stay among those who incurred this cost, by country and arm

Whilst the mean utility was higher in Pakistan than in Bangladesh, the mean utility in both arms showed a relatively gradual and small increase from baseline to six months (Figure 2). In contrast, the mean utility at baseline was much lower in Bangladesh than in Pakistan but it increased more sharply to a similar level in the cytisine arm and a higher level in the placebo arm.

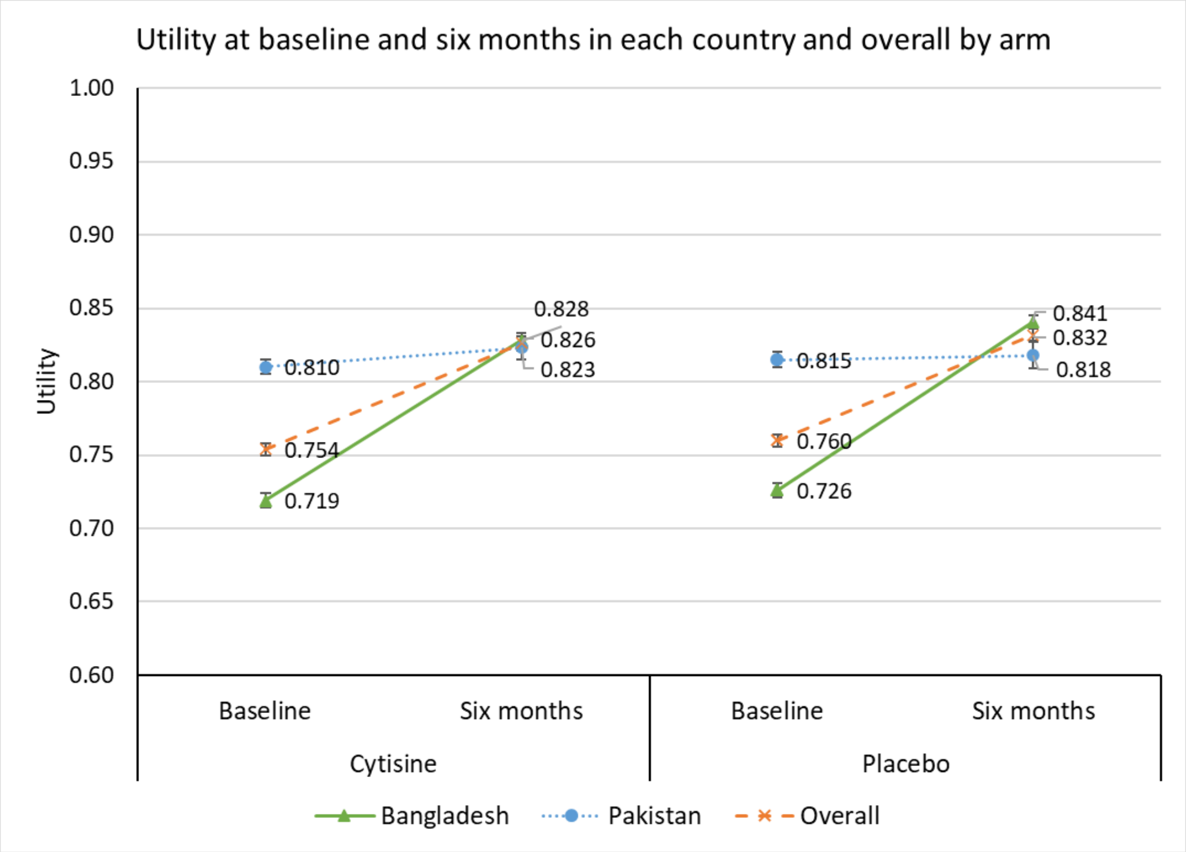

Figure 2 Mean utility at baseline and six months by country and by arm

Adjusting for costs of healthcare and smoking cessation services in the six months before baseline, age, gender, with sites as random effect, the incremental costs over the six months post randomisation were PPP US$108.46 (95%CI PPP US$69.69 to PPP US$157.88) in Pakistan and PPP US$37.06 (95% CI PPP US$28.12 to PPP US$43.85) in Bangladesh (Table 5). Adjusting for utility at baseline, age, gender, with sites as random effect, the incremental QALYs were 0.001 (95% CI -0.004 to 0.008) in Pakistan and -0.003 (95% CI -0.006 to 0.000) in Bangladesh. Therefore, in Pakistan, the ICER was calculated in at PPP US$108,464 per QALY and in Bangladesh, the cytisine arm was dominated by the placebo arm (the cytisine arm being more costly but less effective). Figure 3 shows the uncertainty surrounding the ICERs estimated using bootstrap technique. For Bangladesh, 96% (4794/5000) of the bootstrapped replicates fell in the north-west quadrant of the CEP, where the intervention was more costly but less effective in terms of QALYs. This supports the point estimate that the cytisine arm was dominated by the placebo arm. For Pakistan, 71% (3568/5000) of the bootstrapped replicates fell in the north-east quadrant of the CEP, where the intervention was more costly and more effective in terms of QALYs. The rest fell in the north-west quadrant, indicating a more costly but less effective intervention. According to the estimate made by Woods et al., the willingness-to-pay (WTP) threshold for Pakistan was PPP US$314 to PPP US$2146 per QALY in 2013 [13]. Converting to Pakistan Rupees in 2013 then inflating using consumer price index to 2018 [5 14], the estimated WTP in Pakistan was PPP US$356 to PPP US$2431 per QALY. Represented by the red line in Figure 3, it was apparent that none of the estimates fell under the upper boundary of the WTP (i.e. not cost-effective), same as the point estimate of PPP US$108,464 per QALY. The probability of the cytisine intervention being cost-effective was 0% throughout a wide range of WTP values in both

countries, the CEACs were therefore not presented. By these results, the cytisine intervention was unlikely to be cost-effective, comparing with placebo, in either Pakistan or Bangladesh.

Table 5 Cost-utility analysis results by country (1 PPPUS$ = 30.9 Bangladeshi Taka = 29.3 Pakistani Rupees)

Costs (PPP US$) Mean (SE)	Pakistan		Bangladesh	
	**Cytisine (n=476)**	**Placebo (n=469)**	**Cytisine (n=763)**	**Placebo (n=764)**
Intervention	74.37 (0.68)	15.84 (0.03)	52.10 (0.13)	10.23 (0.00)
TB treatment	421.30 (5.43)	412.97 (5.84)	232.69 (0.78)	233.59 (0.61)
Doctor visit	3.17 (0.82)	2.66 (0.65)	3.50 (0.29)	3.37 (0.29)
Hospital stay	78.12 (19.80)	32.70 (9.76)	3.07 (1.62)	7.35 (3.82)
Smoking cessation	0.00 (0.00)	0.00 (0.00)	0.74 (0.06)	0.71 (0.03)
**Overall total for six months**	**576.96 (20.65)**	**464.16 (11.81)**	**292.07 (1.84)**	**255.28 (3.88)**
Adjusted incremental costs	108.46 (95%CI 69.69 to 157.88)		37.06 (95% CI 28.12 to 43.85)	
**QALYs**	**0.408 (0.002)**	**0.408 (0.003)**	**0.387 (0.002)**	**0.392 (0.002)**
Adjusted incremental QALYs	0.001 (95% CI -0.004 to 0.008)		-0.003 (95% CI -0.006 to 0.000)	
ICER	108,464 per QALY (uncertainty see Figure 3)		Cytisine dominated by placebo (uncertainty see Figure 3)	

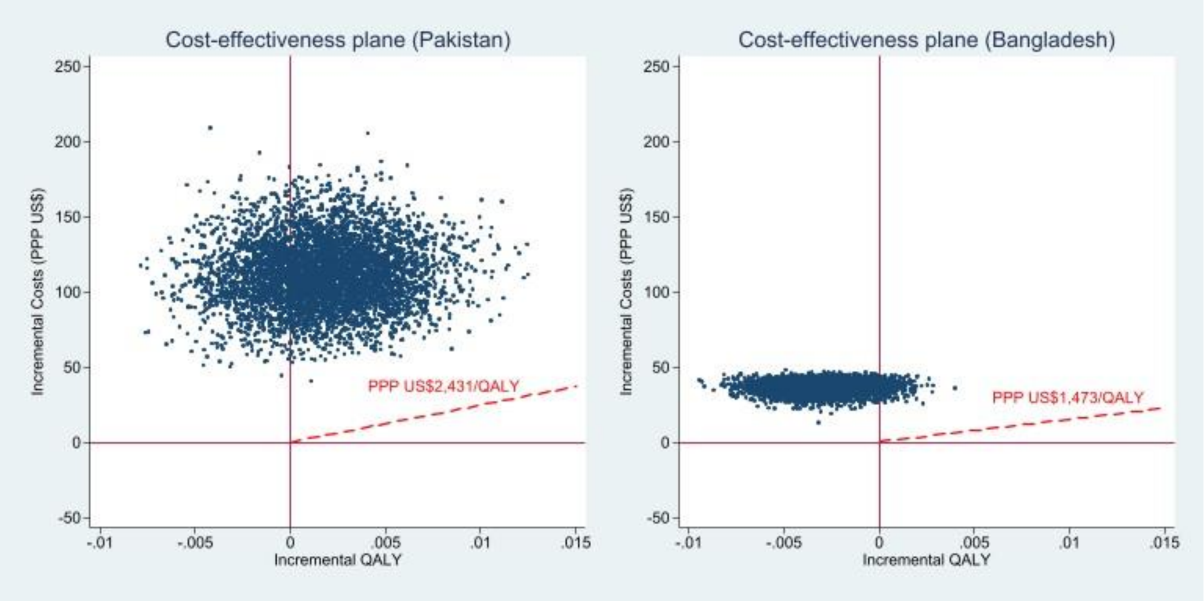

Figure 3 Cost-effectiveness plane of cost-utility analysis results by country

References

1. Bangladesh Bureau of Statistics. Report on Labour Force Survey (LFS) 2016-17, 2018.
2. Pakistan Bureau of Statistics. Labour Force Survey 2017-18, 2018.
3. World Health Organization. Global Tuberculosis report 2018 - TB data: WHO, 2018.
4. Gomez GB, Dowdy DW, Bastos ML, et al. Cost and cost-effectiveness of tuberculosis treatment shortening: a model-based analysis. *BMC Infect Dis* 2016;16(1):726. doi: 10.1186/s12879-016-2064-3 [published Online First: 2016/12/03]
5. The World Bank. DataBank World Development Indicators, 2019.
6. International Monetary Fund. National Currency per U.S. Dollar, period average. Inernational Financial Statistics: International Monetary Fund (IMF), 2019.
7. World Health Organization. Tobacco control country profile Pakistan. Tobacco control country profiles, 2017.
8. World Health Organization. Tobacco control country profile Bangladesh. Tobacco control country profiles, 2017.
9. National Institute for Health and Care Excellence. Putting NICE guidance into practice - Resource impact report: Stop smoking interventions and services (NG92), 2018.
10. Curtis LA, Burns A. Unit Costs of Health and Social Care 2018. Kent: University of Kent, 2018.
11. Economic Analysis and Evaluation Team. Country-specific inpatient and outpatient estimates in 2010 currency: World Health Organisation, 2010.
12. International Monetary Fund. Prices, Consumer Price Index, All items, Index. International Financial Statistics (IFS): International Monetary Fund (IMF), 2019.
13. Woods B, Revill P, Sculpher M, et al. Country-Level Cost-Effectiveness Thresholds: Initial Estimates and the Need for Further Research. *Value Health* 2016;19(8):929-35. doi: 10.1016/j.jval.2016.02.017 [published Online First: 2016/12/19]
14. International Monetary Fund (IMF). Prices, Consumer Price Index, All items, Index. International Financial Statistics (IFS): International Monetary Fund (IMF), 2019.

For peer review only

TB & Tobacco **CASE REPORT FORM - Visit (DAY 0)**

Site ID: ☐☐ **Trial Number:** ☐☐☐☐☐☐ **Date of Completion:** ☐☐ / ☐☐ / ☐☐☐☐
Day Month Year

Section V

ECONOMIC EVALUATION *(This section is about the patient's wider health care use in the past six months, unless stated as TB-specific, this is for any illness.)*

Please exclude care provided by the trial intervention in your answers to these questions.

All costs should be specified in local currency, please round all costs up to the nearest whole number.

Enter a number for each item, if none, enter "0" (zero).

Please use this information to guide you if the patient gives estimates-

For daily visit: One week= 7 days, one month= 30 days, three months= 90 days, six months= 180 days.
For weekly visit per month= 4 times.

(i.e "I visited a centre daily for 6 months" would be 180 times)

1. Have you visited a TB clinic in the past six months?
(please exclude current visit and include visits to diagnostic centres if separate from clinics)

☐ Yes ☐ No (go to Q2)

If 'Yes'

a. How many times have you visited a public/voluntary TB clinic? ☐☐☐

b. How many times have you visited a private TB clinic? ☐☐☐

In **total** how much did you pay in the past six months (for consultation, diagnostics, procedures, drugs)? *(in local currency)* ☐☐☐☐☐☐

c. How much did you usually pay for your own travel per visit? *(in local currency)* ☐☐☐☐☐☐

d. On how many of these visits were you accompanied by a friend/relative? ☐☐☐

e. How much time in **total** did it usually take per visit *(travel, waiting, procedure)*? ☐☐ hours ☐☐ minutes

For peer review only - http://bmjopen.bmj.com/site/about/guidelines.xhtml

TB & Tobacco Trial Number: ☐☐☐☐☐☐

2. Have you visited a doctor in the past six months (*for any illness and exclude TB clinic visits recorded in Q1*)?

☐ Yes ☐ No (go to Q3)

If 'Yes'

a. How many times have you visited a public/voluntary doctor? ☐☐☐

In the past six months, in **total** how much did you pay for public/voluntary visits *(for consultation, diagnostics, procedure, drugs)*? *(in local currency)* ☐☐☐☐☐☐

b. How many times have you visited a private doctor? ☐☐☐

In the past six months, in **total** how much did you pay for private visits *(for consultation, diagnostics, procedure, drugs)*? *(in local currency)* ☐☐☐☐☐☐

c. How much time did you usually spend with the doctor per visit? ☐☐ hours ☐☐ minutes

d. How much did you usually pay for your own travel per visit? *(in local currency)* ☐☐☐☐☐☐

e. On how many of these visits were you accompanied by a friend/relative etc.? ☐☐☐

f. How much time in **total** did it usually take per visit *(travel, waiting, procedure)*? ☐☐ hours ☐☐ minutes

3. Have you been admitted to hospital in the past six months (*for any illness*)?

☐ Yes ☐ No (go to Q4)

If 'Yes'

a. How many nights were you in a public/voluntary hospital? ☐☐☐

In **total** how much did you pay in the past six months at public/voluntary hospitals *(for consultation, diagnostics, procedures, drugs, overnight stay)*? *(in local currency)* ☐☐☐☐☐☐

b. How many nights were you in a private hospital? ☐☐☐

In **total** how much did you pay in the past six months at private hospitals *(for consultation, diagnostics, procedures, drugs, overnight stay)*? *(in local currency)* ☐☐☐☐☐☐

c. How much did you usually pay for your own travel per visit? *(in local currency)* ☐☐☐☐☐☐

For peer review only - http://bmjopen.bmj.com/site/about/guidelines.xhtml

TB & Tobacco Trial Number:

4. Have you received any help to stop smoking in the past six months? *(please exclude the behavioural support session immediately before joining the trial, the session provided by the trial and any medication provided by the trial)*

☐ Yes (go to Q5) ☐ No (go to Q6)

5. How many times in the past six months have you *(this question is only about smoking cessation)*: Enter a number for each item, if none enter '0' (zero).

	Number of times	Amount spent out of pocket *(in local currency)*
Had help or advice about smoking from a public/government clinic/hospital?		
Had help or advice about smoking from a private clinic/hospital?		
Attended a group or single counselling session on smoking at a public/voluntary clinic?		
Attended a group or single counselling session on smoking at a private clinic/hospital?		
Been given a prescription for nicotine patches?		
Been given a prescription for an alternative form of NRT? (such as gum, lozenge, inhaler, etc)		
Bought a refill for an electronic cigarette?		
Been given a prescription for Zyban (Bupropion)?		
Been given a prescription for Champix (Varenicline)?		
Received any traditional medicine? (Hakeem, Homeopathic, Unani etc.)		
Other: please describe:		

For peer review only

For peer review only - http://bmjopen.bmj.com/site/about/guidelines.xhtml

TB & Tobacco

Trial Number: | | | | | | |

6. Have you received any medications for TB in the past six months?

☐ Yes (go to Q7) ☐ No (go to Q8)

7. Please detail below the medications for TB related illness in the past six months? *(Use the colour of the packets to indicate each medication)*

(If patient answers not in days: one week= 7 days, one month= 30 days, three months= 90 days, six months= 180 days etc.)

Anti-TB medication	Number of tablets per day	Duration receiving tablets (days)
Fixed-dose combination (4 drugs) *(R-150mg/H-75mg/E-275mg/P-400mg)*		
Fixed-dose combination (2 drugs) *(R-150mg/H-75mg)*		

8. Do you have a paid job? *(include self-employed and employed) (Please tick one only)*

☐ I have a full time job (go to Q9)

☐ I have a part time job (go to Q9)

☐ I do not have a job (go to Q10)

9. Have you been off work sick in the past six months *(for any illness)*?

☐ Yes

☐ No (go to Q10)

If 'Yes' how many days were you off work sick in the last six months? | | | |

10. Usually how much did you spend **per day** on tobacco over the past six months? *(In local currency)* | | | | | | |

For peer review only

TB & Tobacco Trial Number:

EURO QOL

This section asks about your health in general.
Under each heading, please tick the ONE box that best describes your health TODAY.

MOBILITY

I have no problems in walking about ☐
I have slight problems in walking about ☐
I have moderate problems in walking about ☐
I have severe problems in walking about ☐
I am unable to walk about ☐

SELF-CARE

I have no problems washing or dressing myself ☐
I have slight problems washing or dressing myself ☐
I have moderate problems washing or dressing myself ☐
I have severe problems washing or dressing myself ☐
I am unable to wash or dress myself ☐

USUAL ACTIVITIES *(e.g. work, study, housework, family or leisure activities)*

I have no problems doing my usual activities ☐
I have slight problems doing my usual activities ☐
I have moderate problems doing my usual activities ☐
I have severe problems doing my usual activities ☐
I am unable to do my usual activities ☐

PAIN/DISCOMFORT

I have no pain or discomfort ☐
I have slight pain or discomfort ☐
I have moderate pain or discomfort ☐
I have severe pain or discomfort ☐
I have extreme pain or discomfort ☐

ANXIETY/DEPRESSION

I am not anxious or depressed ☐
I am slightly anxious or depressed ☐
I am moderately anxious or depressed ☐
I am severely anxious or depressed ☐
I am extremely anxious or depressed ☐

UK (English) © 2009 EuroQol Group. EQ-5D™ is a trade mark of the EuroQol Group

For peer review only - http://bmjopen.bmj.com/site/about/guidelines.xhtml

For peer review only

TB & Tobacco

Trial Number: ☐☐☐☐☐☐

The best health
you can imagine

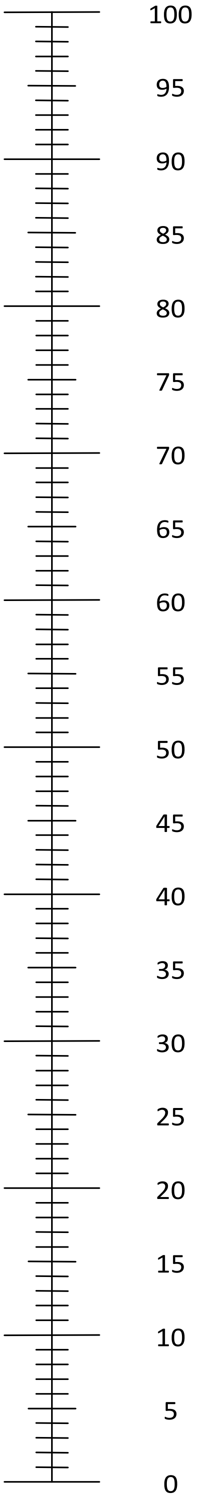

The worst health
you can imagine

- We would like to know how good or bad your health is TODAY.
- The scale is numbered from 0 to 100.
- 100 means the best health you can imagine.

  0 means the worst health you can imagine.
- Mark an X on the scale to indicate how your health is TODAY.
- Now, please write the number you marked on the scale in the box below.

YOUR HEALTH TODAY = ☐☐☐

For peer review only

UK (English) © 2009 EuroQol Group. EQ-5D™ is a trade mark of the EuroQol Group

Please dispense medication for 1 week. Instruct the participant to come back for follow up coinciding with their quit date and also to bring the blister packets and the 'dosing schedule card'.

Thank you for your time!

Send data

For peer review only - http://bmjopen.bmj.com/site/about/guidelines.xhtml

Supplementary tables

Table S1 Average hourly wage by occupation in Pakistan and Bangladesh

	Average hourly wage (PPP US$) [1-3]					
	Bangladesh			Pakistan		
Occupation	Male	Female	Total	Male	Female	Total
Managers	5.68	5.13	5.62	9.67	0.84	9.57
Professionals	4.25	3.93	4.13	6.06	3.84	5.30
Technicians and Associate Professionals	3.35	3.21	3.32	4.69	3.27	4.50
Clerical support workers	2.56	2.33	2.53	4.69	3.16	4.66
Service and Sales workers	1.88	1.76	1.86	2.85	2.37	2.83
Skilled Agricultural, forestry and fisheries	1.50	1.24	1.46	3.03	0.98	2.96
Craft and Related Trades workers	1.69	1.55	1.65	3.00	0.89	2.67
Plant and Machine Operators, and Assembler	1.91	1.77	1.89	2.96	1.95	2.95
Elementary Occupations	1.38	1.15	1.32	2.39	1.11	2.15
Overall	2.14	1.93	2.09	3.35	2.00	3.15

For peer review only

Table S2 Number and proportion of missing values of variables by arm

	Cytisine (n=1239)		Placebo (n=1233)	
Variables	Number of missing values	Proportion of missing values	Number of missing values	Proportion of missing values
Cost of behavioural support	6	0%	7	1%
Cost of TB treatment	102	8%	103	8%
Cost of doctor visit at d0	0	0%	1	0%
Cost of doctor visit at m6	61	5%	67	5%
Cost of doctor visit at m12	89	7%	76	6%
Cost of hospital stay at d0	2	0%	2	0%
Cost of hospital stay at m6	62	5%	67	5%
Cost of hospital stay at m12	89	7%	76	6%
Cost of smoking cessation at d0	0	0%	0	0%
Cost of smoking cessation at m6	63	5%	69	6%
Cost of smoking cessation at m12	103	8%	89	7%
OOP on TB treatment at d0	1	0%	0	0%
OOP on TB treatment at m6	63	5%	69	6%
OOP on TB treatment at m12	89	7%	77	6%
OOP on smoking cessation at d0	3	0%	3	0%
OOP on smoking cessation at m6	146	12%	153	12%
OOP on smoking cessation at m12	127	10%	118	10%
OOP on doctor visit at d0	6	0%	6	0%
OOP on doctor visit at m6	64	5%	75	6%
OOP on doctor visit at m12	89	7%	76	6%
OOP on hospital stay at d0	2	0%	2	0%
OOP on hospital stay at m6	64	5%	69	6%
OOP on hospital stay at m12	89	7%	76	6%
OOP on tobacco products d0	10	1%	9	1%
OOP on tobacco products m6	60	5%	67	5%
OOP on tobacco products m12	89	7%	76	6%
Productivity loss of company for TB treatment at d0	7	1%	5	0%
Productivity loss of company for TB treatment at m6	103	8%	106	9%
Productivity loss of company for TB treatment at m12	92	7%	81	7%
Productivity loss of company for doctor at d0	36	3%	37	3%
Productivity loss of company for doctor at m6	111	9%	117	9%
Productivity loss of company for doctor at m12	94	8%	82	7%
Productivity loss of sick leave at d0	9	1%	6	0%
Productivity loss of sick leave at m6	44	4%	62	5%

For peer review only

	Cytisine (n=1239)		Placebo (n=1233)	
Variables	Number of missing values	Proportion of missing values	Number of missing values	Proportion of missing values
Productivity loss of sick leave at m12	75	6%	73	6%
EQ-5D-5L at d0				
1 Mobility	0	0%	0	0%
2 Self-care	0	0%	2	0%
3 Usual activities	2	0%	0	0%
4 Pain and discomfort	1	0%	1	0%
5 Anxiety or depression	2	0%	1	0%
EQ-5D-5L at m6				
1 Mobility	60	5%	67	5%
2 Self-care	60	5%	67	5%
3 Usual activities	60	5%	68	6%
4 Pain and discomfort	60	5%	68	6%
5 Anxiety or depression	60	5%	68	6%
EQ-5D-5L at m12				
1 Mobility	89	7%	76	6%
2 Self-care	89	7%	78	6%
3 Usual activities	89	7%	78	6%
4 Pain and discomfort	90	7%	78	6%
5 Anxiety or depression	94	8%	78	6%
VAS at d0	0	0%	0	0%
VAS at m6	60	5%	68	6%
VAS at m12	89	7%	77	6%
TB score at d0	0	0%	0	0%
TB score at m6	60	5%	66	5%

For peer review only

Table S3 Logistic regression for missingness of costs, OOPs, productivity loss and outcomes on arm and baseline covariates

Missing on:	Allocation	Age	Country
Cost of TB treatment	1.02 (0.76-1.35)	1.02 (1.01-1.03)	**0.26 (0.19-0.36)***
Cost of doctor visit at m6	1.07 (0.75-1.53)	1.00 (0.99-1.01)	**0.15 (0.10-0.24)***
Cost of doctor visit at m12	0.83 (0.61-1.14)	1.00 (0.99-1.01)	**0.17 (0.12-0.25)***
Cost of hospital stay at m6	1.05 (0.74-1.50)	1.00 (0.99-1.01)	**0.16 (0.11-0.24)***
Cost of hospital stay at m12	0.83 (0.61-1.14)	1.00 (0.99-1.01)	**0.17 (0.12-0.25)***
Cost of smoking cessation at m6	1.07 (0.76-1.52)	1.00 (0.99-1.01)	**0.17 (0.11-0.26)***
Cost of smoking cessation at m12	0.84 (0.63-1.13)	1.00 (0.99-1.01)	**0.28 (0.21-0.39)***
OOP on TB treatment at m6	1.07 (0.76-1.52)	1.00 (0.99-1.01)	**0.16 (0.10-0.24)***
OOP on TB treatment at m12	0.84 (0.62-1.15)	1.00 (0.99-1.01)	**0.17 (0.12-0.25)***
OOP on smoking cessation at m6	1.04 (0.82-1.33)	**0.99 (0.98-1.00)***	1.14 (0.89-1.47)
OOP on smoking cessation at m12	0.91 (0.70-1.18)	0.99 (0.98-1.00)	**0.52 (0.40-0.68)***
OOP on doctor visit at m6	1.15 (0.82-1.62)	1.00 (0.99-1.01)	**0.16 (0.10-0.24)***
OOP on doctor visit at m12	0.83 (0.61-1.14)	1.00 (0.99-1.01)	**0.17 (0.12-0.25)***
OOP on hospital stay at m6	1.05 (0.74-1.49)	1.00(0.99-1.01)	**0.16 (0.11-0.24)***
OOP on hospital stay at m12	0.83 (0.61-1.14)	1.00 (0.99-1.01)	**0.17 (0.12-0.25)***
OOP on tobacco products m6	1.09 (0.76-1.55)	1.00 (0.99-1.01)	**0.16 (0.10-0.24)***
OOP on tobacco products m12	0.83 (0.61-1.14)	1.00 (0.99-1.01)	**0.17 (0.12-0.25)***
Productivity loss of company for TB treatment at m6	1.01 (0.77-1.35)	0.99 (0.98-1.00)	**0.55 (0.41-0.73)***
Productivity loss of company for TB treatment at m12	0.86 (0.63-1.16)	1.00 (0.99-1.01)	**0.18 (0.13-0.26)***
Productivity loss of company for doctor at m6	1.04 (0.80-1.37)	0.99 (0.98-1.00)	**0.52 (0.40-0.69)***
Productivity loss of company for doctor at m12	0.85 (0.63-1.15)	1.00 (0.99-1.01)	**0.18 (0.12-0.25)***
Productivity loss of sick leave at m6	1.40 (0.95-2.08)	0.99 (0.97-1.00)	**0.22 (0.14-0.33)***
Productivity loss of sick leave at m12	0.95 (0.68-1.32)	0.99 (0.98-1.00)	**0.27 (0.19-0.38)***
EQ-5D-5L at m6	1.09 (0.76-1.55)	1.00 (0.99-1.01)	**0.16 (0.10-0.24)***
EQ-5D-5L at m12	0.83 (0.61-1.14)	1.00 (0.99-1.01)	**0.17 (0.12-0.25)***
TB score at m6	1.02 (0.72-1.44)	1.00 (0.99-1.01)	**0.18 (0.12-0.26)***

***P<0.05**

For peer review only

References

1. Bangladesh Bureau of Statistics. Report on Labour Force Survey (LFS) 2016-17, 2018.
2. Pakistan Bureau of Statistics. Labour Force Survey 2017-18, 2018.
3. The World Bank. DataBank World Development Indicators, 2019.

For peer review only

CHEERS Checklist

Items to include when reporting economic evaluations of health interventions

The **ISPOR CHEERS Task Force Report**, *Consolidated Health Economic Evaluation Reporting Standards (CHEERS)—Explanation and Elaboration: A Report of the ISPOR Health Economic Evaluations Publication Guidelines Good Reporting Practices Task Force*, provides examples and further discussion of the 24-item CHEERS Checklist and the CHEERS Statement. It may be accessed via the *Value in Health* or via the ISPOR Health Economic Evaluation Publication Guidelines – CHEERS: Good Reporting Practices webpage: http://www.ispor.org/TaskForces/EconomicPubGuidelines.asp

Section/item	Item No	Recommendation	Reported on page No/ line No
**Title and abstract**			
Title	1	Identify the study as an economic evaluation or use more specific terms such as "cost-effectiveness analysis", and describe the interventions compared.	P1 L1-4
Abstract	2	Provide a structured summary of objectives, perspective, setting, methods (including study design and inputs), results (including base case and uncertainty analyses), and conclusions.	P2
**Introduction**			
Background and objectives	3	Provide an explicit statement of the broader context for the study. Present the study question and its relevance for health policy or practice decisions.	P4 L2-23
**Methods**			
Target population and subgroups	4	Describe characteristics of the base case population and subgroups analysed, including why they were chosen.	P4 L35-P5 L7
Setting and location	5	State relevant aspects of the system(s) in which the decision(s) need(s) to be made.	P4 6-12, P5 L3-5
Study perspective	6	Describe the perspective of the study and relate this to the costs being evaluated.	P4 L20-23, P7 L6-11
Comparators	7	Describe the interventions or strategies being compared and state why they were chosen.	P5 L10-17
Time horizon	8	State the time horizon(s) over which costs and consequences are being evaluated and say why appropriate.	P7 L6, P4 L6-8
Discount rate	9	Report the choice of discount rate(s) used for costs and outcomes and say why appropriate.	P7 L11-12
Choice of health outcomes	10	Describe what outcomes were used as the measure(s) of benefit in the evaluation and their relevance for the type of analysis performed.	P6 L23-35, P7 L6
Measurement of effectiveness	11a	*Single study-based estimates:* Describe fully the design features of the single effectiveness study and why the single study was a sufficient source of clinical effectiveness data.	P4 L14-30

For peer review only

	11b	*Synthesis-based estimates:* Describe fully the methods used for identification of included studies and synthesis of clinical effectiveness data.	N/A
Measurement and valuation of preference based outcomes	12	If applicable, describe the population and methods used to elicit preferences for outcomes.	P6 L31-35
Estimating resources and costs	13a	*Single study-based economic evaluation:* Describe approaches used to estimate resource use associated with the alternative interventions. Describe primary or secondary research methods for valuing each resource item in terms of its unit cost. Describe any adjustments made to approximate to opportunity costs.	P5 L19-P6 L22, Supporting information 1
	13b	*Model-based economic evaluation:* Describe approaches and data sources used to estimate resource use associated with model health states. Describe primary or secondary research methods for valuing each resource item in terms of its unit cost. Describe any adjustments made to approximate to opportunity costs.	N/A
Currency, price date, and conversion	14	Report the dates of the estimated resource quantities and unit costs. Describe methods for adjusting estimated unit costs to the year of reported costs if necessary. Describe methods for converting costs into a common currency base and the exchange rate.	P5 L19-23
Choice of model	15	Describe and give reasons for the specific type of decision-analytical model used. Providing a figure to show model structure is strongly recommended.	N/A
Assumptions	16	Describe all structural or other assumptions underpinning the decision-analytical model.	N/A
Analytical methods	17	Describe all analytical methods supporting the evaluation. This could include methods for dealing with skewed, missing, or censored data; extrapolation methods; methods for pooling data; approaches to validate or make adjustments (such as half cycle corrections) to a model; and methods for handling population heterogeneity and uncertainty.	P6 L38-P7 L36
**Results**			
Study parameters	18	Report the values, ranges, references, and, if used, probability distributions for all parameters. Report reasons or sources for distributions used to represent uncertainty where appropriate. Providing a table to show the input values is strongly recommended.	Table 1, Table S1, Supporting information 1
Incremental costs and outcomes	19	For each intervention, report mean values for the main categories of estimated costs and outcomes of interest, as well as mean differences between the comparator groups. If applicable, report incremental cost-effectiveness ratios.	P8 L15-25, Table 2
Characterising uncertainty	20a	*Single study-based economic evaluation:* Describe the effects of sampling uncertainty for the estimated incremental cost and incremental effectiveness parameters, together with the impact	P8 L28-40, Figure 1-2

For peer review only

		of methodological assumptions (such as discount rate, study perspective).	
	20b	*Model-based economic evaluation:* Describe the effects on the results of uncertainty for all input parameters, and uncertainty related to the structure of the model and assumptions.	N/A
Characterising heterogeneity	21	If applicable, report differences in costs, outcomes, or cost-effectiveness that can be explained by variations between subgroups of patients with different baseline characteristics or other observed variability in effects that are not reducible by more information.	N/A
**Discussion**			
Study findings, limitations, generalisability, and current knowledge	22	Summarise key study findings and describe how they support the conclusions reached. Discuss limitations and the generalisability of the findings and how the findings fit with current knowledge.	P9 L21-P10 L9
**Other**			
Source of funding	23	Describe how the study was funded and the role of the funder in the identification, design, conduct, and reporting of the analysis. Describe other non-monetary sources of support.	P10 L21-24
Conflicts of interest	24	Describe any potential for conflict of interest of study contributors in accordance with journal policy. In the absence of a journal policy, we recommend authors comply with International Committee of Medical Journal Editors recommendations.	P10 L29-37

For consistency, the CHEERS Statement checklist format is based on the format of the CONSORT statement checklist

The **ISPOR CHEERS Task Force Report** provides examples and further discussion of the 24-item CHEERS Checklist and the CHEERS Statement. It may be accessed via the *Value in Health* link or via the ISPOR Health Economic Evaluation Publication Guidelines – CHEERS: Good Reporting Practices webpage: http://www.ispor.org/TaskForces/EconomicPubGuidelines.asp

The citation for the CHEERS Task Force Report is:
Husereau D, Drummond M, Petrou S, et al. Consolidated health economic evaluation reporting standards (CHEERS)—Explanation and elaboration: A report of the ISPOR health economic evaluations publication guidelines good reporting practices task force. Value Health 2013;16:231-50.

For peer review only
